# Understanding the impact of crosslinked PCL/PEG/GelMA electrospun nanofibers on bactericidal activity

**DOI:** 10.1371/journal.pone.0209386

**Published:** 2018-12-20

**Authors:** Mirian Michelle Machado De Paula, Nicole Joy Bassous, Samson Afewerki, Samarah Vargas Harb, Paria Ghannadian, Fernanda Roberta Marciano, Bartolomeu Cruz Viana, Carla Roberta Tim, Thomas Jay Webster, Anderson Oliveira Lobo

**Affiliations:** 1 Faculty of Medical Sciences, State University of Campinas, Campinas, São Paulo, Brazil; 2 Department of Chemical Engineering, Northeastern University, Boston, Massachusetts, United States of America; 3 Department of Chemical Engineering and Koch Institute for Integrative Cancer Research, Massachusetts Institute of Technology, Cambridge, Massachusetts, United States of America; 4 Division of Gastroenterology, Brigham and Women´s Hospital, Harvard Medical School, Boston, Massachusetts, United States of America; 5 Institute of Chemistry, UNESP-São Paulo State University, Araraquara, São Paulo, Brazil; 6 Institute of Science and Technology, Brasil University, São Paulo, SP, Brazil; 7 LIMAV-Interdisciplinary Laboratory for Advanced Materials, PPGCM-Materials Science and Engineering graduate program, UFPI-Federal University of Piauí, Teresina, PI, Brazil; 8 Department of Physics, UFPI-Federal University of Piauí, Teresina, PI, Brazil; 9 Department of Chemistry, Massachusetts Institute of Technology, Cambridge, Massachusetts, United States of America; University of New South Wales, AUSTRALIA

## Abstract

Herein, we report the design of electrospun ultrathin fibers based on the combination of three different polymers polycaprolactone (PCL), polyethylene glycol (PEG), and gelatin methacryloyl (GelMA), and their potential bactericidal activity against three different bacteria *Staphylococcus aureus* (*S*. *aureus*), *Pseudomonas aeruginosa* (*P*. *aeruginosa*), and *Methicillin-resistant Staphylococcus aureus* (MRSA). We evaluated the morphology, chemical structure and wettability before and after UV photocrosslinking of the produced scaffolds. Results showed that the developed scaffolds presented hydrophilic properties after PEG and GelMA incorporation. Moreover, they were able to significantly reduce gram-positive, negative, and MRSA bacteria mainly after UV photocrosslinking (PCL:PEG:GelMa-UV). Furthermore, we performed a series of study for gaining a better mechanistic understanding of the scaffolds bactericidal activity through protein adsorption study and analysis of the reactive oxygen species (ROS) levels. Furthermore, the *in vivo* subcutaneous implantation performed in rats confirmed the biocompatibility of our designed scaffolds.

## Introduction

Microbial infections are major challenges to the public health, and the burden is rapidly growing due to the increase of healthcare cost and antibiotic resistance, in particular with respect to wound healing [1]. Here, a microbial infection at the wound site can severely prolong the healing process, leading to necrosis, sepsis, and even death [2]. The most common pathogenic bacteria found in the hospital environment are *Escherichia coli (E*. *coli)*, *Pseudomonas aeruginosa (P*. *aeruginosa)*, *Staphylococcus aureus* (*S*. *aureus*), *Staphylococcus epidermis (S*. *epidermis)*, and various filamentous fungi as well as yeasts (*i*.*e*., *Candida spp*.) [3–5]. The use of antibiotics can results in an escalating drug resistance in pathogenic microorganisms, which is associated with increased morbidity and mortality and therefore should be avoided when possible [6]. In fact, according to the World Health Organization (WHO), antibiotic resistance is one of the biggest threats to global health. Therefore, the design of materials with the ability to significantly reduce the administration of antibiotics and avoid infections from highly antibiotic-resistant bacteria in the hospital environment are highly desirable. In this context, degradable polymers with tunable mechanical, biological and chemical properties and ease of fabrication can be attractive for aforementioned applications [7]. Through the combination of several biomaterials with inimitable properties, a tailor-made hybrid material can be designed without the use of any complicated fabrication approaches or chemistry. However, one requirement is that such materials are compatible with each other avoiding interference or inhibition due to mismatched chemistry or structure, but instead promote properties synergistically.

Coupled with the above, biomaterial nanotexturing has recently been shown to reduce microorganism attachment and growth. One method for the preparation of ultrathin (fiber with diameters ranging from <3 nm to over 1 μm) biomaterial fibers can be accomplished by employing electrospinning technology [8, 9]. This approach is simple, efficient, reproducible, cost-efficient, and provides high yield [10, 11]. Here, we envision by merging three polymeric biomaterials (polycaprolactone (PCL), polyethylene glycol (PEG) and photocrosslinkable gelatin methacryloyl (GelMA)) for the generation of ultrathin electrospun fibers, bactericidal property can be obtained. These materials were selected based on their unique properties. PCL is one of the most extensively employed polyesters in biomedical and tissue engineering applications due to its biocompatibility, mechanical strength, viscoelasticity and biodegradability [12]. Nevertheless, its hydrophobic property and long degradation time limits its applications. One way to solve such limitations is by merging PCL with other biomaterials to improve its properties such as hydrophilicity, degradability, and controllable mechanical properties [13]. Due data, several strategies have been demonstrated combining PCL with other polymers to obtain partial or total hydrophilicity to open numerous possibilities for biomedical purposes. Furthermore, PEG is biocompatible, hydrophilic and has the ability to reduce protein adsorption [12]. In addition, it is known that a surface resistant to protein adsorption is a pre-requisite for preventing bacteria adhesion [13, 14]. However, it still has limits in that it cannot be used alone to produce mats, but rather needs to be combined with other polymers. Likewise, it have previously been demonstrated that PEG-coated surface can reduce bacterial adhesion highlighting the importance of integrating PEG with other biomaterials [13, 15].

Since the material is intended for biomedical and tissue engineering applications it is extremely important to use biomaterials that promote good interaction with the biological environment. GelMA is a biomaterial that resembles the natural components in the extracellular matrix in native tissues and displays several biological significance properties such as biocompatibility, biodegradability, low antigenicity and promoting cell adhesion due to the RGD (Arg-Gly-Asp) motif. Nevertheless, its poor mechanical properties and fast enzymatic degradation have limited its biomedical applications and in this context, lacks bactericidal properties [16],[17]. Actually, previously gelatin has been reported to be a good source for bacteria growth [18]. However, the resistance of bacteria in contact with GelMa blend has not yet been fully investigated. Furthermore, even though PCL based nanofibers have been investigated regarding their antibacterial activity, the incorporation of antibiotic drugs or silver particles within PCL nanofibers are necessary for promoting antibacterial activity [19],[20],[21]. Therefore, our motivation for the present work is designing a PCL based nanofiber blend with potential antibacterial activity or bactericidal activity without the incorporation of any antibiotic.

Herein, we electrospun a combination of PCL, PEG and GelMA and evaluated the potential of the resulting UV crosslinked scaffolds as bactericidal biomaterial and the understanding about a possible mechanism of reduction using specific biological assays. To the best of our knowledge, this is the first report on the electrospinning of the combination of PCL, PEG, and GelMA, evaluating their bactericidal activity against reduce *S*. *aureus*, *P*. *aeruginosa*, and MRSA growth. In doing so, this study has opened numerous perspectives to use a new bactericidal scaffolding system for a wide range of biomedical and tissue engineering applications.

## Materials and methods

### Materials

The following chemicals were purchased from Sigma-Aldrich (St. Louis, MO, USA): PCL (molecular weight (Mw) 80,000), PEG (Mw 8,000), Gelatin (Type A, 300 bloom from porcine skin), methacrylic anhydride (MA), dimethyl sulfoxide (DMSO), and photoinitiator 2-hydroxy-1-[4-(hydroxyethoxy) phenyl]-2-methyl-1-propanone (Irgacure 2959). Hexafluoroisopropan-2-ol (HFIP) was purchased from Oakhood Chemical. Dulbecco’s phosphate buffered saline (DPBS), trypsin-EDTA (ethylenediaminetetraacetic acid) and penicillin−streptomycin were purchased from Gibco (MD, USA). Alpha-modified Eagle’s medium (Alpha-MEM) was supplied by Invitrogen (Grand Island, NY, USA). HyClone characterized fetal bovine serum (FBS) and pre-cleaned microscope slides were obtained from Fisher Scientific (Waltham, MA, USA). *S*. *aureus*, *P*. *aeruginosa*, and MRSA were acquired from the ATCC strain 12600. The UV source (Omnicure model S2000) was provided by EXPO Photonic Solutions Inc. (Ontario, Canada).

### Procedure for the synthesis of GelMA

**Fig 1:**
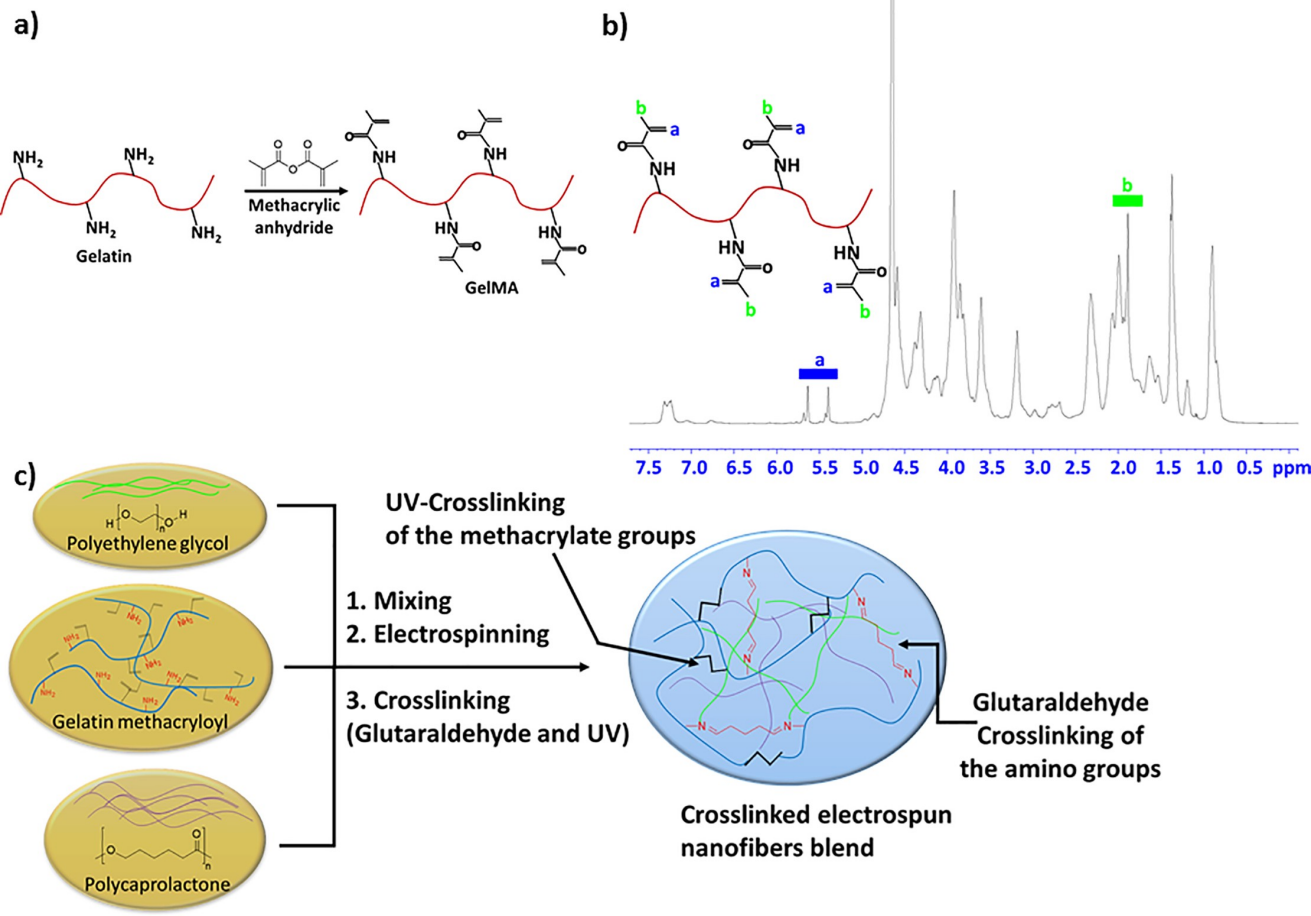
a) The synthetic route for the preparation of gelatin methacryloyl (GelMA). b) ^1^H-NMR of the prepared GelMA identifying the specific groups. c) The fabrication approaches for the preparation of the PCL, PEG and GelMA combined electrospun fiber blend. The blend polymers are entrapped in the crosslinked GelMA through two different covalent bonds. The imine bonds promoted through glutaraldehyde crosslinking and the crosslinking between the double bonds (C = C) in GelMA promoted through UV-crosslinking.

GelMA was prepared following our previously reported protocol (**Fig 1A**) [22]. Shortly, gelatin type A (10 g) in 100 mL PBS was stirred at 50 ^o^C until it was fully dissolved. Afterwards, methacrylate anhydride (3 mL) was added and stirred for additional 2 h. The material was purified through dialysis tubing (12–14 kDa MWCO, spectrum Lab Inc.) for 7 days at 40 ^o^C. Finally after freeze-drying GelMA was obtained as a white solid.

### ^1^H NMR analysis

Prior NMR running, the GelMA samples were dissolved in deuterium oxide (D_2_O) at a concentration of 30 mg/mL and the running was performed at 37 ^o^C. The data were processed using ACD LABS 12.0 software. The degree of substitution of GelMA was determined to 71% by NMR analysis (**Fig 1B**).

### Preparation of the solutions prior electrospinning

First the solution was prepared separately: PCL (0.5 g), PEG (0.5 g) and GelMa (0.5 g) in 5 mL of HFIP. Subsequently, the vials were closed and dissolved under stirring at room temperature for 24 h. For the PCL:PEG samples the mixing of the solutions were realized in the ratio of 80:20, respectively and for the sample PCL:PEG:GelMa the mixing of the solutions were realized in the ratio of 60:30:10, respectively (the selection of the ratios are based on previous publication) [23]. Then, the solutions were placed in a syringe (BD Yale, 3 mL) followed a metal needle tip (Inbras, 23G). The electrospinning was carried out at 17 kV (Nanospinner Machine, Inovenso) as a positive voltage. An aluminum foil collector plate (20 cm x 20 cm square plate) was used as the anode, a needle-collector distance of 10 cm was used, and 1 mL/h was used as the solution flow rate (Harvard, PHD 2000). The temperature and humidity were controlled at 21–23°C and 44–54%, respectively.

### Photocrosslinking of the produced scaffolds

The electrospun mats was first immersed in a glutaraldehyde solution (10 mL/500 mL ethanol) overnight. This step ensure stabilization of the nanofibers through crosslinking of the amino groups in the gelatin forming covalently bonded imine bonds [24]. Subsequently, the unreacted glutaraldehyde was quenched by extensively washing with a solution of glycine (3.75 g in 250 mL of DI water) [25]. The photocrosslinking solution was prepared by adding 1.0 g of the photoinitiator (Igacure 2959) to 10 mL of ethanol in the absence of the light and stirred until completely dissolved. Subsequently, the uncrosslinked mats were immersed in an ethanol solution containing photoinitiator (10 wt%) for 2 h and then exposed to a 365-nm UV light (OminiCure-2000 Series) at a 10-cm working distance for 10 min. Subsequently, the crosslinked mats were washed 3 times with ethanol to remove excess photoinitiator. Afterwards, the mats were washed with DI water and dried under vacuum. **Fig 1C** illustrates a possible mechanism of the PCL:PEG:GelMA interaction after photocrosslinking.

### Characterization of the produced scaffolds

A scanning electronic microscope (FEI Quanta 200, 3 kV) was used to analyze the scaffold morphology. A thin gold layer was evaporated onto all scaffold surfaces before analysis to improve image acquisition.

Fourier transform infrared spectrometry (FTIR, Spotlight-400, Perkin Elmer FTIR Imaging System) was used to analyze the chemical groups in the biomaterial before and after photocrosslinking under a controlled atmosphere and transmittance mode. The spectra were collected at regions between 4000–400 cm^-1^ and were analyzed in three different regions. The regions of interest were analyzed, and their vibrational modes indexed.

For wettability analysis, the contact angle (CA) between the surface of the scaffold and a DI water or diiodomethane drop was monitored in the dynamic mode, using a contact angle device (Krüss, Model DSA 100). Before analysis, a careful calibration was performed in accordance to the equipment manual and all the scaffolds were fixed on a Teflon base. Then, DI water and diiodomethane (2 μL) were dropped onto the scaffold surfaces and measured after 1 s. The atmosphere and humidity (~ 60%) were controlled during all the measurements. The assay was performed in triplicate.

The surface energy composed of polar and dispersive components of the samples was evaluated by measuring CA. The interfacial tension between two condensed phases can be determined by Young’ Eq 1 [26], according to which

$$cos\theta \gamma_{LV}=\gamma_{SV}-\gamma_{SL},$$ (1)

where θ is the measured contact angle between liquid and solid, and *γ*_*LV*_, *γ*_*SV*_, and *γ*_*SL*_ are the interfacial energies of the liquid/vapor, solid/vapor, and solid/liquid interfaces, respectively. This equation can be rewritten as the Young-Duprè Eq 2:

$$W_{a}=\gamma_{LV}(1+cos\theta )=\gamma_{SV}-\gamma_{SL}$$ (2)

where *W*_*a*_ is the adhesion energy per unit area of the solid and liquid surfaces. In the general form of Eqs 1 and 2 can be written (Eq 3):

$$\gamma_{LV}(1+cos\theta )=2\sqrt{\gamma_{L}^{p}\gamma_{S}^{p}}+2\sqrt{\gamma_{L}^{D}\gamma_{S}^{D}}$$ (3)

where *γ*^*p*^_*L*_ and *γ*^*p*^_*S*_ are the polar components of the surface energy of liquid and solid phases, respectively, and *γ*^*D*^_*L*_ and *γ*^*D*^_*S*_ are the dispersive component of the surface energy of the liquid and solid phases, respectively. Because *γ*^*D*^_*L*_ and *γ*^*p*^_*L*_ have been published for many liquids, it is possible to approximate *γ*^*D*^_*S*_ and *γ*^*p*^_*S*_ from a single measurement of θ with Eq 3. Therefore, by measuring the CA of two different liquids (distilled water and diiodomethane) with well-known polar and dispersive components of surface energy (Table 1), Eq 3 can be solved to determine the polar and dispersive components of the surface energy of the materials [27, 28]. The liquid was dropped automatically by a computer-controlled system.

**Table 1:** Test liquids and their surface tension components [27].

Surface tension data (mN/m)	*γ*^D^_L_	*γ*^p^_L_	*γ*_LV_
Water	21.8	51.0	72.8
Diiodomethane	50.8	0.0	50.8

The process of adhesion and spreading of bacteria were firstly calculated thermodynamically from a liquid suspension onto solid substrata can be described by the Eq 4 [29]:

$$\Delta F_{Adh}=\gamma_{BS}-\gamma_{BL}-\gamma_{SL},$$ (4)

where Δ*F*_Adh_ is the interfacial free energy of adhesion, *γ*_*B*S_ is the bacteria-solid substratum interfacial free energy, *γ*_*BL*_ the bacteria-liquid interfacial free energy, and *γ*_*SL*_ is the solid-liquid interfacial free energy, respectively. They can be calculated by using CA data and the van Oss acid-base approach [30–32].

The following Eq 5 was used to determine the interfacial energy of cell adhesion to a solid surface [30–32]:

$$\Delta F_{Adh}=2(\begin{array}{l} \sqrt{\gamma_{S}^{LW}\gamma_{L}^{LW}}+\sqrt{\gamma_{S}^{p}\gamma_{L}^{D}}+\sqrt{\gamma_{S}^{D}\gamma_{L}^{p}}\\ +\sqrt{\gamma_{B}^{LW}\gamma_{L}^{LW}}+\sqrt{\gamma_{B}^{p}\gamma_{L}^{D}}+\sqrt{\gamma_{B}^{D}\gamma_{L}^{p}}\\ -\sqrt{\gamma_{S}^{LW}\gamma_{B}^{LW}}-\sqrt{\gamma_{S}^{p}\gamma_{B}^{D}}-\sqrt{\gamma_{S}^{D}\gamma_{B}^{p}}-\gamma_{L} \end{array}).$$ (5)

According to the thermodynamic theory, if *ΔF*_*Adh*_ is negative, bacteria spreading is energetically favorable; while if *ΔF*_*Adh*_ is positive, bacteria spreading is thermodynamically unfavorable.

### Bacteria tests

*S*. *aureus* (ATCC 25923), *P*. *aeruginosa* (ATCC 25668), and *Methicillin-resistant S*. *aureus (*MRSA*)* (ATCC 43300) were inoculated in 3% Tryptic soy broth (TSB) for 14 h. Afterwards, through primary incubation the bacterial cell concentration could be determined. This bacteria solution was diluted in order to produce working solution at concentrations of 10^4^ cells/mL. Afterwards, the samples (10 mm x 10 mm x 0.5 mm) in triplicate previously sterilized were set into 24-well tissue culture plates and individually inoculated with 1000 μL of bacteria working solution, and incubated for 24 h. Afterwards, the samples were lightly rinsed with PBS (x2) to remove any non-adherent bacteria, next the scaffolds were set into individual vials with PBS (1000 μL) then vortexed to remove the strongly adherent bacteria during 15 min. This bacterial solution suspensions were diluted serially (10X; 100X; 1000X) and plated on tryptic soy agar plates as 10 μL aliquots, in triplicate and incubated overnight (~14 h) at 37°C. Afterwards, the colonies were counted to calculate the colony-forming units (CFUs). The data were normalized by raw PCL data to highlight the bactericidal activity of the produced scaffolds without the use of antibiotics.

### Bacterial adhesion characterization by scanning electron microscopy

Scanning electron microscopy (SEM) was performed to assess bacterial colonization along fibrous scaffold surfaces. PCL, crosslinked GelMA, and crosslinked PCL:PEG:GelMA fibers were incubated for 24 h with 10^4^ cells/mL of biofilm-forming *S*. *aureus* inside a 24-well plate. Following the incubation period, the fibers were carefully transferred to a sterile 24-well plate and rinsed x2 lightly with PBS. The PBS was removed completely and replaced by 1 mL of a primary fixative solution containing 2.25% of glutaraldehyde in 0.1 M of cacodylate buffer. The fibers were maintained in the fixing solution for 4 days at 4°C before being dehydrated in a graded series of ethanol (30%, 50%, 70%, 80%, 90%, and 100%). The test samples were critical-point dried with carbon dioxide and sputter coated with a 5 nm thin layer of platinum before being imaged using a Hitachi S-4800 SEM.

### Protein adsorption evaluation

For protein adhesion test, the fibers were placed in a 24-well plate, 0.5 mL of TSB or casein solution were added and the plate was kept inside an incubator at 37°C for 24 h. After this time, the samples were transferred to a new plate, washed twice with PBS and 0.5 mL of immunoprecipitation assay (RIPA) buffer was added to each well. After 10 min in contact with RIPA buffer, the solution inside the wells was mixed with pipette to ensure homogeneity. Using a 96-well plate, 25 μL of the samples were mixed with 200 μL of working reagent from Pierce bicinchoninic acid (BCA) Protein Assay Kit, Thermo Scientific. The plate was placed on a plate shaker for 30 s and then incubated at 37°C for 30 min. After incubation, the plate was cooled to room temperature and the absorbance was measured at 562 nm using a spectrophotometer (SpectraMax M3, Molecular Devices).

### Analyses of the oxygen species

The reactive oxygen species (ROS) levels before and after UV crosslinking was analyzed using Fluorimetric Hydrogen Peroxide (H_2_O_2_) Assay Kit (Sigma-Aldrich) and the Cellular ROS/Superoxide (O_2_^∙-^) Detection Assay (Abcam). 1 mL of *S*. *aureus*, *P*. *aeruginosa* and MRSA solutions with concentration of 10^4^ CFU/mL were placed into 24-well plates containing the fibers. The plates were kept inside the incubator at 37°C for 24 h. After this time, the bacteria solution was removed, subsequently, the scaffolds were washed 2x with 1 mL PBS each, and then placed inside an eppendorf tube containing 1 mL of PBS. After vortex for 15 min, the solutions were used to perform the 2 tests.

For the hydrogen peroxide assay, 50 μL of each solution was placed in a 96-well plate and mixed with 50 μL of working solution from the assay kit. The 96-well plate was incubated at room temperature for 30 min followed by measuring the absorbance at 540 nm using a spectrophotometer (SpectraMax Paradigm, Molecular Devices).

In the more generalized ROS and superoxide detection process, a microplate assay procedure was implemented in which a mixture containing reactive cell permeable probes was added to the test samples. Specifically, 100 μL of each test and control was transferred from the eppendorf tubes after vortexing to an opaque 96-well plate. Positive controls were established in which a set of cells were treated with Pyocyanin in order to induce ROS generation. 100 μL of the ROS/superoxide detection mixture was added to the cell suspensions inside the 96-well plate, and the plate was allowed to incubate at 37°C for 1 h in the dark. After the staining period, the 96-well plate was loaded onto the spectrophotometer with standard fluorescein (Excitation/Emission = 490/525 nm) and rhodamine (Excitation/Emission = 550/620 nm) filter sets for detecting ROS and superoxide, respectively.

### Cell viability

The cell viability was determine using the MTS (CellTiter 96 AQueous One Solution Cell Proliferation Assay, G3581; Promega Corporation, Fitchburg, WI, USA) assay with hFOB bone forming cells, CRL-11372. The cells were seeded at 20.000 cells cm^2^ onto the sterilized sample in each well of a 24-tissue culture well-plate and were then incubated under standard cell culture conditions during 14 days. After the MTS reagent (1:5 ratio with cell culture media) was added to each well and incubated for 4 h at 37°C and 5% CO_2_ on the day of the measurement (14 days). Absorbance from each well was measured by a SpectraMax M3(MT05412) at 490 nm. Results were determinate by subtracting the values measured for the samples from the value of a comparable blank solution.

### In vivo biocompatibility analysis

All animal’s handling and surgical procedures were strictly conducted according to the Guiding Principles for the Use of Laboratory Animals. This study was approved by the Animal Care Committee guidelines of the São Carlos Federal University (protocol 8577280716). 10 male Wistar rats weighting 210–260 g and aged 8 weeks were used. During the experimental period, the animals were maintained under controlled conditions of light–dark periods of 12 h and temperature (24±2 ^o^C), with free access to water and commercial diet. Initially, the animals were anesthetized with Ketamine (80 mg/kg) and Xylazine (10 mg/kg). To insert the subcutaneous implants, rats were immobilized on their dorsal region, and the skin was shaved and disinfected with iodine. In each animal, four incisions of approximately 8 mm were made along the back, 2 being on the left and 2 on the right side, using sterile fields, followed by divulsion with straight surgical scissors. The implants were randomly placed and the skin was sutured with a 4–0 nylon monofilament (ShalonVR). The animals were housed in pairs. In the initial postoperative period, the intake of water and food was monitored. Furthermore, the animals were observed for signs of pain, infection, and proper activity. Animals were euthanized after 5 days with a lethal dose of anesthetic (Ketamine/Xylazine) and the biomaterials were harvested with surrounding tissue for histopathological analysis.

### Histopathological analysis

The specimens were fixed in 10% buffered formalin (Merck, Darmstadt, Germany) for 24 h, followed by dehydration in a graded series of ethanol and embedding in paraffin. In the transverse axis to the implant, thin sections (5 μm) were prepared using a microtome (Leica Microsystems SP 1600, Nussloch, Germany). The specimens were stained with hematoxylin and eosin (H.E. stain, Merck) and examined using optical microscopy (Olympus Optical Co., Tokyo, Japan).

### Statistical analysis

All experiments were conducted in triplicate and were repeated at least three different times with analysis of variance (ANOVA) followed by Student’s t-tests to determine statistical differences between mean values. The populations from the analyzed scaffolds were obtained with normal distribution and independent to each experiment.

## Results and discussions

**Fig 2:**
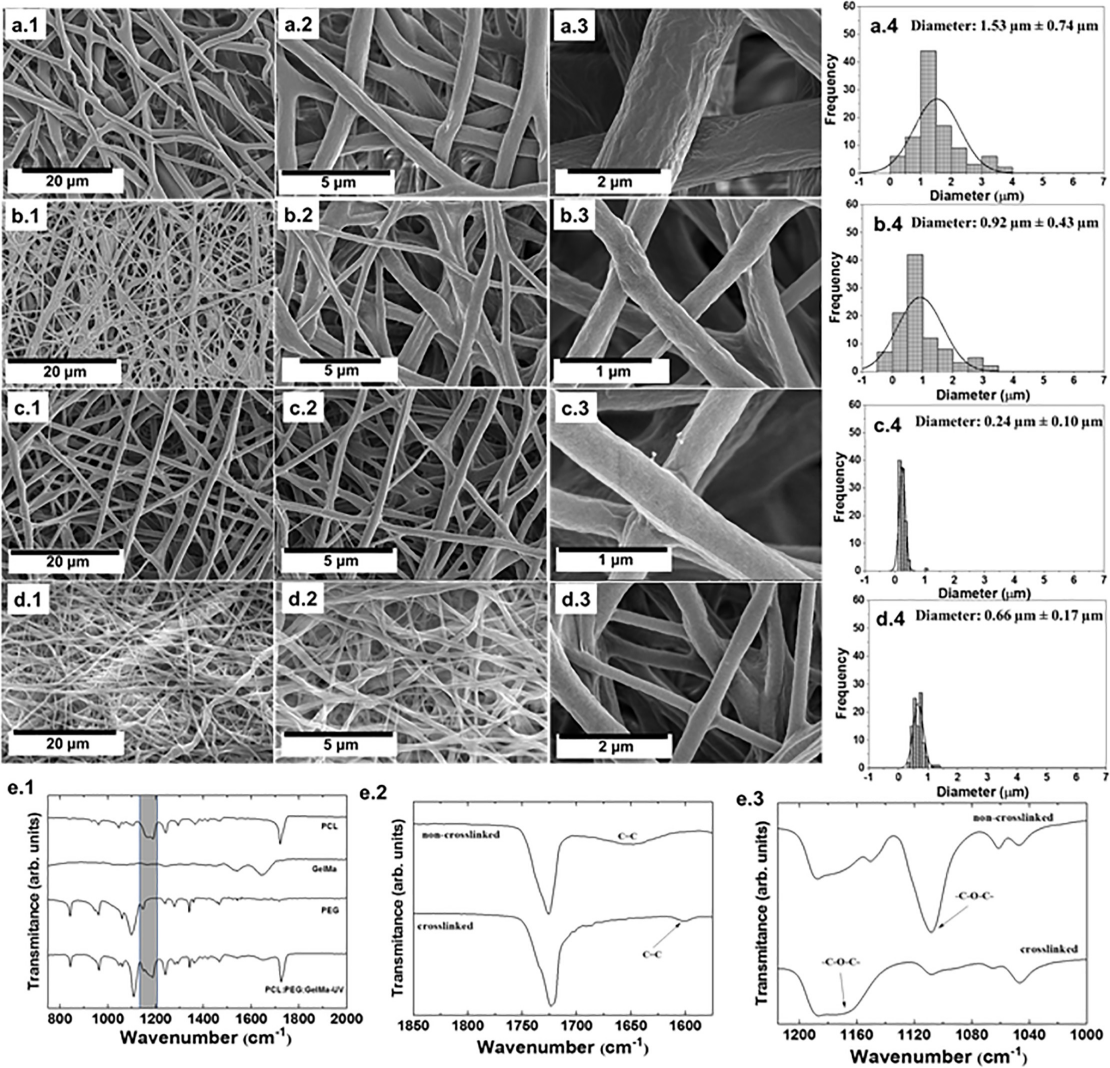
Characterization of the produced scaffolds. Scanning electron microscopy of: (**a1–a3**) PCL, (**b1–b3**) PCL:PEG, (**c1–c3**) PCL:PEG:GelMA and (**d1–d3**) crosslinked PCL:PEG:GelMA scaffolds. Distribution of diameters from (**a4**) PCL, (**b4**) PCL:PEG, (**c4**) PCL:PEG:GelMA and (**d4**) crosslinked PCL:PEG:GelMA scaffolds. (**e1–3**) Corresponding FTIR spectra of the produced scaffolds. (**e1**) Details of all electrospun scaffolds identifying the main peaks and bands to each polymer. (**e2**) The specific FTIR region identifying the vibrational region referred to the C = C bond from non- and crosslinked scaffolds. (**e3**) The main FTIR region containing the vibration of ether groups related to the methacryloyl substitution of GelMA. The groups were analyzed before and after UV irradiation.

The surface and structural characterization of the scaffolds are summarized in **Fig 2**. All the analyzed groups displayed ultrathin and microfibrous scaffolds in the absence of any beads independently of the analyzed groups. When the PCL solution was electrospun, a non-homogeneous behavior, and some defected fibers were produced (**Fig 2A1**). More details about the surface defects can be seen in **Fig 2A2** and **Fig 2A3**, which highlights the partial roughness structure and defects on the scaffold. The distribution of diameters is depicted in **Fig 2A4**, where PCL presented microfibrous structures (diameter of 1.53 μm ± 0.74 μm). After the incorporation of PEG, a discrete regularity in the material was obtained (**Fig 2B1**) and the PCL:PEG scaffolds presented a better smoothness (**Fig 2B2**). The smooth surface of the fibers could be more easily identified in the magnified **Fig 2B3** image. The thickness also decreased by 30% compared to PCL (**Fig 2B4**, 0.92 μm ± 0.43 μm). Furthermore, after the addition of GelMA the electrospun fibers transformed to an even more regular scaffold (**Fig 2C1**). **Fig 2C2** illustrates details from the non-defected structures with a homogeneous behavior. By comparing the detailed SEM image **Fig 2C3**, with PCL (**Fig 2A1–2A3**) and PCL:PEG (**Fig 2B1–2B3**) scaffolds, a smoother surface was obtained. Moreover, the distribution of the diameters from the PCL:PEG:GelMA scaffolds showed ultrathin fibers (0.24 μm ± 0.10 μm) smaller than PCL. The crosslinked PCL:PEG:GelMA scaffolds also presented a more homogeneous surface without any further defects (**Fig 2D1–2D3**), and with increases in diameter (**Fig 2D4**) after crosslinking compared to non-crosslinked PCL:PEG:GelMA (**Fig 2C1–2C3**).

The structural analysis and chemical interactions were recorded on the FTIR analysis (**Fig 2E1–2E3**). The main peaks for each separate polymer are shown in **Fig 2E1**. In the FTIR from the PCL, the main peaks could be identified at: 731 cm^-1^ (-(CH_2_)_*n*_), 1157 cm^-1^(C-O and C-C stretching in the amorphous phase), 1170 cm^-1^ (symmetric C-O-C stretching), 1190 cm^-1^ ((*ν*(O-C-O)), 1240 cm^-1^ (asymmetric C-O-C stretching), and 1342 cm^-1^ (CH_2_) [33, 34]. Also, the fingerprint region center was observed at: 1637 cm^−1^ (amide I), 1529 cm^−1^ (amide II), and 1448 cm^−1^ (amide III), corresponding to the stretching of the C = O bond, bending of the N-H bond, and plane vibration of C-N and N-H, respectively [35]. Meanwhile, the main peaks related to PEG are: 839 cm^−1^ (*δ*(C–H)), 951.26 cm^−1^ (CH_2_), 1115.19 cm^−1^(C-O), and 1350 cm^−1^ (*ν*(C=C)) [36, 37]. In the region 1100–1200 cm^-1^ of the mixed polymers, the intermolecular forces such as hydrogen bonds, ions, and charge transfers could be observed [38].

**Fig 2E2** and **2E3** show the difference between non- and crosslinked scaffolds. It is known that FTIR helps to analyze the fraction of unreacted C = C double bonds of methacrylate or methacrylamide groups [39]. The band centered at 1634 cm^−1^ indicating the double bond was absent after UV irradiation (**Fig 2E2**), whilst this was present on the scaffold before UV irradiation (identified at **Fig 2E2**). The increased chemical crosslinking resulted in an enhanced physical network [39, 40]. After UV irradiation, a peak centered at 1600 cm^−1^ appeared (acrylic double bonds, C = C) and at the same time, the peak at 1100 and 1170 cm^−1^ disappeared (**Fig 2E3**, related to the -C-O-C- vibration commonly found in scaffolds containing GelMA). This can be associated to the reaction of the methacryloyl moiety during crosslinking [38, 41].

**Fig 3:**
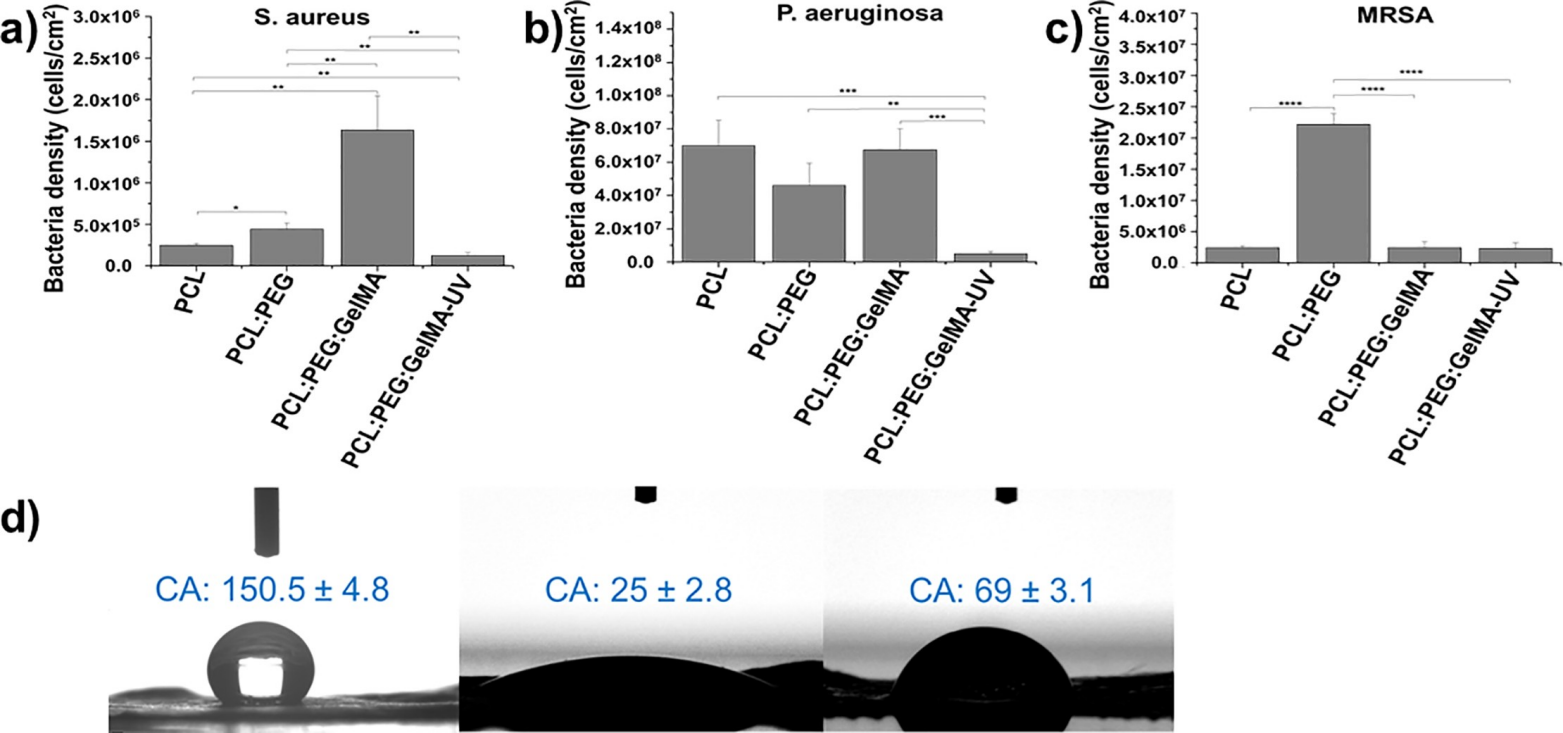
Bactericidal study on the electrospun scaffolds after 24 h (**a**) *S*. *aureus* growth reduction. (**b**) *P*. *aeruginosa* growth reduction. (**c**) MRSA growth reduction. The data were plotted in percentage and compared to raw PCL. N = 5, (**) p< 0.01, (***) p<0.001 and (****) p< 0.0001 mean statistical differences. (**d**) The wettability measured through contact angle analysis from water on PCL, PCL-PEG-GelMA and PCL-PEG-GelMA-UV scaffolds.

The bactericidal effect was evaluated against three most prominent bacteria in the hospital environment, such as: *S*. *aureus* (**Fig 3A**), *P*. *aeruginosa* (**Fig 3B**), and MRSA (**Fig 3C**). We compared these results to raw PCL. After UV crosslinking (PCL:PEG:GelMa-UV), the scaffolds inhibited the growth of *S*. *aureus* (**Fig 3A**, gram-positive) compared to raw PCL and with the others fibers. Similar results were observed in *P*. *aeruginosa* (**Fig 3B** gram-negative). However, the PCL:PEG:GelMA-UV scaffolds reduced both *S*. *aureus* and *P*. *aeruginosa* by 10-fold comparable to the others fibers, showing significantly with more than 90% bacteria reduction (1 log reduction) **(Fig 3A and 3B**). Moreover, our developed scaffolds showed high antibacterial activity against MRSA bacteria (**Fig 3C**) and the same efficiency was observed for the materials before and after UV crosslinking. We measured the contact angle (CA) of our developed scaffolds to explain this bactericidal activity presented for the PCL:PEG:GelMA before and after UV crosslinking and calculated their surface energy and adhesion force of bacteria attached using thermodynamic approaches (**Table 2**). Interesting, the PCL:PEG:GelMA-UV presented a CA (water, 69^o^) higher than the same material without UV crosslinking (water, 25^o^), probably due to a more dense material obtained after crosslinking introducing covalent bonds to the material. However, the CA using diiodomethane gave the same result for both materials with/without crosslinking (~5^o^, **Table 2**). We also calculated the surface energy of our developed scaffolds, and the material after UV crosslinking promoting the bactericidal activity showed a lower surface energy (53.3 mJ/m^2^) compared to the material without UV crosslinking (71.4 mJ/m^2^). Moreover, the adhesion force was calculated using a thermodynamic approach where the average value of work obtained for the adhesion of bacteria from PCL:PEG:GelMA were *deltaF*_*Adh*_ = -34.6 mJ/m^2^ (without crosslinking) and *deltaF*_*Adh*_ = -22.6 mJ/m^2^ (after crosslinking). These results indicated that the scaffold surfaces before UV crosslinking was more favorable for bacteria adhesion than after [31].

**Table 2:** Contact angle and surface energy components of PCL:PEG:GelMA and PCL:PEG:GelMA-UV scaffolds. Each mean value corresponds to the average value on three different samples.

Sample	Contact angle (°)		Surface free energy (mN m^-1^)			$\frac{\gamma_{p}}{\left(\gamma_{d}+\gamma_{p}\right)}$
	Water	Diiodomethane	Dispersive (*γ*_*d*_)	Polar (*γ*_*p*_)	Total	
PCL:PEG:GelMA	25 ± 2.8	5 ± 0.0	40.6	30.8	71.4	0.43
PCL:PEG:GelMA-UV	69 ± 3.1	5 ± 0.0	47.5	5.85	53.35	0.11

**Fig 4:**
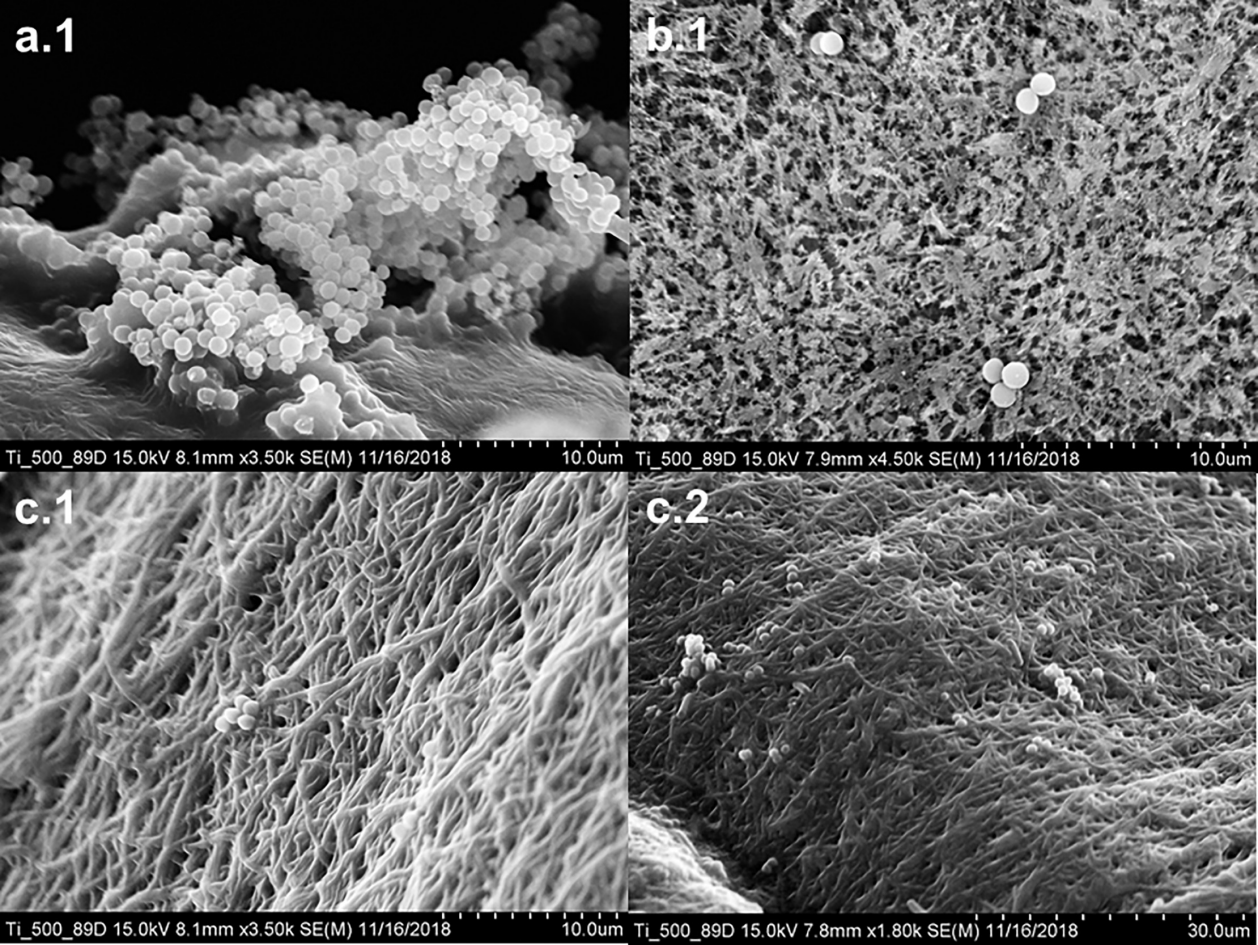
Scanning electron microscopy (SEM) images of PCL (**a1**), crosslinked GelMA (**b1**), and crosslinked PCL:PEG:GelMA (**c1–2**) fibers pre-treated with biofilm-forming *S*. *aureus*, taken at magnifications of x3.50k, x4.50k, x3.50k, and x1.80k, respectively, using an SEM operating at 15.0 kV.

SEM imaging was adopted in order to assess the tendency for biofilm-forming bacteria to adhere to crosslinked GelMA and PCL:PEG:GelMA fibers, relative to PCL control fibers. SEM representations of fiber surfaces pre-incubated with 10^4^ CFU/ml of *S*. *aureus* are shown in **Fig 4**. The PCL control surface (**Fig 4A1**) demonstrated a high density of bacterial colonization, whereas crosslinked GelMA (**Fig 4B1**) and crosslinked PCL:PEG:GelMA fibers (**Fig 4C1 and 4C2**) were generally less populated with bacteria. The images shown in **Fig 4A–4C1** were taken at similar intermediate magnifications ranging from x3.5k –x4.5k, and an additional representation (**Fig 4C2**) of the primary crosslinked PCL:PEG:GelMA fiber matt proposed in this study is provided at a lower magnification (x1.5k) in order to illustrate the low bacterial colonization over a wide surface area coverage. The data derived from this study suggests that crosslinking effectively reduces bacterial adhesion to fibrous surfaces, even for scaffolds exposed to biofilm-forming pathogenic strains.

**Fig 5:**
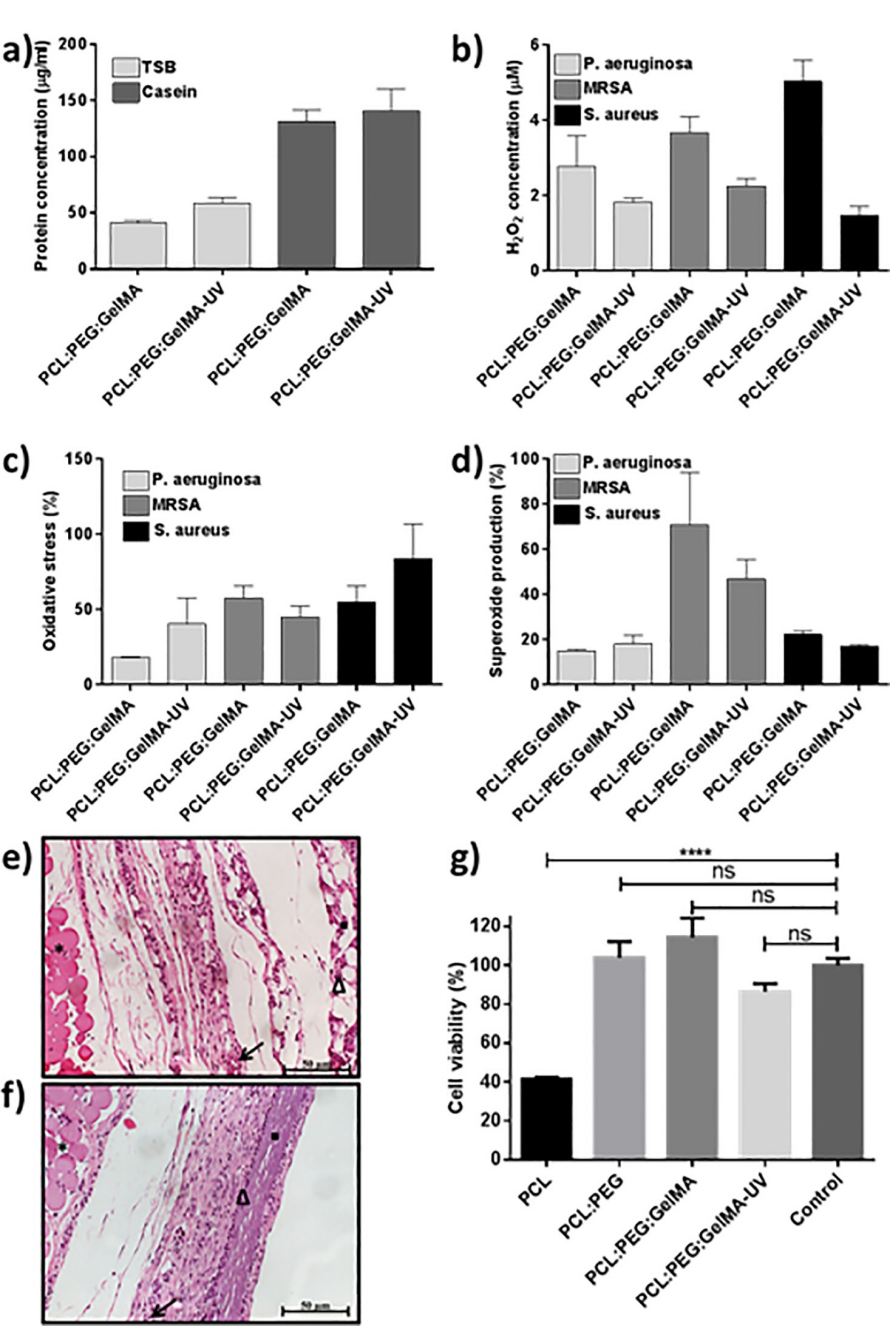
(**a**) Protein adsorption after 24 h. (**b**) Hydrogen peroxide assay, (**c**) reactive oxygen species (ROS) assay and (**d**) superoxide assay, after 24 h immersed in *S*. *aureus*, *P*. *aeruginosa* and MRSA bacteria solution, where percentages are given relative to positive control cells treated using a Pyocyanin ROS inducer. N = 3. Photomicrograph of subcutaneous implants in rats (**e**) PCL-PEG-GelMA and (**f**) PCL-PEG-GelMA-UV scaffolds. ▪ Biomaterials, Δ cell inflammatory, * panniculus carnosus muscle. Hematoxylin and eosin (H. E.) stain. Bar = 50 μm← blood vessel. (**g**) Cell viability results performed through MTS assay. The results were obtained on day 14.

In order to analyze the effect of the UV crosslinked fibers on the bactericidal properties, the non-crosslinked and crosslinked fibers were submitted to protein adsorption assay (**Fig 5A**), hydrogen peroxide assay (**Fig 5B**), reactive oxygen species (ROS) assay (**Fig 5C**) and superoxide assay (**Fig 5D**). The protein adsorption assay showed for both TSB and casein an increase of the protein concentration adsorbed onto the crosslinked fibers. Impressively, bacterial adhesion to the implant surfaces is inhibited, despite the generalized understanding that TSB and casein aid in bacterial growth ^[^^34^^]^. The reason for this is that casein adsorption to an implant surface prevents biomolecular attachment, and the increased protein adsorption over a restricted time period correlates with antibacterial properties due to short-scale bacterial adhesion dynamics [35]. From this study, it is possible to conclude that the UV crosslinking of the scaffold blend improves protein adsorption, and, correspondingly, reduces bacterial attachment and growth.

The UV crosslinking also influences the production of ROS, such as hydrogen peroxide (**Fig 5B**). The data obtained using the Fluorimetric Hydrogen Peroxide Assay Kit indicates that crosslinking of the fibers contributed to a decrease in the production of hydrogen peroxide for all three of the bacterial types evaluated. Nevertheless, the overall oxidative stress imposed on the *P*. *aeruginosa* and *S*. *aureus* strains due to the combined effect of hydrogen peroxide, hydroxyl radicals (HO∙), peroxynitrite (ONOO-), and peroxy radicals (ROO∙), as measured using the ROS/Superoxide Detection Assay, is augmented for the UV crosslinked samples (**Fig 5C**). This indicates that scaffold crosslinking plays a role in promoting the production of ROS that facilitate the self-destruction of *P*. *aeruginosa* and *S*. *aureus* cells. This conclusion, however, cannot be drawn for the drug-resistant MRSA strain. Indeed, a modest reduction in the ROS levels for MRSA suspensions that were grown along UV-crosslinked scaffolds was observed. Moreover, an increase in the overall oxidative stress imposed on the *P*. *aeruginosa* and *S*. *aureus* strains due to scaffold crosslinking did not correlate with an increase in superoxide production (**Fig 5D**), and the amount of O_2_^∙-^ generated by these bacteria upon exposure to both the UV-crosslinked and non-crosslinked samples was approximately the same. However, a significant decrease in the production of O_2_^∙-^ by MRSA cells incubated on the crosslinked scaffolds, relative to the non-crosslinked scaffolds was observed.

Additionally, the *in vivo* biocompatibility study performed on our scaffolds (PCL:PEG:GelMA) both prior and post UV crosslinking (**Fig 5E and 5F**) by subcutaneous implants in rats for five days implantation confirmed a normal appearance of the adjacent tissues and absence of necrosis. As depicted in **Fig 5E and 5F**, the capsules observed surrounding the implants does not demonstrate significant differences among the two scaffolds. Due to the empty space between the biomaterial and tissue, it could be assumed poor integration between the material and tissue. Furthermore, both scaffolds showed moderate inflammatory infiltrate surrounding and inside the implants, and moderate presence of tissue granulation and blood vessels surrounding the scaffolds. Overall, the *in vivo* study showed good compatibility of the scaffolds. Furthermore, the biocompatibility study was also expanded on testing the viability of mammalian cells seeded on these scaffolds (**Fig 5G**). The results displayed good biocompatibility except for the PCL fiber, this could probably be a result due to the hydrophobic nature of PCL, thus the cell adheres poorly.

Herein, we combined three different polymers to obtain ultrathin electrospun meshes. To date, we are not aware of any prior reports that have successfully demonstrated the electrospinning of the combination of PCL, PEG, and GelMA and evaluation on their bactericidal activity. Their efficiency was more evident after UV photocrosslinking, which could be associated to the closer interaction between the polar groups (such as -NH_2_, -OH, and the carbonyl group (-CO)) in GelMA and -OH found on the PEG chain, and also due to the more compact scaffold obtained. The surface energy changes of the scaffolds after UV crosslinking (**Table 2**) can lead to changes in protein adsorption and bioactivity altering cell functions and, at the same time, reducing bacteria adhesion and proliferation, since it is known that bacteria recognizes surface proteins to adhere [42, 43]. Furthermore, it is widely accepted that reactive chemical groups on micro and nano-fibers can disrupt bacteria membranes via oxygen free radical formation [44]. Therefore, it can be inferred, especially in the case of MRSA, that factors besides ROS and superoxide radical (O_2_^∙-^) production contribute to the bactericidal efficacy of PCL:PEG:GelMA scaffolds post crosslinking. This is beneficial considering that cellular mechanisms have evolved to evade killing by self-generated ROS (**Fig 5C**). For instance, adhesion prevention appears to factor significantly into disabling MRSA attachment and growth along scaffolds that incorporate GelMA. Although ROS are essential biological mediators of cell function, mainly at sites of inflammation some researchers showed the potential of this signal as an induce for material degradation, in which can provide scaffolds with better rates of tissue growth and biodegradation of the material [45–47].

Scaffolds based on different amounts of PCL, GelMA, and gelatin have been reported by Correa et al. [48]. However, the authors first produced PCL mats and then incorporated GelMA and gelatin afterwards using *in situ* coating. The process presented here is different and more interesting to tissue engineering applications since we electrospun PCL, PEG and GelMA together and the subsequent photocrosslinked blend promoted bactericidal activity. Furthermore, improved hydrophilic scaffold (CA ~25^o^) was obtained after GelMA and PEG inclusion. The potential of water absorption of GelMA and GelMA:PLGA mats were also investigated by Zhao et al. [49]. The authors obtained a highly porous scaffold which increased the diameter to ~70% after 24 h.

Other authors have explored the influence of GelMA and PEG to induce vascularization and extracellular matrix calcification [50–56], however, the bactericidal properties have not yet been investigated.

Annabi et al. reported that hydrogels based on GelMA conjugated with an amphiphilic peptide reduced bacteria growth [57]. However, the bactericidal effect against *S*. *aureus* was only observed after peptide incorporation. Commonly, micro- and nanoparticles have been associated to short sequences of cationic amino acids to possess a broad-spectrum of bactericidal activity against Gram (+/−) bacteria [58–61]. Here, impressively, the PCL:PEG:GelMa-UV reduced both *S*. *aureus* and *P*. *aeruginosa* by 10-fold, corresponding to more than 90% bacteria reduction compared to raw PCL without requiring additional steps of peptide functionalization or antibiotic incorporation, such data considered clinically relevant [62]. Additionally, no statistical difference was observed compared to PCL:PEG:GelMA before and after UV against MRSA.

Rujitanaroj et al. combined gelatin and GelMA with silver nanoparticles and evaluated their bactericidal effect [63]; the use of silver has recently been questioned due to high mammalian cell toxicity. Moreover, the authors only observed the bactericidal effect when silver nanoparticles were added. In this manner, it is plausible to at least preliminarily compare the antimicrobial mechanism of our scaffold to those obtained when amphiphilic peptides (AMPs) were used. It is noteworthy that our developed scaffold had no positive charge in its structure, indicating that the binding interactions between the hydrogel and gram-positive and negative bacteria were unlikely to be caused by electrostatic interactions, as seen in the AMPs, which involved bacterial cell wall and membrane disruption [64–66]. We believe that the design of a more hydrophilic scaffold promoted the promising results obtained from the bacteria study by altering initial protein adsorption that bacteria depend upon to attach. All these characteristics highlight the application of PCL:PEG:GelMA as novel scaffolds for tissue engineering purposes. Further studies will be needed to determine the exact mechanisms (including initial ion/protein adsorption) responsible for the reduced bacteria growth on the present scaffolds.

In this report we disclose, for the first time, the preparation and bactericidal proficiency investigation of electrospun scaffolds by merging PCL:PEG:GelMA polymers. The designed ultrathin fibers scaffolds were hydrophilic comparable to raw PCL. Moreover, the developed scaffolds showed a reduced susceptibility the growth of *S*. *aureus*, *P*. *aeruginosa*, and MRSA after UV crosslinking. Moreover, the *in vivo* subcutaneous implantation performed in rats together with the viability test of mammalian cells seeded on these scaffolds confirmed the biocompatibility of our designed scaffolds. In conclusion, all these findings suggest that the designed biomaterial is a promising candidate for various biomedical applications.

## Supporting information

S1 Table FTIR data. The raw data for the FTIR presented on Fig 2 of samples PCL, GelMA, PEG and PCL-PEG-GelMA-UV.
(PDF)

S2 Table FTIR data. The raw data for the bacteria test presented in Fig 3.
(PDF)

S3 Table FTIR data. The raw data for the Hydrogen peroxide assay, BCA protein assay, Reactive oxygen species (ROS) assay, Superoxide assay and Cell viability test presented in Fig 5.
(PDF)
