# Supplementary material for: Understanding the impact of crosslinked PCL/PEG/GelMA electrospun nanofibers on bactericidal activity
Source: PLoS One. 2018 Dec 20;13(12):e0209386. doi: 10.1371/journal.pone.0209386 (PMC6301679; doi:10.1371/journal.pone.0209386)
Supplement: S1 Table — The raw data for the FTIR presented on Fig 2 of samples PCL, GelMA, PEG and PCL-PEG-GelMA-UV. (PDF) [file pone.0209386.s001.pdf]

FTIR data

	PCL		GelMA		PEG		PCL-PEG-GelMA-UV
4000	167.375	4000	143.1899	4000	120.9309	4000	88.11274
3999	167.3716	3999	143.1809	3999	120.9319	3999	88.10917
3998	167.3616	3998	143.168	3998	120.9353	3998	88.10083
3997	167.3491	3997	143.1946	3997	120.9362	3997	88.09003
3996	167.3398	3996	143.1923	3996	120.9337	3996	88.08182
3995	167.3367	3995	143.1889	3995	120.927	3995	88.12513
3994	167.3771	3994	143.1866	3994	120.9176	3994	88.12734
3993	167.3808	3993	143.1879	3993	120.9084	3993	88.13128
3992	167.386	3992	143.1933	3992	120.903	3992	88.13615
3991	167.392	3991	143.2007	3991	120.9044	3991	88.1414
3990	167.3979	3990	143.2079	3990	120.9124	3990	88.14634
3989	167.4026	3989	143.214	3989	120.9238	3989	88.15041
3988	167.4055	3988	143.2188	3988	120.9346	3988	88.15292
3987	167.4062	3987	143.2218	3987	120.9418	3987	88.15331
3986	167.4045	3986	143.2219	3986	120.9446	3986	88.15168
3985	167.4002	3985	143.2192	3985	120.9435	3985	88.14901
3984	167.3933	3984	143.2157	3984	120.9402	3984	88.14633
3983	167.3852	3983	143.2137	3983	120.9363	3983	88.14401
3982	167.3778	3982	143.2139	3982	120.9319	3982	88.14229
3981	167.3735	3981	143.2154	3981	120.9254	3981	88.14225
3980	167.3728	3980	143.2164	3980	120.9149	3980	88.14475
3979	167.3743	3979	143.2156	3979	120.9	3979	88.14833
3978	167.3759	3978	143.2127	3978	120.8833	3978	88.14961
3977	167.3763	3977	143.2085	3977	120.8702	3977	88.14645
3976	167.3758	3976	143.2048	3976	120.8653	3976	88.14056
3975	167.3742	3975	143.2026	3975	120.869	3975	88.13624
3974	167.371	3974	143.2017	3974	120.8776	3974	88.13634
3973	167.3665	3973	143.2014	3973	120.8872	3973	88.14
3972	167.3629	3972	143.2014	3972	120.8963	3972	88.14368
3971	167.3628	3971	143.2019	3971	120.906	3971	88.14401
3970	167.3663	3970	143.2025	3970	120.9167	3970	88.1398
3969	167.3711	3969	143.2019	3969	120.9263	3969	88.13278
3968	167.374	3968	143.1997	3968	120.9318	3968	88.12674
3967	167.3737	3967	143.1972	3967	120.9309	3967	88.12521
3966	167.3715	3966	143.1966	3966	120.9241	3966	88.12957
3965	167.3702	3965	143.1992	3965	120.9141	3965	88.13846
3964	167.3716	3964	143.2036	3964	120.9046	3964	88.14837
3963	167.3752	3963	143.2065	3963	120.8978	3963	88.15546
3962	167.3788	3962	143.2055	3962	120.8937	3962	88.15792
3961	167.3808	3961	143.2013	3961	120.8916	3961	88.15692
3960	167.3807	3960	143.1962	3960	120.8907	3960	88.15481
3959	167.379	3959	143.1916	3959	120.8917	3959	88.15306

3958	167.3765	3958	143.1876	3958	120.895	3958	88.15129
3957	167.3739	3957	143.1848	3957	120.8994	3957	88.14882
3956	167.3726	3956	143.1854	3956	120.9036	3956	88.14643
3955	167.373	3955	143.1911	3955	120.9081	3955	88.1458
3954	167.3748	3954	143.2016	3954	120.9131	3954	88.14791
3953	167.3767	3953	143.2135	3953	120.9168	3953	88.15186
3952	167.3778	3952	143.2219	3952	120.9147	3952	88.15501
3951	167.3774	3951	143.2219	3951	120.9036	3951	88.15437
3950	167.3746	3950	143.2113	3950	120.8823	3950	88.14843
3949	167.3701	3949	143.1937	3949	120.8534	3949	88.13928
3948	167.3661	3948	143.1787	3948	120.8257	3948	88.13246
3947	167.3673	3947	143.178	3947	120.8144	3947	88.13422
3946	167.3777	3946	143.1985	3946	120.8339	3946	88.14796
3945	167.396	3945	143.2343	3945	120.8867	3945	88.17084
3944	167.4141	3944	143.2671	3944	120.9547	3944	88.19322
3943	167.4226	3943	143.2786	3943	121.0028	3943	88.20402
3942	167.4182	3942	143.2667	3942	121.0042	3942	88.19957
3941	167.4058	3941	143.2454	3941	120.9666	3941	88.18646
3940	167.3931	3940	143.2287	3940	120.9213	3940	88.17439
3939	167.3843	3939	143.2199	3939	120.8935	3939	88.16898
3938	167.3798	3938	143.2139	3938	120.8872	3938	88.16985
3937	167.3789	3937	143.2072	3937	120.8893	3937	88.17375
3936	167.3807	3936	143.2012	3936	120.8852	3936	88.17789
3935	167.3827	3935	143.198	3935	120.8691	3935	88.17998
3934	167.3818	3934	143.1967	3934	120.8456	3934	88.17821
3933	167.3782	3933	143.1958	3933	120.8265	3933	88.17297
3932	167.377	3932	143.1974	3932	120.8236	3932	88.16842
3931	167.3821	3931	143.2035	3931	120.8387	3931	88.16846
3930	167.3914	3930	143.2119	3930	120.8605	3930	88.17236
3929	167.4024	3929	143.2227	3929	120.8813	3929	88.17915
3928	167.4147	3928	143.2391	3928	120.9051	3928	88.19002
3927	167.4255	3927	143.2593	3927	120.9344	3927	88.20303
3926	167.4282	3926	143.2714	3926	120.959	3926	88.21041
3925	167.4207	3925	143.2657	3925	120.9624	3925	88.20638
3924	167.4106	3924	143.2489	3924	120.9386	3924	88.19532
3923	167.4058	3923	143.2334	3923	120.8979	3923	88.1852
3922	167.4042	3922	143.2212	3922	120.8554	3922	88.178
3921	167.3982	3921	143.2054	3921	120.8188	3921	88.17029
3920	167.3856	3920	143.1839	3920	120.7894	3920	88.16066
3919	167.3743	3919	143.1676	3919	120.7725	3919	88.15374
3918	167.3752	3918	143.1693	3918	120.7814	3918	88.15584
3917	167.3893	3917	143.1892	3917	120.8217	3917	88.16704
3916	167.408	3916	143.2153	3916	120.8775	3916	88.1812
3915	167.422	3915	143.2366	3915	120.9252	3915	88.19305
3914	167.4268	3914	143.2476	3914	120.9534	3914	88.20043

3913	167.422	3913	143.2469	3913	120.9647	3913	88.20271
3912	167.4104	3912	143.2376	3912	120.966	3912	88.20061
3911	167.3977	3911	143.2275	3911	120.9645	3911	88.19762
3910	167.3888	3910	143.2224	3910	120.9645	3910	88.19746
3909	167.3852	3909	143.2218	3909	120.963	3909	88.20017
3908	167.3846	3908	143.2186	3908	120.9448	3908	88.20044
3907	167.3813	3907	143.2033	3907	120.8881	3907	88.18997
3906	167.369	3906	143.1695	3906	120.782	3906	88.16264
3905	167.347	3905	143.121	3905	120.6433	3905	88.12219
3904	167.327	3904	143.0785	3904	120.5129	3904	88.0863
3903	167.3263	3903	143.0668	3903	120.4326	3903	88.0755
3902	167.3504	3902	143.0916	3902	120.423	3902	88.09482
3901	167.3876	3901	143.1356	3901	120.4788	3901	88.13171
3900	167.4181	3900	143.1745	3900	120.5725	3900	88.16751
3899	167.43	3899	143.1964	3899	120.663	3899	88.18992
3898	167.4265	3898	143.205	3898	120.7216	3898	88.19772
3897	167.4154	3897	143.2055	3897	120.7452	3897	88.19442
3896	167.4011	3896	143.1966	3896	120.7407	3896	88.18206
3895	167.3861	3895	143.1796	3895	120.7194	3895	88.1645
3894	167.3731	3894	143.1621	3894	120.697	3894	88.14796
3893	167.3637	3893	143.1512	3893	120.6907	3893	88.13768
3892	167.3559	3892	143.1428	3892	120.7014	3892	88.13319
3891	167.3481	3891	143.129	3891	120.7117	3891	88.13137
3890	167.3444	3890	143.1152	3890	120.7124	3890	88.13233
3889	167.3516	3889	143.1163	3889	120.7053	3889	88.13939
3888	167.3692	3888	143.1373	3888	120.6953	3888	88.154
3887	167.3861	3887	143.1639	3887	120.7009	3887	88.17094
3886	167.392	3886	143.1767	3886	120.7323	3886	88.18017
3885	167.3931	3885	143.182	3885	120.7771	3885	88.18353
3884	167.4036	3884	143.203	3884	120.8226	3884	88.19445
3883	167.4284	3883	143.2471	3883	120.875	3883	88.22105
3882	167.4591	3882	143.2932	3882	120.9464	3882	88.25683
3881	167.4806	3881	143.3089	3881	121.013	3881	88.28214
3880	167.4829	3880	143.2842	3880	121.0208	3880	88.28262
3879	167.4715	3879	143.2466	3879	120.9695	3879	88.26797
3878	167.4611	3878	143.231	3878	120.9144	3878	88.25897
3877	167.4591	3877	143.2465	3877	120.896	3877	88.26635
3876	167.4607	3876	143.2743	3876	120.9241	3876	88.2848
3875	167.4538	3875	143.2808	3875	120.9716	3875	88.29416
3874	167.4336	3874	143.2521	3874	120.9781	3874	88.27764
3873	167.4093	3873	143.21	3873	120.9073	3873	88.24098
3872	167.3908	3872	143.1815	3872	120.7917	3872	88.20276
3871	167.3771	3871	143.1643	3871	120.7007	3871	88.17274
3870	167.3639	3870	143.1407	3870	120.658	3870	88.14853
3869	167.3563	3869	143.1152	3869	120.624	3869	88.13054

3868	167.3624	3868	143.1101	3868	120.5706	3868	88.12625
3867	167.3786	3867	143.1273	3867	120.5092	3867	88.13788
3866	167.39	3866	143.1427	3866	120.4782	3866	88.1572
3865	167.3898	3865	143.1404	3865	120.5088	3865	88.17221
3864	167.3872	3864	143.1339	3864	120.5836	3864	88.17706
3863	167.3879	3863	143.1371	3863	120.6425	3863	88.17159
3862	167.3868	3862	143.1434	3862	120.6525	3862	88.15948
3861	167.3852	3861	143.1468	3861	120.6412	3861	88.15144
3860	167.3891	3860	143.149	3860	120.6512	3860	88.15598
3859	167.3986	3859	143.1499	3859	120.6935	3859	88.16911
3858	167.4052	3858	143.1442	3858	120.7355	3858	88.17621
3857	167.4015	3857	143.1314	3857	120.7266	3857	88.16703
3856	167.3893	3856	143.1248	3856	120.6413	3856	88.15043
3855	167.3722	3855	143.1338	3855	120.5415	3855	88.14687
3854	167.3497	3854	143.1353	3854	120.5026	3854	88.15273
3853	167.3524	3853	143.1415	3853	120.5451	3853	88.17556
3852	167.3864	3852	143.1711	3852	120.6275	3852	88.21402
3851	167.4205	3851	143.2039	3851	120.693	3851	88.24046
3850	167.4338	3850	143.2164	3850	120.7103	3850	88.24057
3849	167.4263	3849	143.2078	3849	120.6987	3849	88.224
3848	167.4102	3848	143.1935	3848	120.687	3848	88.20775
3847	167.3998	3847	143.1924	3847	120.698	3847	88.20615
3846	167.4016	3846	143.2099	3846	120.7359	3846	88.22196
3845	167.4122	3845	143.2348	3845	120.7833	3845	88.24523
3844	167.4241	3844	143.2491	3844	120.8125	3844	88.26183
3843	167.4332	3843	143.2472	3843	120.8113	3843	88.26629
3842	167.4389	3842	143.2375	3842	120.786	3842	88.26255
3841	167.4408	3841	143.2287	3841	120.7485	3841	88.25477
3840	167.4383	3840	143.2199	3840	120.7133	3840	88.2437
3839	167.4362	3839	143.2106	3839	120.6969	3839	88.23308
3838	167.4375	3838	143.2023	3838	120.6961	3838	88.22663
3837	167.4354	3837	143.1907	3837	120.6856	3837	88.2199
3836	167.4276	3836	143.1778	3836	120.6606	3836	88.21077
3835	167.4193	3835	143.1723	3835	120.6412	3835	88.20433
3834	167.4142	3834	143.1761	3834	120.6405	3834	88.20476
3833	167.4101	3833	143.1798	3833	120.6529	3833	88.21022
3832	167.4034	3832	143.1746	3832	120.6664	3832	88.2158
3831	167.3957	3831	143.1661	3831	120.6771	3831	88.22021
3830	167.3916	3830	143.1686	3830	120.691	3830	88.22566
3829	167.3926	3829	143.1881	3829	120.7108	3829	88.2322
3828	167.3942	3828	143.2115	3828	120.7301	3828	88.23364
3827	167.3907	3827	143.216	3827	120.7359	3827	88.22181
3826	167.3818	3826	143.1933	3826	120.7126	3826	88.19573
3825	167.3752	3825	143.1614	3825	120.6518	3825	88.16605
3824	167.3794	3824	143.1472	3824	120.5677	3824	88.15041

3823	167.3925	3823	143.1579	3823	120.5118	3823	88.15939
3822	167.398	3822	143.1634	3822	120.5226	3822	88.17625
3821	167.3925	3821	143.1453	3821	120.5648	3821	88.18128
3820	167.3915	3820	143.1318	3820	120.5866	3820	88.18335
3819	167.4044	3819	143.1516	3819	120.598	3819	88.19779
3818	167.4252	3818	143.1987	3818	120.6348	3818	88.22593
3817	167.4407	3817	143.2409	3817	120.7229	3817	88.2569
3816	167.4426	3816	143.2526	3816	120.833	3816	88.2747
3815	167.4355	3815	143.2428	3815	120.8969	3815	88.27503
3814	167.4287	3814	143.2369	3814	120.8944	3814	88.26728
3813	167.4215	3813	143.2381	3813	120.8572	3813	88.25769
3812	167.4093	3812	143.2342	3812	120.8227	3812	88.24681
3811	167.3964	3811	143.2248	3811	120.8082	3811	88.23752
3810	167.3943	3810	143.225	3810	120.8064	3810	88.2376
3809	167.4067	3809	143.241	3809	120.8038	3809	88.25123
3808	167.4211	3808	143.2515	3808	120.7946	3808	88.26771
3807	167.4248	3807	143.2366	3807	120.7802	3807	88.27222
3806	167.4237	3806	143.2169	3806	120.7697	3806	88.26738
3805	167.4319	3805	143.2287	3805	120.7708	3805	88.2675
3804	167.4503	3804	143.2763	3804	120.7822	3804	88.27821
3803	167.4651	3803	143.3199	3803	120.81	3803	88.28922
3802	167.4646	3802	143.311	3802	120.8467	3802	88.28382
3801	167.4544	3801	143.2577	3801	120.8509	3801	88.26389
3800	167.4449	3800	143.2122	3800	120.804	3800	88.24606
3799	167.4409	3799	143.2067	3799	120.7507	3799	88.24221
3798	167.4387	3798	143.2312	3798	120.7384	3798	88.2506
3797	167.4325	3797	143.256	3797	120.7766	3797	88.26063
3796	167.4225	3796	143.2635	3796	120.8296	3796	88.26372
3795	167.414	3795	143.261	3795	120.8655	3795	88.26246
3794	167.4106	3794	143.2623	3794	120.8871	3794	88.26407
3793	167.4093	3793	143.2687	3793	120.9083	3793	88.26988
3792	167.4073	3792	143.2751	3792	120.9327	3792	88.27675
3791	167.4052	3791	143.2799	3791	120.9518	3791	88.28183
3790	167.4046	3790	143.2836	3790	120.9546	3790	88.28398
3789	167.4061	3789	143.2865	3789	120.9417	3789	88.28434
3788	167.4096	3788	143.2887	3788	120.9264	3788	88.28527
3787	167.4145	3787	143.2904	3787	120.9222	3787	88.28799
3786	167.4191	3786	143.2899	3786	120.9326	3786	88.29128
3785	167.4221	3785	143.285	3785	120.9473	3785	88.29267
3784	167.4251	3784	143.2779	3784	120.9531	3784	88.29195
3783	167.4335	3783	143.2787	3783	120.9524	3783	88.29418
3782	167.4488	3782	143.2946	3782	120.9629	3782	88.30459
3781	167.4622	3781	143.3169	3781	120.9946	3781	88.3192
3780	167.4623	3780	143.3258	3780	121.0294	3780	88.32543
3779	167.4496	3779	143.315	3779	121.0347	3779	88.31769

3778	167.4358	3778	143.2977	3778	121.0041	3778	88.30407
3777	167.4288	3777	143.2874	3777	120.962	3777	88.29445
3776	167.4265	3776	143.2825	3776	120.9313	3776	88.29016
3775	167.4222	3775	143.2721	3775	120.9155	3775	88.28546
3774	167.4137	3774	143.2534	3774	120.9023	3774	88.27554
3773	167.4042	3773	143.2348	3773	120.8762	3773	88.26117
3772	167.3955	3772	143.2231	3772	120.835	3772	88.24654
3771	167.388	3771	143.2167	3771	120.7947	3771	88.23628
3770	167.3855	3770	143.214	3770	120.768	3770	88.23438
3769	167.3937	3769	143.2208	3769	120.7534	3769	88.2426
3768	167.4108	3768	143.2393	3768	120.7478	3768	88.25719
3767	167.4259	3767	143.2593	3767	120.7508	3767	88.27077
3766	167.4295	3766	143.2657	3766	120.7609	3766	88.27859
3765	167.4225	3765	143.2555	3765	120.7729	3765	88.28164
3764	167.4159	3764	143.2423	3764	120.7796	3764	88.28369
3763	167.4194	3763	143.2415	3763	120.7801	3763	88.28818
3762	167.4331	3762	143.2533	3762	120.7851	3762	88.29625
3761	167.4496	3761	143.265	3761	120.8062	3761	88.30616
3760	167.4623	3760	143.2672	3760	120.8322	3760	88.31457
3759	167.4691	3759	143.2621	3759	120.8347	3759	88.31832
3758	167.465	3758	143.2507	3758	120.8018	3758	88.31194
3757	167.4482	3757	143.2317	3757	120.7492	3757	88.29235
3756	167.427	3756	143.2126	3756	120.6939	3756	88.26583
3755	167.4105	3755	143.2024	3755	120.6357	3755	88.24215
3754	167.3941	3754	143.1931	3754	120.554	3754	88.21985
3753	167.3567	3753	143.1556	3753	120.4078	3753	88.17781
3752	167.2844	3752	143.0708	3752	120.1684	3752	88.09536
3751	167.2086	3751	142.9771	3751	119.9087	3751	88.00207
3750	167.1713	3750	142.9248	3750	119.7595	3750	87.95113
3749	167.1741	3749	142.912	3749	119.7535	3749	87.94533
3748	167.2074	3748	142.9289	3748	119.835	3748	87.96348
3747	167.2625	3747	142.9755	3747	119.9353	3747	87.99559
3746	167.317	3746	143.0346	3746	120.0278	3746	88.03846
3745	167.3379	3745	143.0632	3745	120.1086	3745	88.07755
3744	167.3328	3744	143.0624	3744	120.1845	3744	88.11054
3743	167.3435	3743	143.0846	3743	120.2828	3743	88.15358
3742	167.3791	3742	143.1412	3742	120.3934	3742	88.2035
3741	167.4185	3741	143.2002	3741	120.4837	3741	88.24443
3740	167.4464	3740	143.2395	3740	120.5482	3740	88.27167
3739	167.4633	3739	143.2632	3739	120.5876	3739	88.29207
3738	167.472	3738	143.2811	3738	120.6028	3738	88.31214
3737	167.4669	3737	143.2823	3737	120.5987	3737	88.32356
3736	167.4562	3736	143.2633	3736	120.5903	3736	88.31779
3735	167.4652	3735	143.2548	3735	120.5942	3735	88.3067
3734	167.485	3734	143.2649	3734	120.5865	3734	88.295

3733	167.4777	3733	143.2579	3733	120.5335	3733	88.27065
3732	167.4327	3732	143.2138	3732	120.4545	3732	88.23407
3731	167.3692	3731	143.1471	3731	120.3805	3731	88.1953
3730	167.3045	3730	143.0792	3730	120.2994	3730	88.15607
3729	167.2459	3729	143.0224	3729	120.1919	3729	88.11509
3728	167.2018	3728	142.9845	3728	120.0871	3728	88.0801
3727	167.1861	3727	142.9739	3727	120.0481	3727	88.06593
3726	167.2089	3726	142.9973	3726	120.1081	3726	88.08251
3725	167.262	3725	143.0486	3725	120.2383	3725	88.12393
3724	167.3242	3724	143.1097	3724	120.3914	3724	88.17406
3723	167.3791	3723	143.1659	3723	120.5412	3723	88.22019
3722	167.4219	3722	143.214	3722	120.6765	3722	88.25794
3721	167.4529	3721	143.2561	3721	120.7895	3721	88.28851
3720	167.4696	3720	143.2892	3720	120.8685	3720	88.31142
3719	167.4661	3719	143.302	3719	120.8969	3719	88.32033
3718	167.439	3718	143.2843	3718	120.8672	3718	88.30772
3717	167.3982	3717	143.2452	3717	120.7992	3717	88.27768
3716	167.3664	3716	143.2138	3716	120.733	3716	88.25025
3715	167.3583	3715	143.2114	3715	120.6959	3715	88.24432
3714	167.3602	3714	143.2212	3714	120.6668	3714	88.25253
3713	167.3354	3713	143.1937	3713	120.5791	3713	88.23617
3712	167.2769	3712	143.1138	3712	120.4136	3712	88.17502
3711	167.2388	3711	143.049	3711	120.2829	3711	88.11824
3710	167.257	3710	143.0517	3710	120.2766	3710	88.11493
3709	167.3085	3709	143.1017	3709	120.3563	3709	88.15545
3708	167.3605	3708	143.1617	3708	120.4635	3708	88.20917
3707	167.3998	3707	143.2118	3707	120.573	3707	88.25663
3706	167.4317	3706	143.2536	3706	120.6816	3706	88.2961
3705	167.4704	3705	143.3019	3705	120.8066	3705	88.33972
3704	167.523	3704	143.3659	3704	120.9675	3704	88.3981
3703	167.5764	3703	143.4286	3703	121.1429	3703	88.46008
3702	167.6046	3702	143.4546	3702	121.253	3702	88.49507
3701	167.5951	3701	143.4291	3701	121.235	3701	88.48578
3700	167.5605	3700	143.3761	3700	121.1179	3700	88.44657
3699	167.5207	3699	143.3288	3699	120.9803	3699	88.40284
3698	167.4838	3698	143.2973	3698	120.8748	3698	88.36737
3697	167.4472	3697	143.2701	3697	120.8073	3697	88.33763
3696	167.4095	3696	143.2357	3696	120.75	3696	88.30646
3695	167.3758	3695	143.1962	3695	120.6767	3695	88.27254
3694	167.3551	3694	143.166	3694	120.5988	3694	88.24514
3693	167.3582	3693	143.1659	3693	120.5653	3693	88.24099
3692	167.3926	3692	143.2114	3692	120.6218	3692	88.27248
3691	167.4577	3691	143.2992	3691	120.7711	3691	88.33671
3690	167.5351	3690	143.3942	3690	120.9564	3690	88.40939
3689	167.5853	3689	143.4412	3689	121.067	3689	88.45224

3688	167.5835	3688	143.4214	3688	121.0484	3688	88.44868
3687	167.5487	3687	143.3738	3687	120.9652	3687	88.42023
3686	167.5087	3686	143.3361	3686	120.883	3686	88.39008
3685	167.4719	3685	143.3108	3685	120.8179	3685	88.36235
3684	167.4369	3684	143.2847	3684	120.7642	3684	88.33256
3683	167.4077	3683	143.2565	3683	120.7201	3683	88.30232
3682	167.3913	3682	143.2366	3682	120.6912	3682	88.27953
3681	167.385	3681	143.2262	3681	120.682	3681	88.26623
3680	167.3779	3680	143.216	3680	120.6825	3680	88.25598
3679	167.3683	3679	143.2098	3679	120.677	3679	88.24837
3678	167.3709	3678	143.2274	3678	120.6727	3678	88.25754
3677	167.4027	3677	143.2733	3677	120.7263	3677	88.29717
3676	167.4564	3676	143.3078	3676	120.8402	3676	88.3456
3675	167.4987	3675	143.2982	3675	120.8873	3675	88.36419
3674	167.507	3674	143.2617	3674	120.8032	3674	88.34461
3673	167.4946	3673	143.2355	3673	120.6635	3673	88.31018
3672	167.4777	3672	143.2263	3672	120.5484	3672	88.2809
3671	167.465	3671	143.2235	3671	120.5352	3671	88.26957
3670	167.458	3670	143.2218	3670	120.6244	3670	88.27626
3669	167.4475	3669	143.2227	3669	120.7021	3669	88.2874
3668	167.4287	3668	143.2294	3668	120.7153	3668	88.29422
3667	167.4103	3667	143.2442	3667	120.7133	3667	88.29962
3666	167.4004	3666	143.2608	3666	120.7307	3666	88.30556
3665	167.4003	3665	143.2724	3665	120.767	3665	88.31175
3664	167.4056	3664	143.2768	3664	120.8062	3664	88.31719
3663	167.409	3663	143.2747	3663	120.836	3663	88.32032
3662	167.4042	3662	143.2654	3662	120.8504	3662	88.31735
3661	167.3892	3661	143.2478	3661	120.8445	3661	88.30393
3660	167.3683	3660	143.2264	3660	120.811	3660	88.28032
3659	167.3473	3659	143.2091	3659	120.7484	3659	88.25363
3658	167.3305	3658	143.2003	3658	120.6752	3658	88.23391
3657	167.3195	3657	143.1942	3657	120.6224	3657	88.22636
3656	167.3182	3656	143.1863	3656	120.6076	3656	88.23068
3655	167.3306	3655	143.1828	3655	120.623	3655	88.244
3654	167.3573	3654	143.1936	3654	120.6483	3654	88.26439
3653	167.3948	3653	143.2227	3653	120.6724	3653	88.29427
3652	167.4306	3652	143.2575	3652	120.6953	3652	88.33018
3651	167.4399	3651	143.2605	3651	120.6868	3651	88.34425
3650	167.4259	3650	143.2252	3650	120.6324	3650	88.32252
3649	167.437	3649	143.2195	3649	120.6451	3649	88.31607
3648	167.4665	3648	143.2516	3648	120.7442	3648	88.33828
3647	167.4657	3647	143.2607	3647	120.7928	3647	88.34161
3646	167.4326	3646	143.2364	3646	120.7554	3646	88.31559
3645	167.3939	3645	143.2062	3645	120.6931	3645	88.28322
3644	167.3665	3644	143.1862	3644	120.6534	3644	88.26101

3643	167.354	3643	143.1786	3643	120.653	3643	88.25316
3642	167.3539	3642	143.1819	3642	120.6879	3642	88.25777
3641	167.362	3641	143.1954	3641	120.741	3641	88.27133
3640	167.3737	3640	143.2151	3640	120.7945	3640	88.28978
3639	167.3844	3639	143.2322	3639	120.8376	3639	88.30773
3638	167.3925	3638	143.2409	3638	120.8629	3638	88.32031
3637	167.3982	3637	143.2412	3637	120.8625	3637	88.32398
3636	167.3971	3636	143.2301	3636	120.83	3636	88.31356
3635	167.381	3635	143.1973	3635	120.7702	3635	88.28291
3634	167.3492	3634	143.1406	3634	120.696	3634	88.23312
3633	167.3142	3633	143.0783	3633	120.6124	3633	88.17736
3632	167.2912	3632	143.0365	3632	120.5136	3632	88.13549
3631	167.2855	3631	143.0241	3631	120.4108	3631	88.12288
3630	167.2892	3630	143.0202	3630	120.3483	3630	88.13523
3629	167.3035	3629	143.0131	3629	120.3576	3629	88.15727
3628	167.3351	3628	143.0289	3628	120.4188	3628	88.18774
3627	167.3713	3627	143.0743	3627	120.497	3627	88.22212
3626	167.3976	3626	143.1278	3626	120.5737	3626	88.25056
3625	167.4114	3625	143.1698	3625	120.646	3625	88.2703
3624	167.4233	3624	143.2012	3624	120.7308	3624	88.29022
3623	167.4497	3623	143.2374	3623	120.8401	3623	88.32322
3622	167.4992	3622	143.2909	3622	120.9702	3622	88.37709
3621	167.5602	3621	143.3511	3621	121.1003	3621	88.44207
3620	167.6003	3620	143.3828	3620	121.1822	3620	88.4858
3619	167.593	3619	143.3581	3619	121.1557	3619	88.47793
3618	167.5434	3618	143.2859	3618	121.0143	3618	88.42125
3617	167.4769	3617	143.1955	3617	120.8153	3617	88.341
3616	167.4154	3616	143.1109	3616	120.6242	3616	88.2608
3615	167.3667	3615	143.0409	3615	120.4858	3615	88.19446
3614	167.3279	3614	142.9815	3614	120.4065	3614	88.14543
3613	167.2946	3613	142.9271	3613	120.3487	3613	88.10988
3612	167.2692	3612	142.8824	3612	120.2789	3612	88.08553
3611	167.2551	3611	142.857	3611	120.2091	3611	88.07315
3610	167.251	3610	142.8543	3610	120.181	3610	88.07278
3609	167.2572	3609	142.8757	3609	120.2373	3609	88.08764
3608	167.2795	3608	142.9222	3608	120.3781	3608	88.12198
3607	167.3161	3607	142.9835	3607	120.544	3607	88.16875
3606	167.3537	3606	143.0391	3606	120.6728	3606	88.21102
3605	167.3807	3605	143.0748	3605	120.748	3605	88.23832
3604	167.3943	3604	143.0888	3604	120.7837	3604	88.25224
3603	167.3976	3603	143.0852	3603	120.7909	3603	88.25738
3602	167.3977	3602	143.0732	3602	120.7774	3602	88.25878
3601	167.404	3601	143.0671	3601	120.7637	3601	88.26297
3600	167.421	3600	143.0777	3600	120.7732	3600	88.27508
3599	167.4419	3599	143.0977	3599	120.8027	3599	88.29081

3598	167.4526	3598	143.1048	3598	120.8243	3598	88.29725
3597	167.4426	3597	143.0819	3597	120.8131	3597	88.28234
3596	167.4141	3596	143.0347	3596	120.773	3596	88.24699
3595	167.3799	3595	142.9874	3595	120.7321	3595	88.20727
3594	167.3532	3594	142.9596	3594	120.7078	3594	88.18131
3593	167.3362	3593	142.9486	3593	120.6898	3593	88.17264
3592	167.3249	3592	142.9419	3592	120.6637	3592	88.17354
3591	167.3204	3591	142.9391	3591	120.6399	3591	88.18111
3590	167.328	3590	142.95	3590	120.65	3590	88.19884
3589	167.3486	3589	142.9775	3589	120.7163	3589	88.22482
3588	167.3756	3588	143.0103	3588	120.8077	3588	88.24709
3587	167.4002	3587	143.0338	3587	120.8579	3587	88.25821
3586	167.4123	3586	143.0361	3586	120.8347	3586	88.25605
3585	167.4108	3585	143.0188	3585	120.7756	3585	88.24391
3584	167.4019	3584	142.9939	3584	120.7298	3584	88.22821
3583	167.39	3583	142.971	3583	120.7148	3583	88.2133
3582	167.3785	3582	142.9551	3582	120.7232	3582	88.20135
3581	167.3693	3581	142.9443	3581	120.74	3581	88.19194
3580	167.3621	3580	142.9334	3580	120.7517	3580	88.18316
3579	167.3549	3579	142.9197	3579	120.7528	3579	88.17415
3578	167.3464	3578	142.9056	3578	120.7457	3578	88.16633
3577	167.3383	3577	142.8949	3577	120.7375	3577	88.16154
3576	167.3328	3576	142.887	3576	120.7326	3576	88.15896
3575	167.3309	3575	142.8781	3575	120.7305	3575	88.15568
3574	167.3307	3574	142.8648	3574	120.7268	3574	88.1485
3573	167.33	3573	142.8474	3573	120.7173	3573	88.13675
3572	167.3289	3572	142.8295	3572	120.7034	3572	88.12368
3571	167.3306	3571	142.8182	3571	120.6925	3571	88.11672
3570	167.3385	3570	142.8191	3570	120.694	3570	88.12313
3569	167.3505	3569	142.8279	3569	120.7062	3569	88.13943
3568	167.3633	3568	142.8356	3568	120.7137	3568	88.15283
3567	167.3811	3567	142.8472	3567	120.7118	3567	88.161
3566	167.3972	3566	142.8616	3566	120.697	3566	88.16567
3565	167.3943	3565	142.8621	3565	120.6593	3565	88.15985
3564	167.3718	3564	142.8459	3564	120.6144	3564	88.14333
3563	167.3439	3563	142.8243	3563	120.5915	3563	88.12519
3562	167.3231	3562	142.8068	3562	120.6025	3562	88.11283
3561	167.3133	3561	142.7954	3561	120.637	3561	88.1074
3560	167.3107	3560	142.7877	3560	120.6754	3560	88.10569
3559	167.3093	3559	142.7818	3559	120.7041	3559	88.10465
3558	167.3073	3558	142.7765	3558	120.7191	3558	88.10348
3557	167.3063	3557	142.7701	3557	120.7231	3557	88.10248
3556	167.3082	3556	142.7613	3556	120.7211	3556	88.1015
3555	167.313	3555	142.7509	3555	120.7201	3555	88.1005
3554	167.3185	3554	142.7414	3554	120.7255	3554	88.10014

3553	167.3217	3553	142.7333	3553	120.7328	3553	88.101
3552	167.32	3552	142.7221	3552	120.7248	3552	88.10078
3551	167.3131	3551	142.7025	3551	120.6887	3551	88.09566
3550	167.3037	3550	142.6752	3550	120.6326	3550	88.08581
3549	167.2957	3549	142.6472	3549	120.5782	3549	88.07654
3548	167.2902	3548	142.6264	3548	120.5414	3548	88.07252
3547	167.2867	3547	142.6193	3547	120.5263	3547	88.07376
3546	167.2861	3546	142.6285	3546	120.5325	3546	88.07685
3545	167.2897	3545	142.6484	3545	120.5587	3545	88.07876
3544	167.2957	3544	142.6675	3544	120.5974	3544	88.07846
3543	167.3007	3543	142.6783	3543	120.6375	3543	88.0769
3542	167.3019	3542	142.68	3542	120.6702	3542	88.0746
3541	167.2997	3541	142.675	3541	120.6914	3541	88.07114
3540	167.2965	3540	142.6659	3540	120.703	3540	88.0667
3539	167.2944	3539	142.6538	3539	120.7091	3539	88.06172
3538	167.2932	3538	142.6393	3538	120.7136	3538	88.05614
3537	167.2913	3537	142.6238	3537	120.7167	3537	88.04923
3536	167.2881	3536	142.61	3536	120.716	3536	88.04207
3535	167.2844	3535	142.5996	3535	120.7094	3535	88.03753
3534	167.2815	3534	142.5914	3534	120.6987	3534	88.03784
3533	167.2796	3533	142.5817	3533	120.6857	3533	88.04103
3532	167.2766	3532	142.5662	3532	120.6675	3532	88.04141
3531	167.27	3531	142.5437	3531	120.6399	3531	88.03406
3530	167.2589	3530	142.5167	3530	120.6031	3530	88.01875
3529	167.2464	3529	142.49	3529	120.5637	3529	88.00009
3528	167.2374	3528	142.4684	3528	120.5278	3528	87.98349
3527	167.2342	3527	142.4542	3527	120.4968	3527	87.97144
3526	167.2349	3526	142.4473	3526	120.4728	3526	87.96394
3525	167.2368	3525	142.4476	3525	120.465	3525	87.96161
3524	167.2401	3524	142.4553	3524	120.4829	3524	87.96582
3523	167.2466	3523	142.4693	3523	120.5227	3523	87.97594
3522	167.2547	3522	142.4837	3522	120.5674	3522	87.98736
3521	167.261	3521	142.4908	3521	120.6036	3521	87.995
3520	167.2641	3520	142.4867	3520	120.6314	3520	87.99739
3519	167.2651	3519	142.4737	3519	120.6565	3519	87.99693
3518	167.2669	3518	142.4593	3518	120.6801	3518	87.99789
3517	167.27	3517	142.4491	3517	120.697	3517	88.00271
3516	167.2718	3516	142.4425	3516	120.701	3516	88.00964
3515	167.2684	3515	142.4334	3515	120.6908	3515	88.0138
3514	167.259	3514	142.4185	3514	120.6709	3514	88.01138
3513	167.2467	3513	142.4011	3513	120.649	3513	88.0028
3512	167.2369	3512	142.3891	3512	120.6334	3512	87.99207
3511	167.2332	3511	142.387	3511	120.6303	3511	87.98308
3510	167.2356	3510	142.3918	3510	120.6389	3510	87.97711
3509	167.2407	3509	142.3938	3509	120.6495	3509	87.97297

3508	167.2451	3508	142.3849	3508	120.6503	3508	87.96945
3507	167.2477	3507	142.3665	3507	120.6371	3507	87.96657
3506	167.2485	3506	142.3462	3506	120.612	3506	87.96363
3505	167.247	3505	142.3325	3505	120.58	3505	87.95888
3504	167.244	3504	142.3299	3504	120.5512	3504	87.95302
3503	167.2414	3503	142.3359	3503	120.5377	3503	87.94985
3502	167.2396	3502	142.3414	3502	120.5426	3502	87.95059
3501	167.2389	3501	142.3407	3501	120.5598	3501	87.9525
3500	167.2413	3500	142.3366	3500	120.5844	3500	87.95314
3499	167.2475	3499	142.3342	3499	120.6127	3499	87.95181
3498	167.2552	3498	142.3333	3498	120.639	3498	87.94837
3497	167.2601	3497	142.3293	3497	120.6568	3497	87.94343
3496	167.2603	3496	142.32	3496	120.664	3496	87.93952
3495	167.2569	3495	142.3082	3495	120.664	3495	87.93913
3494	167.2513	3494	142.2978	3494	120.6619	3494	87.94106
3493	167.2436	3493	142.2893	3493	120.6593	3493	87.9403
3492	167.2335	3492	142.2796	3492	120.6539	3492	87.93278
3491	167.2225	3491	142.2665	3491	120.6435	3491	87.9197
3490	167.2134	3490	142.252	3490	120.6295	3490	87.90653
3489	167.2088	3489	142.2411	3489	120.6173	3489	87.89858
3488	167.211	3488	142.2376	3488	120.6123	3488	87.89773
3487	167.2195	3487	142.2397	3487	120.6156	3487	87.90237
3486	167.2307	3486	142.242	3486	120.6227	3486	87.90913
3485	167.2384	3485	142.2388	3485	120.6267	3485	87.91385
3484	167.2369	3484	142.2278	3484	120.6227	3484	87.91305
3483	167.226	3483	142.2118	3483	120.6117	3483	87.90665
3482	167.2128	3482	142.198	3482	120.6027	3482	87.89916
3481	167.2072	3481	142.1924	3481	120.6059	3481	87.8957
3480	167.2134	3480	142.1943	3480	120.6229	3480	87.89678
3479	167.2275	3479	142.198	3479	120.6468	3479	87.89887
3478	167.2414	3478	142.1988	3478	120.6689	3478	87.89857
3477	167.2495	3477	142.1966	3477	120.6837	3477	87.89554
3476	167.2508	3476	142.1941	3476	120.6905	3476	87.89198
3475	167.2474	3475	142.192	3475	120.6901	3475	87.88998
3474	167.2413	3474	142.1876	3474	120.6832	3474	87.88952
3473	167.2343	3473	142.1781	3473	120.6714	3473	87.88921
3472	167.2294	3472	142.1651	3472	120.6584	3472	87.88883
3471	167.2304	3471	142.154	3471	120.6496	3471	87.88955
3470	167.2378	3470	142.1485	3470	120.6484	3470	87.89141
3469	167.2476	3469	142.1473	3469	120.6533	3469	87.89173
3468	167.2537	3468	142.1455	3468	120.6588	3468	87.88747
3467	167.2525	3467	142.1384	3467	120.6587	3467	87.8788
3466	167.2445	3466	142.1245	3466	120.6505	3466	87.86919
3465	167.2324	3465	142.1056	3465	120.6369	3465	87.8615
3464	167.2192	3464	142.0859	3464	120.6235	3464	87.85562

3463	167.2078	3463	142.0705	3463	120.6155	3463	87.85002
3462	167.2004	3462	142.0635	3462	120.6156	3462	87.84472
3461	167.1973	3461	142.0657	3461	120.6243	3461	87.84126
3460	167.1965	3460	142.074	3460	120.6394	3460	87.84037
3459	167.1949	3459	142.0829	3459	120.6569	3459	87.84055
3458	167.1907	3458	142.0873	3458	120.672	3458	87.83855
3457	167.1836	3457	142.0845	3457	120.6815	3457	87.83125
3456	167.1756	3456	142.0759	3456	120.6865	3456	87.81876
3455	167.1692	3455	142.0668	3455	120.6912	3455	87.80523
3454	167.1655	3454	142.0616	3454	120.6972	3454	87.79596
3453	167.1633	3453	142.0596	3453	120.7007	3453	87.7926
3452	167.1613	3452	142.055	3452	120.6947	3452	87.79254
3451	167.1597	3451	142.0436	3451	120.6783	3451	87.79305
3450	167.1596	3450	142.0265	3450	120.6587	3450	87.79373
3449	167.1605	3449	142.0083	3449	120.646	3449	87.79442
3448	167.1601	3448	141.9928	3448	120.6437	3448	87.79317
3447	167.1572	3447	141.9816	3447	120.6452	3447	87.78813
3446	167.153	3446	141.9741	3446	120.6405	3446	87.78041
3445	167.1501	3445	141.97	3445	120.6283	3445	87.77358
3444	167.1496	3444	141.9692	3444	120.6177	3444	87.77021
3443	167.1504	3443	141.9705	3443	120.6175	3443	87.76999
3442	167.1503	3442	141.9718	3442	120.6272	3442	87.77131
3441	167.1484	3441	141.9713	3441	120.6393	3441	87.77369
3440	167.1453	3440	141.9681	3440	120.6473	3440	87.7777
3439	167.1427	3439	141.962	3439	120.6501	3439	87.7832
3438	167.1421	3438	141.9533	3438	120.6501	3438	87.78852
3437	167.1441	3437	141.9428	3437	120.6497	3437	87.79153
3436	167.1489	3436	141.9319	3436	120.6506	3436	87.79211
3435	167.1549	3435	141.9217	3435	120.6539	3435	87.79204
3434	167.1585	3434	141.9115	3434	120.658	3434	87.79235
3433	167.1563	3433	141.8997	3433	120.6597	3433	87.79111
3432	167.1483	3432	141.8858	3432	120.6563	3432	87.78505
3431	167.1387	3431	141.8721	3431	120.6499	3431	87.77397
3430	167.1325	3430	141.8617	3430	120.6451	3430	87.76222
3429	167.132	3429	141.8556	3429	120.6446	3429	87.7559
3428	167.1365	3428	141.8523	3428	120.6483	3428	87.75885
3427	167.144	3427	141.8498	3427	120.6542	3427	87.77061
3426	167.1517	3426	141.8464	3426	120.658	3426	87.78628
3425	167.1566	3425	141.8403	3425	120.6547	3425	87.79857
3424	167.1575	3424	141.8315	3424	120.6415	3424	87.8019
3423	167.1559	3423	141.8219	3423	120.6216	3423	87.79561
3422	167.153	3422	141.814	3422	120.6023	3422	87.78347
3421	167.1483	3421	141.8078	3421	120.5903	3421	87.77
3420	167.1423	3420	141.8011	3420	120.5882	3420	87.75816
3419	167.1383	3419	141.7925	3419	120.5968	3419	87.75011

3418	167.1405	3418	141.7823	3418	120.6146	3418	87.7478
3417	167.1486	3417	141.7713	3417	120.6356	3417	87.75115
3416	167.1581	3416	141.7598	3416	120.6509	3416	87.75718
3415	167.1646	3415	141.7503	3415	120.6576	3415	87.76221
3414	167.1667	3414	141.7459	3414	120.661	3414	87.76452
3413	167.166	3413	141.7472	3413	120.6678	3413	87.76451
3412	167.1657	3412	141.7515	3412	120.6782	3412	87.76358
3411	167.1693	3411	141.755	3411	120.6879	3411	87.76337
3410	167.1777	3410	141.7558	3410	120.6935	3410	87.76482
3409	167.1874	3409	141.7538	3409	120.6948	3409	87.76699
3408	167.1931	3408	141.7484	3408	120.6931	3408	87.76722
3407	167.1914	3407	141.7394	3407	120.6902	3407	87.76358
3406	167.1837	3406	141.7279	3406	120.6877	3406	87.75709
3405	167.1734	3405	141.7164	3405	120.6865	3405	87.75104
3404	167.1644	3404	141.7064	3404	120.6864	3404	87.74778
3403	167.1588	3403	141.6963	3403	120.6857	3403	87.74607
3402	167.1557	3402	141.6829	3402	120.6816	3402	87.74213
3401	167.1526	3401	141.6659	3401	120.6731	3401	87.73381
3400	167.1479	3400	141.6497	3400	120.6626	3400	87.72329
3399	167.1434	3399	141.6398	3399	120.6555	3399	87.71532
3398	167.142	3398	141.6364	3398	120.6538	3398	87.71264
3397	167.144	3397	141.6344	3397	120.6534	3397	87.71401
3396	167.1471	3396	141.6295	3396	120.649	3396	87.71678
3395	167.1499	3395	141.6221	3395	120.6416	3395	87.72018
3394	167.1535	3394	141.6152	3394	120.6376	3394	87.72515
3393	167.1592	3393	141.6097	3393	120.641	3393	87.732
3392	167.1656	3392	141.6039	3392	120.6496	3392	87.73907
3391	167.1699	3391	141.5962	3391	120.6574	3391	87.74332
3390	167.1702	3390	141.5871	3390	120.6617	3390	87.74185
3389	167.1674	3389	141.579	3389	120.6635	3389	87.73401
3388	167.1644	3388	141.5732	3388	120.6651	3388	87.72327
3387	167.1644	3387	141.5699	3387	120.6685	3387	87.71588
3386	167.1678	3386	141.5675	3386	120.6743	3386	87.71557
3385	167.1721	3385	141.5647	3385	120.6809	3385	87.71955
3384	167.1755	3384	141.5614	3384	120.6859	3384	87.72197
3383	167.1776	3383	141.558	3383	120.6887	3383	87.72065
3382	167.1783	3382	141.554	3382	120.6899	3382	87.71825
3381	167.1747	3381	141.5462	3381	120.689	3381	87.71677
3380	167.165	3380	141.5325	3380	120.6847	3380	87.71416
3379	167.1526	3379	141.515	3379	120.6778	3379	87.70769
3378	167.1453	3378	141.4999	3378	120.673	3378	87.69833
3377	167.1479	3377	141.4924	3377	120.6745	3377	87.68983
3376	167.1574	3376	141.4918	3376	120.6818	3376	87.68453
3375	167.1665	3375	141.4924	3375	120.6911	3375	87.68212
3374	167.1705	3374	141.4893	3374	120.6994	3374	87.6815

3373	167.1696	3373	141.4819	3373	120.706	3373	87.68186
3372	167.1663	3372	141.472	3372	120.7099	3372	87.68195
3371	167.1624	3371	141.4609	3371	120.7082	3371	87.6805
3370	167.1584	3370	141.4486	3370	120.6985	3370	87.67822
3369	167.154	3369	141.4355	3369	120.6819	3369	87.67781
3368	167.1492	3368	141.4231	3368	120.6639	3368	87.68077
3367	167.1443	3367	141.4134	3367	120.6511	3367	87.68485
3366	167.1403	3366	141.4067	3366	120.6465	3366	87.68563
3365	167.1381	3365	141.4014	3365	120.647	3365	87.68062
3364	167.1377	3364	141.3954	3364	120.6474	3364	87.67138
3363	167.1387	3363	141.3876	3363	120.6447	3363	87.66185
3362	167.1408	3362	141.378	3362	120.6407	3362	87.65495
3361	167.1434	3361	141.3675	3361	120.6383	3361	87.6508
3360	167.1471	3360	141.3573	3360	120.6389	3360	87.64783
3359	167.1531	3359	141.3486	3359	120.6433	3359	87.64533
3358	167.1617	3358	141.3429	3358	120.653	3358	87.64471
3357	167.1701	3357	141.3414	3357	120.6684	3357	87.64796
3356	167.1732	3356	141.3441	3356	120.6861	3356	87.65485
3355	167.1683	3355	141.3478	3355	120.6997	3355	87.66189
3354	167.1578	3354	141.3473	3354	120.705	3354	87.6644
3353	167.1475	3353	141.3395	3353	120.7032	3353	87.66015
3352	167.1425	3352	141.3258	3352	120.699	3352	87.65085
3351	167.143	3351	141.3096	3351	120.6968	3351	87.64003
3350	167.1446	3350	141.2926	3350	120.6979	3350	87.6295
3349	167.142	3349	141.2748	3349	120.701	3349	87.61868
3348	167.1337	3348	141.2578	3348	120.7047	3348	87.6076
3347	167.1235	3347	141.2457	3347	120.7079	3347	87.59916
3346	167.1179	3346	141.2409	3346	120.7101	3346	87.59708
3345	167.1195	3345	141.2403	3345	120.7102	3345	87.60159
3344	167.1259	3344	141.238	3344	120.7068	3344	87.60852
3343	167.1321	3343	141.2315	3343	120.7	3343	87.61279
3342	167.1355	3342	141.223	3342	120.6914	3342	87.61204
3341	167.1374	3341	141.2159	3341	120.6833	3341	87.60733
3340	167.1401	3340	141.212	3340	120.6774	3340	87.60185
3339	167.1448	3339	141.2109	3339	120.6755	3339	87.59922
3338	167.1498	3338	141.2117	3338	120.679	3338	87.60134
3337	167.1517	3337	141.212	3337	120.6873	3337	87.60658
3336	167.1487	3336	141.2089	3336	120.6968	3336	87.61071
3335	167.1415	3335	141.1999	3335	120.7032	3335	87.61071
3334	167.1329	3334	141.1859	3334	120.7053	3334	87.60719
3333	167.1251	3333	141.1697	3333	120.7054	3333	87.60284
3332	167.1192	3332	141.1544	3332	120.706	3332	87.5994
3331	167.1153	3331	141.142	3331	120.7066	3331	87.59656
3330	167.1129	3330	141.1325	3330	120.7046	3330	87.59233
3329	167.11	3329	141.1241	3329	120.6979	3329	87.58407

3328	167.1056	3328	141.114	3328	120.6881	3328	87.57078
3327	167.1017	3327	141.1014	3327	120.6797	3327	87.55564
3326	167.1018	3326	141.0892	3326	120.6771	3326	87.54508
3325	167.1069	3325	141.081	3325	120.6816	3325	87.54336
3324	167.1126	3324	141.0773	3324	120.6894	3324	87.54807
3323	167.1139	3323	141.0744	3323	120.6951	3323	87.5522
3322	167.1099	3322	141.0682	3322	120.6962	3322	87.55049
3321	167.104	3321	141.0584	3321	120.6941	3321	87.54335
3320	167.1003	3320	141.0477	3320	120.6919	3320	87.53545
3319	167.1006	3319	141.0381	3319	120.6908	3319	87.53161
3318	167.1041	3318	141.03	3318	120.6898	3318	87.53368
3317	167.1091	3317	141.0237	3317	120.6886	3317	87.5398
3316	167.1138	3316	141.0208	3316	120.6881	3316	87.54603
3315	167.1173	3315	141.0222	3315	120.6892	3315	87.54931
3314	167.1187	3314	141.0249	3314	120.6903	3314	87.54925
3313	167.1168	3313	141.0238	3313	120.6877	3313	87.54695
3312	167.1109	3312	141.0154	3312	120.6795	3312	87.5429
3311	167.1028	3311	141.0007	3311	120.6684	3311	87.53681
3310	167.0969	3310	140.9842	3310	120.6606	3310	87.52929
3309	167.0974	3309	140.9711	3309	120.6608	3309	87.52276
3308	167.1041	3308	140.9645	3308	120.6689	3308	87.51992
3307	167.1125	3307	140.9628	3307	120.6811	3307	87.52141
3306	167.1169	3306	140.9609	3306	120.6929	3306	87.52493
3305	167.1147	3305	140.9543	3305	120.7016	3305	87.52652
3304	167.1074	3304	140.9431	3304	120.7067	3304	87.52355
3303	167.0998	3303	140.9309	3303	120.7098	3303	87.51686
3302	167.0964	3302	140.9208	3302	120.713	3302	87.50996
3301	167.0976	3301	140.9127	3301	120.7171	3301	87.50565
3300	167.0997	3300	140.9061	3300	120.7206	3300	87.50407
3299	167.0989	3299	140.9019	3299	120.7219	3299	87.5037
3298	167.0935	3298	140.901	3298	120.7193	3298	87.50306
3297	167.0847	3297	140.9013	3297	120.7117	3297	87.50088
3296	167.0754	3296	140.8993	3296	120.698	3296	87.49599
3295	167.0689	3295	140.8943	3295	120.6797	3295	87.48883
3294	167.0671	3294	140.8887	3294	120.6614	3294	87.48249
3293	167.0689	3293	140.8847	3293	120.6488	3293	87.48078
3292	167.072	3292	140.8822	3292	120.6453	3292	87.48493
3291	167.0755	3291	140.8802	3291	120.6507	3291	87.49308
3290	167.0808	3290	140.8793	3290	120.6626	3290	87.50278
3289	167.0881	3289	140.881	3289	120.6774	3289	87.51311
3288	167.0957	3288	140.8861	3288	120.6919	3288	87.52421
3287	167.1021	3287	140.8936	3287	120.7047	3287	87.5358
3286	167.1078	3286	140.9014	3286	120.7162	3286	87.54613
3285	167.1124	3285	140.9062	3285	120.7263	3285	87.5519
3284	167.1134	3284	140.9056	3284	120.7328	3284	87.55004

3283	167.1086	3283	140.8996	3283	120.7334	3283	87.54087
3282	167.1003	3282	140.8921	3282	120.7289	3282	87.5294
3281	167.0941	3281	140.8883	3281	120.7227	3281	87.52192
3280	167.0936	3280	140.8907	3280	120.7162	3280	87.5211
3279	167.0987	3279	140.8973	3279	120.7087	3279	87.52515
3278	167.1076	3278	140.9042	3278	120.6998	3278	87.53174
3277	167.1187	3277	140.9097	3277	120.6933	3277	87.54086
3276	167.1296	3276	140.9159	3276	120.6936	3276	87.55304
3275	167.1375	3275	140.9255	3275	120.7016	3275	87.56669
3274	167.1405	3274	140.939	3274	120.7128	3274	87.57901
3273	167.1395	3273	140.9535	3273	120.7219	3273	87.589
3272	167.1365	3272	140.9653	3272	120.7264	3272	87.59758
3271	167.1326	3271	140.9711	3271	120.7268	3271	87.60452
3270	167.1274	3270	140.9693	3270	120.7241	3270	87.60706
3269	167.1204	3269	140.9604	3269	120.7199	3269	87.6025
3268	167.1128	3268	140.9481	3268	120.7156	3268	87.5919
3267	167.1071	3267	140.9384	3267	120.7129	3267	87.58067
3266	167.106	3266	140.9375	3266	120.7131	3266	87.57562
3265	167.1108	3265	140.9479	3265	120.7172	3265	87.58114
3264	167.1211	3264	140.9673	3264	120.7251	3264	87.59661
3263	167.1351	3263	140.9892	3263	120.7343	3263	87.61645
3262	167.1498	3262	141.0068	3262	120.7408	3262	87.63286
3261	167.1606	3261	141.0157	3261	120.741	3261	87.64003
3260	167.1626	3260	141.0163	3260	120.7343	3260	87.63722
3259	167.155	3259	141.0126	3259	120.7229	3259	87.62855
3258	167.1437	3258	141.0101	3258	120.7116	3258	87.62036
3257	167.1372	3257	141.0132	3257	120.707	3257	87.61819
3256	167.14	3256	141.0235	3256	120.7138	3256	87.62452
3255	167.1492	3255	141.0384	3255	120.73	3255	87.63732
3254	167.158	3254	141.0523	3254	120.7458	3254	87.6509
3253	167.161	3253	141.0609	3253	120.7503	3253	87.65981
3252	167.1577	3252	141.065	3252	120.7408	3252	87.66333
3251	167.1511	3251	141.0692	3251	120.7253	3251	87.66574
3250	167.1453	3250	141.0774	3250	120.7144	3250	87.67126
3249	167.1424	3249	141.0894	3249	120.713	3249	87.67946
3248	167.142	3248	141.1025	3248	120.7186	3248	87.68649
3247	167.1436	3247	141.1143	3247	120.7262	3247	87.69043
3246	167.1488	3246	141.1244	3246	120.7331	3246	87.69406
3245	167.1599	3245	141.1336	3245	120.7394	3245	87.70179
3244	167.1758	3244	141.1424	3244	120.7461	3244	87.71467
3243	167.1913	3243	141.1512	3243	120.7531	3243	87.72976
3242	167.2009	3242	141.1598	3242	120.7602	3242	87.7432
3241	167.2013	3241	141.168	3241	120.7668	3241	87.75263
3240	167.1926	3240	141.1749	3240	120.7722	3240	87.75722
3239	167.1783	3239	141.1797	3239	120.775	3239	87.75727

3238	167.1649	3238	141.1828	3238	120.7743	3238	87.75465
3237	167.1585	3237	141.185	3237	120.7691	3237	87.75241
3236	167.1614	3236	141.1877	3236	120.7595	3236	87.75302
3235	167.17	3235	141.1924	3235	120.7473	3235	87.75667
3234	167.1789	3234	141.1998	3234	120.7368	3234	87.76185
3233	167.1843	3233	141.2093	3233	120.7328	3233	87.76714
3232	167.1849	3232	141.2187	3232	120.7368	3232	87.77204
3231	167.1814	3231	141.2253	3231	120.7453	3231	87.77626
3230	167.176	3230	141.2285	3230	120.7545	3230	87.77905
3229	167.1726	3229	141.231	3229	120.7638	3229	87.78055
3228	167.1744	3228	141.2374	3228	120.7752	3228	87.78329
3227	167.1816	3227	141.2504	3227	120.7876	3227	87.7904
3226	167.1913	3226	141.2674	3226	120.7968	3226	87.80114
3225	167.1989	3225	141.282	3225	120.799	3225	87.80952
3224	167.2012	3224	141.2892	3224	120.7941	3224	87.80932
3223	167.1981	3223	141.2887	3223	120.7855	3223	87.80093
3222	167.1924	3222	141.2848	3222	120.7765	3222	87.79138
3221	167.1888	3221	141.282	3221	120.7704	3221	87.78745
3220	167.1899	3220	141.2819	3220	120.7692	3220	87.79012
3219	167.195	3219	141.283	3219	120.7721	3219	87.79646
3218	167.2006	3218	141.2835	3218	120.7759	3218	87.8044
3217	167.2024	3217	141.2831	3217	120.7775	3217	87.8131
3216	167.1981	3216	141.2827	3216	120.7771	3216	87.8203
3215	167.19	3215	141.2841	3215	120.7769	3215	87.82345
3214	167.1847	3214	141.2899	3214	120.7794	3214	87.82398
3213	167.1882	3213	141.3014	3213	120.7854	3213	87.82716
3212	167.1992	3212	141.3171	3212	120.794	3212	87.83591
3211	167.2095	3211	141.3324	3211	120.8032	3211	87.84719
3210	167.2125	3210	141.3443	3210	120.8105	3210	87.85603
3209	167.2091	3209	141.3544	3209	120.8155	3209	87.86166
3208	167.2049	3208	141.3663	3208	120.8193	3208	87.86738
3207	167.2032	3207	141.3809	3207	120.8223	3207	87.87491
3206	167.2036	3206	141.3947	3206	120.8234	3206	87.88152
3205	167.2057	3205	141.4038	3205	120.8215	3205	87.88339
3204	167.2114	3204	141.4084	3204	120.819	3204	87.88045
3203	167.2219	3203	141.4115	3203	120.8204	3203	87.87694
3202	167.2343	3202	141.4155	3202	120.8285	3202	87.87711
3201	167.2435	3201	141.4197	3201	120.8406	3201	87.88146
3200	167.246	3200	141.4229	3200	120.8505	3200	87.88691
3199	167.2429	3199	141.4261	3199	120.852	3199	87.89018
3198	167.2369	3198	141.4307	3198	120.844	3198	87.89075
3197	167.2311	3197	141.4354	3197	120.8317	3197	87.89079
3196	167.2266	3196	141.438	3196	120.8226	3196	87.8931
3195	167.2241	3195	141.4386	3195	120.8213	3195	87.89926
3194	167.2241	3194	141.4407	3194	120.8277	3194	87.90908

3193	167.2272	3193	141.4476	3193	120.8384	3193	87.92072
3192	167.2333	3192	141.4596	3192	120.8494	3192	87.93173
3191	167.2416	3191	141.475	3191	120.8576	3191	87.94039
3190	167.2503	3190	141.4926	3190	120.8603	3190	87.9463
3189	167.2576	3189	141.5113	3189	120.8571	3189	87.9499
3188	167.2614	3188	141.5288	3188	120.85	3188	87.9514
3187	167.2612	3187	141.5429	3187	120.8429	3187	87.95076
3186	167.2578	3186	141.553	3186	120.8393	3186	87.94833
3185	167.253	3185	141.5603	3185	120.8404	3185	87.94552
3184	167.2483	3184	141.5661	3184	120.8441	3184	87.94493
3183	167.2441	3183	141.5701	3183	120.8468	3183	87.94926
3182	167.2408	3182	141.572	3182	120.846	3182	87.95894
3181	167.2393	3181	141.5733	3181	120.8431	3181	87.97047
3180	167.2411	3180	141.5771	3180	120.8422	3180	87.97879
3179	167.247	3179	141.5868	3179	120.8474	3179	87.98227
3178	167.2557	3178	141.6031	3178	120.8589	3178	87.9843
3177	167.2629	3177	141.6215	3177	120.8725	3177	87.98871
3176	167.2637	3176	141.6357	3176	120.8824	3176	87.99531
3175	167.2577	3175	141.6432	3175	120.8866	3175	88.00175
3174	167.2499	3174	141.6485	3174	120.8875	3174	88.00775
3173	167.2473	3173	141.6574	3173	120.8896	3173	88.01491
3172	167.2528	3172	141.6708	3172	120.8941	3172	88.02288
3171	167.2622	3171	141.6839	3171	120.8991	3171	88.02856
3170	167.2695	3170	141.6926	3170	120.9018	3170	88.02981
3169	167.2718	3169	141.6977	3169	120.902	3169	88.028
3168	167.2705	3168	141.7032	3168	120.9014	3168	88.02635
3167	167.2687	3167	141.712	3167	120.9013	3167	88.02724
3166	167.268	3166	141.7245	3166	120.9022	3166	88.03163
3165	167.2686	3165	141.7387	3165	120.9038	3165	88.03896
3164	167.2703	3164	141.7511	3164	120.9056	3164	88.04706
3163	167.2726	3163	141.7589	3163	120.9072	3163	88.05365
3162	167.2746	3162	141.7635	3162	120.9091	3162	88.0591
3161	167.2753	3161	141.7694	3161	120.913	3161	88.06599
3160	167.2747	3160	141.7796	3160	120.9203	3160	88.07526
3159	167.2742	3159	141.7925	3159	120.9292	3159	88.08407
3158	167.2746	3158	141.8048	3158	120.936	3158	88.08865
3157	167.2766	3157	141.8157	3157	120.9376	3157	88.0885
3156	167.281	3156	141.827	3156	120.9352	3156	88.08668
3155	167.2886	3155	141.8399	3155	120.9325	3155	88.08666
3154	167.2973	3154	141.8525	3154	120.9319	3154	88.08963
3153	167.301	3153	141.8608	3153	120.9324	3153	88.09401
3152	167.2944	3152	141.8622	3152	120.9306	3152	88.097
3151	167.2793	3151	141.8577	3151	120.9255	3151	88.09697
3150	167.265	3150	141.8525	3150	120.9202	3150	88.09543
3149	167.2595	3149	141.8532	3149	120.919	3149	88.09603

3148	167.2634	3148	141.8632	3148	120.9234	3148	88.10105
3147	167.2712	3147	141.8808	3147	120.9311	3147	88.10908
3146	167.2776	3146	141.9007	3146	120.9384	3146	88.1168
3145	167.2812	3145	141.9175	3145	120.9425	3145	88.12277
3144	167.2832	3144	141.9287	3144	120.9429	3144	88.1285
3143	167.2847	3143	141.9347	3143	120.9414	3143	88.13546
3142	167.287	3142	141.9375	3142	120.9406	3142	88.14197
3141	167.2911	3141	141.9396	3141	120.9429	3141	88.14423
3140	167.2977	3140	141.9432	3140	120.9478	3140	88.14096
3139	167.3061	3139	141.9492	3139	120.953	3139	88.13629
3138	167.3144	3138	141.9579	3138	120.9564	3138	88.13666
3137	167.3198	3137	141.9677	3137	120.9576	3137	88.14419
3136	167.3202	3136	141.9764	3136	120.957	3136	88.15421
3135	167.3165	3135	141.9821	3135	120.9553	3135	88.16024
3134	167.3127	3134	141.9859	3134	120.9535	3134	88.16126
3133	167.3123	3133	141.9902	3133	120.9534	3133	88.16215
3132	167.315	3132	141.9953	3132	120.9552	3132	88.16694
3131	167.3176	3131	141.9983	3131	120.9573	3131	88.17347
3130	167.3177	3130	141.9972	3130	120.9588	3130	88.17608
3129	167.3167	3129	141.9937	3129	120.9603	3129	88.17184
3128	167.3177	3128	141.9917	3128	120.9615	3128	88.1631
3127	167.3225	3127	141.993	3127	120.96	3127	88.15522
3126	167.3296	3126	141.9966	3126	120.9546	3126	88.15259
3125	167.3356	3125	142.0017	3125	120.9479	3125	88.15658
3124	167.3385	3124	142.0093	3124	120.9446	3124	88.16616
3123	167.3397	3123	142.0209	3123	120.9467	3123	88.17934
3122	167.3421	3122	142.0356	3122	120.9515	3122	88.1938
3121	167.3462	3121	142.0497	3121	120.9545	3121	88.20625
3120	167.3486	3120	142.0587	3120	120.9528	3120	88.21251
3119	167.3461	3119	142.0603	3119	120.9457	3119	88.20999
3118	167.339	3118	142.0556	3118	120.9352	3118	88.20056
3117	167.3316	3117	142.0485	3117	120.9255	3117	88.19042
3116	167.3286	3116	142.0443	3116	120.9213	3116	88.18601
3115	167.3316	3115	142.0467	3115	120.9256	3115	88.18947
3114	167.3387	3114	142.0561	3114	120.9375	3114	88.19809
3113	167.3458	3113	142.0689	3113	120.9526	3113	88.20743
3112	167.3492	3112	142.0806	3112	120.9657	3112	88.2146
3111	167.3485	3111	142.0886	3111	120.9732	3111	88.21887
3110	167.3458	3110	142.0921	3110	120.9758	3110	88.22
3109	167.3429	3109	142.0908	3109	120.9762	3109	88.21699
3108	167.3391	3108	142.0845	3108	120.9765	3108	88.20954
3107	167.3338	3107	142.075	3107	120.9777	3107	88.20023
3106	167.3277	3106	142.0659	3106	120.9793	3106	88.19319
3105	167.3229	3105	142.0593	3105	120.9795	3105	88.19035
3104	167.3215	3104	142.055	3104	120.9765	3104	88.19022

3103	167.324	3103	142.0525	3103	120.9708	3103	88.19095
3102	167.329	3102	142.0522	3102	120.9657	3102	88.19263
3101	167.3326	3101	142.0535	3101	120.9635	3101	88.19574
3100	167.3309	3100	142.054	3100	120.9623	3100	88.19912
3099	167.3246	3099	142.0518	3099	120.9591	3099	88.20114
3098	167.3181	3098	142.0478	3098	120.9529	3098	88.2015
3097	167.3148	3097	142.0438	3097	120.9451	3097	88.2005
3096	167.315	3096	142.0409	3096	120.939	3096	88.1979
3095	167.3183	3095	142.0402	3095	120.9391	3095	88.19414
3094	167.3249	3094	142.0441	3094	120.9502	3094	88.1918
3093	167.3334	3093	142.0534	3093	120.9718	3093	88.19343
3092	167.3394	3092	142.0641	3092	120.9954	3092	88.19805
3091	167.3392	3091	142.0697	3091	121.0106	3091	88.20154
3090	167.3344	3090	142.0663	3090	121.0133	3090	88.20078
3089	167.3295	3089	142.0562	3089	121.0081	3089	88.19661
3088	167.327	3088	142.0453	3088	121.0017	3088	88.19281
3087	167.3265	3087	142.039	3087	120.9983	3087	88.19323
3086	167.3269	3086	142.0392	3086	120.9988	3086	88.19933
3085	167.3286	3085	142.0432	3085	121.0022	3085	88.20909
3084	167.3319	3084	142.0462	3084	121.0064	3084	88.21824
3083	167.3367	3083	142.0459	3083	121.0095	3083	88.22358
3082	167.3427	3082	142.0439	3082	121.011	3082	88.22554
3081	167.3484	3081	142.0429	3081	121.0111	3081	88.22665
3080	167.3507	3080	142.0418	3080	121.0095	3080	88.22808
3079	167.348	3079	142.0384	3079	121.0054	3079	88.22902
3078	167.3432	3078	142.0337	3078	121.0011	3078	88.22904
3077	167.341	3077	142.0311	3077	121.0011	3077	88.22885
3076	167.3427	3076	142.031	3076	121.0071	3076	88.22804
3075	167.3452	3075	142.0299	3075	121.0163	3075	88.22453
3074	167.3457	3074	142.0256	3074	121.0249	3074	88.2177
3073	167.3447	3073	142.0219	3073	121.0317	3073	88.2111
3072	167.3446	3072	142.0238	3072	121.0372	3072	88.21012
3071	167.347	3071	142.0307	3071	121.0407	3071	88.2168
3070	167.3512	3070	142.037	3070	121.0401	3070	88.22757
3069	167.3556	3069	142.0385	3069	121.033	3069	88.23618
3068	167.3579	3068	142.0359	3068	121.0197	3068	88.23879
3067	167.3571	3067	142.0322	3067	121.0043	3067	88.23633
3066	167.3547	3066	142.0297	3066	120.9942	3066	88.23273
3065	167.3535	3065	142.0299	3065	120.9954	3065	88.23149
3064	167.3548	3064	142.0335	3064	121.0078	3064	88.23378
3063	167.3581	3063	142.04	3063	121.0248	3063	88.23886
3062	167.3625	3062	142.0469	3062	121.0391	3062	88.24529
3061	167.3677	3061	142.0516	3061	121.0471	3061	88.25154
3060	167.3728	3060	142.0545	3060	121.05	3060	88.25656
3059	167.3754	3059	142.0579	3059	121.0506	3059	88.26031

3058	167.3745	3058	142.063	3058	121.0512	3058	88.26369
3057	167.3722	3057	142.0694	3057	121.0529	3057	88.26753
3056	167.3721	3056	142.0771	3056	121.0561	3056	88.27211
3055	167.3764	3055	142.0877	3055	121.0609	3055	88.27759
3054	167.3834	3054	142.1015	3054	121.0669	3054	88.28401
3053	167.3892	3053	142.1142	3053	121.0721	3053	88.29048
3052	167.3905	3052	142.12	3052	121.0736	3052	88.29509
3051	167.3868	3051	142.117	3051	121.0694	3051	88.29664
3050	167.3817	3050	142.1108	3050	121.0622	3050	88.29627
3049	167.3798	3049	142.1091	3049	121.0586	3049	88.29618
3048	167.3831	3048	142.1145	3048	121.064	3048	88.2971
3047	167.39	3047	142.1228	3047	121.0778	3047	88.29781
3046	167.3974	3046	142.1287	3046	121.0939	3046	88.29738
3045	167.4039	3045	142.1323	3045	121.106	3045	88.29676
3044	167.4092	3044	142.1377	3044	121.1107	3044	88.29755
3043	167.4128	3043	142.1479	3043	121.1086	3043	88.30044
3042	167.4143	3042	142.1608	3042	121.1028	3042	88.30526
3041	167.4133	3041	142.1709	3041	121.0973	3041	88.31179
3040	167.4103	3040	142.174	3040	121.0944	3040	88.31925
3039	167.4057	3039	142.1709	3039	121.0922	3039	88.32513
3038	167.3992	3038	142.167	3038	121.0866	3038	88.3264
3037	167.3917	3037	142.1686	3037	121.0756	3037	88.32234
3036	167.3856	3036	142.1772	3036	121.062	3036	88.31608
3035	167.3838	3035	142.1887	3035	121.0506	3035	88.31234
3034	167.3877	3034	142.1979	3034	121.0439	3034	88.3139
3033	167.3955	3033	142.2041	3033	121.0423	3033	88.32017
3032	167.4037	3032	142.2099	3032	121.0455	3032	88.32866
3031	167.4098	3031	142.2167	3031	121.0535	3031	88.33694
3030	167.4144	3030	142.2227	3030	121.0646	3030	88.34338
3029	167.4196	3029	142.227	3029	121.0761	3029	88.34711
3028	167.4266	3028	142.232	3028	121.0868	3028	88.34841
3027	167.4348	3027	142.2399	3027	121.0959	3027	88.34906
3026	167.4423	3026	142.2495	3026	121.1019	3026	88.35156
3025	167.4477	3025	142.2576	3025	121.1024	3025	88.35725
3024	167.4498	3024	142.2627	3024	121.0973	3024	88.36473
3023	167.4485	3023	142.267	3023	121.0899	3023	88.37043
3022	167.4448	3022	142.2724	3022	121.0844	3022	88.37105
3021	167.4406	3021	142.2783	3021	121.0826	3021	88.36632
3020	167.4378	3020	142.2829	3020	121.083	3020	88.3601
3019	167.4376	3019	142.287	3019	121.0824	3019	88.35808
3018	167.44	3018	142.2929	3018	121.0782	3018	88.36304
3017	167.4435	3017	142.3016	3017	121.0683	3017	88.37191
3016	167.4468	3016	142.3108	3016	121.054	3016	88.37868
3015	167.45	3015	142.3177	3015	121.04	3015	88.38039
3014	167.4539	3014	142.3215	3014	121.0321	3014	88.37884

3013	167.4583	3013	142.3237	3013	121.0321	3013	88.37668
3012	167.4612	3012	142.326	3012	121.0359	3012	88.3741
3011	167.4605	3011	142.3288	3011	121.0378	3011	88.37056
3010	167.4567	3010	142.3312	3010	121.0355	3010	88.36789
3009	167.4522	3009	142.3322	3009	121.0304	3009	88.36876
3008	167.4493	3008	142.3317	3008	121.0251	3008	88.37281
3007	167.4492	3007	142.3307	3007	121.0212	3007	88.37667
3006	167.4508	3006	142.3308	3006	121.0191	3006	88.37777
3005	167.4526	3005	142.3335	3005	121.0185	3005	88.37647
3004	167.4534	3004	142.3388	3004	121.0182	3004	88.37402
3003	167.4539	3003	142.3454	3003	121.0172	3003	88.37074
3002	167.455	3002	142.3518	3002	121.0159	3002	88.36696
3001	167.4555	3001	142.3563	3001	121.0157	3001	88.3639
3000	167.453	3000	142.3573	3000	121.0163	3000	88.36193
2999	167.4469	2999	142.3537	2999	121.0154	2999	88.35923
2998	167.4394	2998	142.3465	2998	121.0099	2998	88.35394
2997	167.4341	2997	142.3387	2997	120.9998	2997	88.34696
2996	167.4321	2996	142.3335	2996	120.9885	2996	88.34088
2995	167.4328	2995	142.3317	2995	120.9796	2995	88.33623
2994	167.4345	2994	142.3313	2994	120.9743	2994	88.3304
2993	167.4354	2993	142.3299	2993	120.9706	2993	88.32058
2992	167.4336	2992	142.326	2992	120.9652	2992	88.30668
2991	167.4287	2991	142.3197	2991	120.9568	2991	88.29072
2990	167.4223	2990	142.3126	2990	120.9469	2990	88.27471
2989	167.4152	2989	142.3066	2989	120.9389	2989	88.25929
2988	167.4055	2988	142.3014	2988	120.9359	2988	88.24357
2987	167.3912	2987	142.2945	2987	120.937	2987	88.22554
2986	167.3744	2986	142.2837	2986	120.9381	2986	88.20348
2985	167.3602	2985	142.2708	2985	120.9353	2985	88.1778
2984	167.3517	2984	142.2605	2984	120.9276	2984	88.15101
2983	167.3463	2983	142.2552	2983	120.9175	2983	88.12534
2982	167.3388	2982	142.2533	2982	120.9087	2982	88.10071
2981	167.326	2981	142.2512	2981	120.9033	2981	88.0752
2980	167.3077	2980	142.2464	2980	120.9014	2980	88.04683
2979	167.2855	2979	142.2377	2979	120.9009	2979	88.01442
2978	167.2604	2978	142.2258	2978	120.8989	2978	87.97752
2977	167.2326	2977	142.2129	2977	120.8936	2977	87.93657
2976	167.2014	2976	142.2026	2976	120.8859	2976	87.89289
2975	167.1666	2975	142.1968	2975	120.8785	2975	87.84755
2974	167.1296	2974	142.1942	2974	120.873	2974	87.80007
2973	167.0918	2973	142.193	2973	120.8686	2973	87.74865
2972	167.0521	2972	142.1928	2972	120.8626	2972	87.69156
2971	167.0071	2971	142.1926	2971	120.8528	2971	87.62788
2970	166.9551	2970	142.1895	2970	120.8389	2970	87.55764
2969	166.8985	2969	142.1804	2969	120.8222	2969	87.48241

2968	166.842	2968	142.1664	2968	120.8057	2968	87.40592
2967	166.7877	2967	142.152	2967	120.7926	2967	87.33195
2966	166.7336	2966	142.1402	2966	120.7833	2966	87.26013
2965	166.6754	2965	142.1297	2965	120.7744	2965	87.18409
2964	166.6099	2964	142.1169	2964	120.7601	2964	87.09605
2963	166.5369	2963	142.1012	2963	120.7374	2963	86.994
2962	166.4592	2962	142.0864	2962	120.7083	2962	86.88412
2961	166.3801	2961	142.077	2961	120.6769	2961	86.77531
2960	166.3009	2960	142.0729	2960	120.6455	2960	86.67167
2959	166.2212	2959	142.0692	2959	120.6129	2959	86.57145
2958	166.1426	2958	142.0617	2958	120.5785	2958	86.47313
2957	166.0698	2957	142.0518	2957	120.5449	2957	86.38057
2956	166.0076	2956	142.0444	2956	120.5168	2956	86.3004
2955	165.957	2955	142.0424	2955	120.4962	2955	86.23465
2954	165.915	2954	142.0437	2954	120.48	2954	86.17757
2953	165.8773	2953	142.0447	2953	120.4632	2953	86.12054
2952	165.8393	2952	142.0446	2952	120.4431	2952	86.05904
2951	165.7956	2951	142.0441	2951	120.4205	2951	85.99429
2950	165.741	2950	142.0423	2950	120.3974	2950	85.9297
2949	165.6743	2949	142.0365	2949	120.3753	2949	85.86809
2948	165.5996	2948	142.0257	2948	120.3554	2948	85.81228
2947	165.5257	2947	142.0123	2947	120.3396	2947	85.7659
2946	165.4605	2946	141.9996	2946	120.33	2946	85.73191
2945	165.4082	2945	141.9887	2945	120.3272	2945	85.71111
2944	165.3688	2944	141.9782	2944	120.3301	2944	85.70313
2943	165.341	2943	141.9672	2943	120.3372	2943	85.70772
2942	165.3231	2942	141.9557	2942	120.3472	2942	85.72354
2941	165.3123	2941	141.9448	2941	120.3575	2941	85.74653
2940	165.3052	2940	141.9354	2940	120.3646	2940	85.77166
2939	165.3005	2939	141.9275	2939	120.3661	2939	85.79677
2938	165.2995	2938	141.9209	2938	120.3619	2938	85.8229
2937	165.3036	2937	141.9153	2937	120.3543	2937	85.85076
2936	165.3121	2936	141.9103	2936	120.3451	2936	85.87845
2935	165.3236	2935	141.9046	2935	120.3344	2935	85.90375
2934	165.3391	2934	141.8976	2934	120.3221	2934	85.9271
2933	165.361	2933	141.8904	2933	120.3086	2933	85.95075
2932	165.3874	2932	141.8851	2932	120.2932	2932	85.97506
2931	165.411	2931	141.8808	2931	120.2734	2931	85.99637
2930	165.4238	2930	141.8731	2930	120.2472	2930	86.00864
2929	165.4235	2929	141.858	2929	120.2155	2929	86.00769
2928	165.4141	2928	141.8376	2928	120.1823	2928	85.9952
2927	165.4016	2927	141.8185	2927	120.1517	2927	85.97836
2926	165.3898	2926	141.8046	2926	120.1244	2926	85.96418
2925	165.3786	2925	141.7923	2925	120.0982	2925	85.95311
2924	165.3657	2924	141.7739	2924	120.0707	2924	85.93897

2923	165.3495	2923	141.7456	2923	120.0419	2923	85.91571
2922	165.3311	2922	141.7098	2922	120.0143	2922	85.88402
2921	165.3128	2921	141.6718	2921	119.9908	2921	85.85102
2920	165.2973	2920	141.6369	2920	119.973	2920	85.82499
2919	165.2881	2919	141.61	2919	119.961	2919	85.81077
2918	165.2903	2918	141.5955	2918	119.953	2918	85.80901
2917	165.3093	2917	141.5962	2917	119.9452	2917	85.81775
2916	165.3472	2916	141.6125	2916	119.9326	2916	85.83424
2915	165.4017	2915	141.6442	2915	119.911	2915	85.85599
2914	165.4677	2914	141.6909	2914	119.8784	2914	85.88068
2913	165.5405	2913	141.7498	2913	119.8348	2913	85.9052
2912	165.6156	2912	141.8137	2912	119.7813	2912	85.92534
2911	165.6876	2911	141.8729	2911	119.7195	2911	85.93685
2910	165.7518	2910	141.9207	2910	119.6511	2910	85.9371
2909	165.8063	2909	141.9575	2909	119.5779	2909	85.92662
2908	165.8533	2908	141.9892	2908	119.5014	2908	85.90903
2907	165.8964	2907	142.0216	2907	119.4226	2907	85.88829
2906	165.9383	2906	142.055	2906	119.3413	2906	85.86457
2905	165.9787	2905	142.0852	2905	119.2566	2905	85.83303
2904	166.015	2904	142.1079	2904	119.1669	2904	85.78812
2903	166.0445	2903	142.1216	2903	119.0706	2903	85.72817
2902	166.0666	2902	142.1296	2902	118.966	2902	85.65629
2901	166.0821	2901	142.1354	2901	118.8547	2901	85.57461
2900	166.0913	2900	142.1413	2900	118.7361	2900	85.48234
2899	166.0942	2899	142.1487	2899	118.6094	2899	85.37901
2898	166.0917	2898	142.1581	2898	118.4736	2898	85.26707
2897	166.0864	2897	142.1691	2897	118.3296	2897	85.15154
2896	166.0826	2896	142.1801	2896	118.1824	2896	85.03846
2895	166.0846	2895	142.1889	2895	118.0391	2895	84.93486
2894	166.0945	2894	142.1942	2894	117.9046	2894	84.84784
2893	166.1118	2893	142.1966	2893	117.7794	2893	84.78141
2892	166.1351	2892	142.1983	2892	117.662	2892	84.73537
2891	166.1645	2891	142.202	2891	117.5539	2891	84.70843
2890	166.201	2890	142.2084	2890	117.4594	2890	84.70064
2889	166.2443	2889	142.2168	2889	117.3802	2889	84.71054
2888	166.2907	2888	142.2254	2888	117.3124	2888	84.73144
2887	166.3336	2887	142.2321	2887	117.2501	2887	84.75377
2886	166.3678	2886	142.2351	2886	117.1911	2886	84.77148
2885	166.3912	2885	142.2336	2885	117.1388	2885	84.78459
2884	166.406	2884	142.2291	2884	117.0968	2884	84.79606
2883	166.4162	2883	142.2252	2883	117.0661	2883	84.80835
2882	166.4245	2882	142.2246	2882	117.0444	2882	84.82274
2881	166.4305	2881	142.2271	2881	117.0285	2881	84.83923
2880	166.4312	2880	142.2301	2880	117.0157	2880	84.85583
2879	166.4245	2879	142.2309	2879	117.0045	2879	84.86901

2878	166.4109	2878	142.2287	2878	116.9958	2878	84.87605
2877	166.392	2877	142.2251	2877	116.9923	2877	84.877
2876	166.3685	2876	142.2229	2876	116.9971	2876	84.8745
2875	166.3389	2875	142.2232	2875	117.0132	2875	84.87205
2874	166.3004	2874	142.2253	2874	117.0412	2874	84.87175
2873	166.2511	2873	142.227	2873	117.079	2873	84.87266
2872	166.1924	2872	142.2276	2872	117.1219	2872	84.87175
2871	166.1292	2871	142.2289	2871	117.166	2871	84.86724
2870	166.0676	2870	142.2332	2870	117.209	2870	84.86095
2869	166.0108	2869	142.2408	2869	117.2499	2869	84.85655
2868	165.9587	2868	142.2497	2868	117.2858	2868	84.85607
2867	165.9105	2867	142.2579	2867	117.3127	2867	84.85915
2866	165.8668	2866	142.2644	2866	117.327	2866	84.86484
2865	165.8294	2865	142.2686	2865	117.3269	2865	84.87294
2864	165.7992	2864	142.2703	2864	117.3116	2864	84.88405
2863	165.7783	2863	142.2701	2863	117.2833	2863	84.90043
2862	165.7707	2862	142.2694	2862	117.2473	2862	84.92665
2861	165.779	2861	142.269	2861	117.2107	2861	84.96734
2860	165.801	2860	142.2681	2860	117.1802	2860	85.02373
2859	165.8299	2859	142.2651	2859	117.1614	2859	85.09369
2858	165.8604	2858	142.2588	2858	117.1603	2858	85.17511
2857	165.8906	2857	142.2485	2857	117.1832	2857	85.26752
2856	165.9194	2856	142.234	2856	117.2319	2856	85.36937
2855	165.9439	2855	142.2143	2855	117.3023	2855	85.47513
2854	165.96	2854	142.1886	2854	117.3858	2854	85.57678
2853	165.9661	2853	142.1572	2853	117.4747	2853	85.6688
2852	165.965	2852	142.1235	2852	117.566	2852	85.75242
2851	165.9652	2851	142.0954	2851	117.662	2851	85.83583
2850	165.979	2850	142.0821	2850	117.7664	2850	85.92969
2849	166.0168	2849	142.0901	2849	117.8787	2849	86.04063
2848	166.0794	2848	142.1184	2848	117.9926	2848	86.16713
2847	166.1579	2847	142.1606	2847	118.1004	2847	86.30092
2846	166.2404	2846	142.2092	2846	118.1984	2846	86.43206
2845	166.3189	2845	142.2584	2845	118.287	2845	86.55313
2844	166.3896	2844	142.3036	2844	118.368	2844	86.66025
2843	166.4504	2843	142.3408	2843	118.4426	2843	86.75268
2842	166.5005	2842	142.3692	2842	118.5127	2842	86.83223
2841	166.5419	2841	142.3918	2841	118.5798	2841	86.90202
2840	166.5777	2840	142.4132	2840	118.6429	2840	86.96399
2839	166.6099	2839	142.4353	2839	118.6984	2839	87.01786
2838	166.6385	2838	142.4557	2838	118.7436	2838	87.06295
2837	166.6635	2837	142.4714	2837	118.7803	2837	87.10089
2836	166.685	2836	142.4819	2836	118.8129	2836	87.13512
2835	166.7024	2835	142.4891	2835	118.8442	2835	87.16751
2834	166.7151	2834	142.4947	2834	118.8741	2834	87.19662

2833	166.7247	2833	142.4992	2833	118.9014	2833	87.22021
2832	166.7351	2832	142.5036	2832	118.9262	2832	87.23875
2831	166.7489	2831	142.5099	2831	118.9492	2831	87.25535
2830	166.7654	2830	142.5203	2830	118.9701	2830	87.27256
2829	166.7818	2829	142.5339	2829	118.9878	2829	87.29029
2828	166.7964	2828	142.5468	2828	119.0026	2828	87.30692
2827	166.8091	2827	142.5545	2827	119.0169	2827	87.32086
2826	166.8194	2826	142.5558	2826	119.0331	2826	87.33115
2825	166.8263	2825	142.5537	2825	119.0507	2825	87.33826
2824	166.8299	2824	142.5537	2824	119.0678	2824	87.34554
2823	166.8331	2823	142.5589	2823	119.083	2823	87.35808
2822	166.8397	2822	142.568	2822	119.0966	2822	87.37816
2821	166.8509	2821	142.577	2821	119.109	2821	87.40201
2820	166.8645	2820	142.5826	2820	119.1197	2820	87.42296
2819	166.8767	2819	142.5848	2819	119.1287	2819	87.43775
2818	166.8858	2818	142.5854	2818	119.1377	2818	87.44839
2817	166.8928	2817	142.5862	2817	119.1486	2817	87.45828
2816	166.8998	2816	142.5866	2816	119.1618	2816	87.46864
2815	166.9077	2815	142.5859	2815	119.1775	2815	87.47943
2814	166.9164	2814	142.5852	2814	119.1959	2814	87.49169
2813	166.9255	2813	142.5877	2813	119.2156	2813	87.50697
2812	166.9346	2812	142.5946	2812	119.2327	2812	87.52492
2811	166.9434	2811	142.6034	2811	119.2427	2811	87.5426
2810	166.9513	2810	142.6094	2810	119.2449	2810	87.55626
2809	166.9579	2809	142.611	2809	119.2433	2809	87.56354
2808	166.9636	2808	142.6111	2808	119.2423	2808	87.5646
2807	166.9689	2807	142.6136	2807	119.2423	2807	87.56233
2806	166.9737	2806	142.6195	2806	119.2412	2806	87.56151
2805	166.9786	2805	142.6271	2805	119.2389	2805	87.56702
2804	166.9847	2804	142.6353	2804	119.2392	2804	87.58148
2803	166.9926	2803	142.6435	2803	119.2471	2803	87.60365
2802	167	2802	142.6499	2802	119.2643	2802	87.62891
2801	167.0036	2801	142.6525	2801	119.2885	2801	87.65264
2800	167.0027	2800	142.6514	2800	119.3165	2800	87.67404
2799	167.0001	2799	142.6493	2799	119.3467	2799	87.69631
2798	166.9994	2798	142.6485	2798	119.3793	2798	87.72205
2797	167.0013	2797	142.6493	2797	119.4139	2797	87.74925
2796	167.0052	2796	142.6502	2796	119.4498	2796	87.77346
2795	167.0107	2795	142.6507	2795	119.4868	2795	87.79327
2794	167.0175	2794	142.6511	2794	119.5238	2794	87.81183
2793	167.0233	2793	142.6512	2793	119.5584	2793	87.83261
2792	167.0258	2792	142.6512	2792	119.5882	2792	87.85544
2791	167.0252	2791	142.6517	2791	119.6138	2791	87.87767
2790	167.0243	2790	142.6537	2790	119.6389	2790	87.89751
2789	167.0251	2789	142.6574	2789	119.6662	2789	87.91512

2788	167.0267	2788	142.6616	2788	119.6948	2788	87.93159
2787	167.0276	2787	142.6647	2787	119.7222	2787	87.94809
2786	167.028	2786	142.6656	2786	119.7476	2786	87.96533
2785	167.0293	2785	142.6652	2785	119.7721	2785	87.98307
2784	167.0332	2784	142.6666	2784	119.7971	2784	88.00041
2783	167.0404	2783	142.6722	2783	119.8237	2783	88.01721
2782	167.0504	2782	142.682	2782	119.8531	2782	88.03423
2781	167.061	2781	142.6925	2781	119.8838	2781	88.05145
2780	167.0684	2780	142.7004	2780	119.9118	2780	88.06728
2779	167.0704	2779	142.7047	2779	119.9333	2779	88.08033
2778	167.0679	2778	142.707	2778	119.9491	2778	88.09117
2777	167.0648	2777	142.7089	2777	119.9638	2777	88.10129
2776	167.0644	2776	142.7105	2776	119.9808	2776	88.11109
2775	167.0666	2775	142.711	2775	119.9995	2775	88.11981
2774	167.0691	2774	142.7097	2774	120.0172	2774	88.12702
2773	167.07	2773	142.7072	2773	120.0323	2773	88.13339
2772	167.0702	2772	142.7055	2772	120.0459	2772	88.14003
2771	167.0718	2771	142.7063	2771	120.0601	2771	88.14764
2770	167.0758	2770	142.7091	2770	120.0756	2770	88.15616
2769	167.08	2769	142.7122	2769	120.0908	2769	88.16458
2768	167.0816	2768	142.7136	2768	120.1033	2768	88.17163
2767	167.0801	2767	142.713	2767	120.1122	2767	88.17708
2766	167.0775	2766	142.7119	2766	120.119	2766	88.18219
2765	167.0767	2765	142.7123	2765	120.1261	2765	88.18842
2764	167.0797	2764	142.716	2764	120.1352	2764	88.19591
2763	167.0869	2763	142.7231	2763	120.147	2763	88.20334
2762	167.0968	2762	142.7309	2762	120.1611	2762	88.20899
2761	167.1054	2761	142.7354	2761	120.1754	2761	88.21181
2760	167.1084	2760	142.7348	2760	120.1868	2760	88.2123
2759	167.1049	2759	142.7308	2759	120.1945	2759	88.21272
2758	167.099	2758	142.7264	2758	120.2012	2758	88.21585
2757	167.0958	2757	142.7242	2757	120.2109	2757	88.22278
2756	167.0968	2756	142.7251	2756	120.2238	2756	88.23218
2755	167.0996	2755	142.7288	2755	120.236	2755	88.24142
2754	167.1002	2754	142.7336	2754	120.2436	2754	88.248
2753	167.097	2753	142.737	2753	120.2458	2753	88.25038
2752	167.0925	2752	142.7383	2752	120.2442	2752	88.24884
2751	167.091	2751	142.7392	2751	120.2409	2751	88.24593
2750	167.0946	2750	142.7418	2750	120.2372	2750	88.24448
2749	167.1011	2749	142.7458	2749	120.2336	2749	88.24463
2748	167.1066	2748	142.7498	2748	120.2293	2748	88.24359
2747	167.1098	2747	142.7531	2747	120.2231	2747	88.23918
2746	167.1118	2746	142.7562	2746	120.2143	2746	88.23262
2745	167.1138	2745	142.7593	2745	120.2033	2745	88.22705
2744	167.1151	2744	142.7619	2744	120.1903	2744	88.22451

2743	167.116	2743	142.7644	2743	120.1764	2743	88.22484
2742	167.1176	2742	142.7673	2742	120.1642	2742	88.22658
2741	167.1196	2741	142.7698	2741	120.1568	2741	88.22779
2740	167.12	2740	142.7701	2740	120.1548	2740	88.22699
2739	167.118	2739	142.7683	2739	120.1559	2739	88.22463
2738	167.1163	2738	142.7674	2738	120.1582	2738	88.22317
2737	167.118	2737	142.7704	2737	120.1621	2737	88.22533
2736	167.1228	2736	142.7774	2736	120.1698	2736	88.23253
2735	167.1279	2735	142.7859	2735	120.183	2735	88.24518
2734	167.1314	2734	142.793	2734	120.2018	2734	88.26269
2733	167.1337	2733	142.7966	2733	120.2244	2733	88.28238
2732	167.1356	2732	142.797	2732	120.2486	2732	88.30007
2731	167.1373	2731	142.7962	2731	120.2722	2731	88.31341
2730	167.1386	2730	142.7972	2730	120.294	2730	88.32374
2729	167.1395	2729	142.8	2729	120.3132	2729	88.33334
2728	167.1396	2728	142.8019	2728	120.328	2728	88.34217
2727	167.1391	2727	142.8002	2727	120.3369	2727	88.34868
2726	167.1394	2726	142.7958	2726	120.3405	2726	88.35261
2725	167.1424	2725	142.7917	2725	120.3423	2725	88.35467
2724	167.1474	2724	142.7909	2724	120.3459	2724	88.35472
2723	167.1529	2723	142.7935	2723	120.353	2723	88.35269
2722	167.1575	2722	142.7983	2722	120.3632	2722	88.35093
2721	167.1609	2721	142.804	2721	120.3743	2721	88.35297
2720	167.1625	2720	142.8098	2720	120.3835	2720	88.35918
2719	167.1619	2719	142.8153	2719	120.3891	2719	88.36595
2718	167.1608	2718	142.8202	2718	120.3924	2718	88.36991
2717	167.1615	2717	142.8247	2717	120.3964	2717	88.37128
2716	167.1643	2716	142.8282	2716	120.4023	2716	88.37216
2715	167.1664	2715	142.8299	2715	120.4071	2715	88.37319
2714	167.1647	2714	142.8291	2714	120.4074	2714	88.37364
2713	167.1594	2713	142.8265	2713	120.404	2713	88.37355
2712	167.154	2712	142.8247	2712	120.4013	2712	88.37426
2711	167.1519	2711	142.8263	2711	120.4026	2711	88.37698
2710	167.1544	2710	142.8316	2710	120.4071	2710	88.38173
2709	167.1597	2709	142.8371	2709	120.4118	2709	88.38746
2708	167.1648	2708	142.8384	2708	120.4151	2708	88.39254
2707	167.1676	2707	142.8343	2707	120.4184	2707	88.39535
2706	167.168	2706	142.8283	2706	120.4236	2706	88.39526
2705	167.1674	2705	142.8248	2705	120.4305	2705	88.3936
2704	167.1667	2704	142.8261	2704	120.4368	2704	88.39303
2703	167.1663	2703	142.8309	2703	120.441	2703	88.3955
2702	167.1669	2702	142.8364	2702	120.4431	2702	88.40029
2701	167.1685	2701	142.8407	2701	120.4421	2701	88.40406
2700	167.1705	2700	142.8427	2700	120.4354	2700	88.40336
2699	167.1715	2699	142.8425	2699	120.4206	2699	88.39713

2698	167.1715	2698	142.8416	2698	120.3996	2698	88.3868
2697	167.1717	2697	142.8422	2697	120.3773	2697	88.37437
2696	167.1722	2696	142.8448	2696	120.3577	2696	88.3615
2695	167.1715	2695	142.8469	2695	120.3426	2695	88.35074
2694	167.1682	2694	142.8452	2694	120.3335	2694	88.34613
2693	167.1633	2693	142.8401	2693	120.3327	2693	88.35078
2692	167.159	2692	142.8355	2692	120.3418	2692	88.36377
2691	167.1572	2691	142.8357	2691	120.3588	2691	88.38067
2690	167.1589	2690	142.8405	2690	120.3805	2690	88.39728
2689	167.1643	2689	142.8463	2689	120.4041	2689	88.41235
2688	167.1722	2688	142.8503	2688	120.4278	2688	88.42695
2687	167.1796	2687	142.8531	2687	120.4497	2687	88.4421
2686	167.1838	2686	142.8566	2686	120.4688	2686	88.45729
2685	167.1841	2685	142.8609	2685	120.4855	2685	88.4705
2684	167.1825	2684	142.8646	2684	120.5008	2684	88.47961
2683	167.1817	2683	142.8668	2683	120.5149	2683	88.48448
2682	167.1831	2682	142.8682	2682	120.5277	2682	88.48756
2681	167.1856	2681	142.8695	2681	120.54	2681	88.49182
2680	167.1876	2680	142.8698	2680	120.5523	2680	88.49786
2679	167.1877	2679	142.8675	2679	120.5635	2679	88.50368
2678	167.1866	2678	142.8629	2678	120.5719	2678	88.5076
2677	167.1856	2677	142.8585	2677	120.5774	2677	88.5106
2676	167.1866	2676	142.8573	2676	120.5825	2676	88.5152
2675	167.1901	2675	142.8601	2675	120.5894	2675	88.52244
2674	167.1952	2674	142.8646	2674	120.5979	2674	88.5305
2673	167.1997	2673	142.8676	2673	120.606	2673	88.53644
2672	167.2018	2672	142.8678	2672	120.6123	2672	88.53905
2671	167.2011	2671	142.8664	2671	120.6176	2671	88.53995
2670	167.1986	2670	142.8651	2670	120.6232	2670	88.54187
2669	167.1952	2669	142.8652	2669	120.6293	2669	88.54578
2668	167.1918	2668	142.8667	2668	120.6347	2668	88.54998
2667	167.1897	2667	142.8694	2667	120.6388	2667	88.55228
2666	167.1904	2666	142.8728	2666	120.6428	2666	88.55264
2665	167.1946	2665	142.8759	2665	120.6484	2665	88.55299
2664	167.2001	2664	142.8772	2664	120.6559	2664	88.55473
2663	167.2038	2663	142.8758	2663	120.6634	2663	88.55702
2662	167.2036	2662	142.8725	2662	120.6689	2662	88.55801
2661	167.2003	2661	142.8689	2661	120.6709	2661	88.5572
2660	167.1954	2660	142.8669	2660	120.6691	2660	88.55597
2659	167.19	2659	142.8669	2659	120.6642	2659	88.55585
2658	167.1848	2658	142.8685	2658	120.6585	2658	88.5568
2657	167.1809	2657	142.871	2657	120.6551	2657	88.55762
2656	167.1793	2656	142.8738	2656	120.6564	2656	88.55782
2655	167.1812	2655	142.8772	2655	120.6634	2655	88.55864
2654	167.1868	2654	142.8811	2654	120.675	2654	88.56169

2653	167.1947	2653	142.8858	2653	120.6884	2653	88.56703
2652	167.2028	2652	142.8911	2652	120.6999	2652	88.57316
2651	167.2092	2651	142.8959	2651	120.7067	2651	88.57867
2650	167.2136	2650	142.898	2650	120.709	2650	88.58311
2649	167.2163	2649	142.8949	2649	120.7088	2649	88.58601
2648	167.2172	2648	142.8863	2648	120.7071	2648	88.58633
2647	167.2161	2647	142.876	2647	120.7032	2647	88.58378
2646	167.2135	2646	142.8697	2646	120.6982	2646	88.58055
2645	167.2106	2645	142.8714	2645	120.6955	2645	88.58069
2644	167.2082	2644	142.88	2644	120.6973	2644	88.5872
2643	167.2067	2643	142.8901	2643	120.7023	2643	88.59925
2642	167.2071	2642	142.8958	2642	120.7074	2642	88.61173
2641	167.2101	2641	142.8944	2641	120.7112	2641	88.61802
2640	167.2146	2640	142.8872	2640	120.714	2640	88.61449
2639	167.2178	2639	142.8786	2639	120.7152	2639	88.6037
2638	167.2176	2638	142.874	2638	120.714	2638	88.59306
2637	167.2155	2637	142.8769	2637	120.7122	2637	88.58951
2636	167.2158	2636	142.8867	2636	120.713	2636	88.59505
2635	167.2211	2635	142.8987	2635	120.7183	2635	88.60679
2634	167.2304	2634	142.9085	2634	120.7267	2634	88.62004
2633	167.2397	2633	142.9149	2633	120.7355	2633	88.63117
2632	167.246	2632	142.9197	2632	120.7434	2632	88.63867
2631	167.2489	2631	142.9243	2631	120.7494	2631	88.64316
2630	167.2503	2630	142.9277	2630	120.753	2630	88.64643
2629	167.2516	2629	142.9296	2629	120.7541	2629	88.64991
2628	167.2526	2628	142.9307	2628	120.7528	2628	88.65369
2627	167.2527	2627	142.9309	2627	120.7491	2627	88.65675
2626	167.2521	2626	142.9277	2626	120.7434	2626	88.65757
2625	167.2519	2625	142.9199	2625	120.7376	2625	88.65464
2624	167.2534	2624	142.9115	2624	120.734	2624	88.64775
2623	167.2565	2623	142.9089	2623	120.7338	2623	88.63986
2622	167.2609	2622	142.9153	2622	120.7368	2622	88.63644
2621	167.2666	2621	142.9277	2621	120.7424	2621	88.64117
2620	167.273	2620	142.9404	2620	120.75	2620	88.65171
2619	167.2774	2619	142.949	2619	120.7572	2619	88.66081
2618	167.2764	2618	142.9514	2618	120.7598	2618	88.6624
2617	167.2697	2617	142.9489	2617	120.7561	2617	88.65694
2616	167.2612	2616	142.9455	2616	120.7492	2616	88.65088
2615	167.2562	2615	142.9448	2615	120.7449	2615	88.6503
2614	167.2566	2614	142.946	2614	120.7462	2614	88.65461
2613	167.2602	2613	142.9443	2613	120.7507	2613	88.65689
2612	167.2631	2612	142.9378	2612	120.7537	2612	88.65128
2611	167.2631	2611	142.9301	2611	120.7524	2611	88.63969
2610	167.2598	2610	142.9258	2610	120.7474	2610	88.63003
2609	167.2544	2609	142.925	2609	120.7415	2609	88.62817

2608	167.2493	2608	142.9252	2608	120.7379	2608	88.63351
2607	167.2471	2607	142.9252	2607	120.7394	2607	88.64175
2606	167.2485	2606	142.9256	2606	120.7465	2606	88.64924
2605	167.2515	2605	142.9263	2605	120.7555	2605	88.65368
2604	167.2542	2604	142.9264	2604	120.76	2604	88.6538
2603	167.257	2603	142.9263	2603	120.7567	2603	88.65089
2602	167.2616	2602	142.928	2602	120.7499	2602	88.64906
2601	167.2672	2601	142.9331	2601	120.7489	2601	88.65164
2600	167.2717	2600	142.9428	2600	120.7585	2600	88.65805
2599	167.2753	2599	142.9571	2599	120.7755	2599	88.66623
2598	167.2813	2598	142.9738	2598	120.7929	2598	88.67621
2597	167.2911	2597	142.9883	2597	120.8057	2597	88.68868
2596	167.3006	2596	142.9968	2596	120.8119	2596	88.70087
2595	167.3033	2595	142.9988	2595	120.8098	2595	88.70754
2594	167.2964	2594	142.9955	2594	120.8003	2594	88.70688
2593	167.2836	2593	142.9875	2593	120.789	2593	88.70302
2592	167.272	2592	142.9754	2592	120.7835	2592	88.70125
2591	167.2672	2591	142.962	2591	120.786	2591	88.7021
2590	167.2693	2590	142.951	2590	120.7917	2590	88.70152
2589	167.2739	2589	142.9446	2589	120.7945	2589	88.69647
2588	167.2764	2588	142.9427	2588	120.7934	2588	88.68903
2587	167.276	2587	142.9453	2587	120.7911	2587	88.68487
2586	167.2756	2586	142.9526	2586	120.7891	2586	88.68788
2585	167.2779	2585	142.9627	2585	120.7872	2585	88.69675
2584	167.2834	2584	142.9717	2584	120.785	2584	88.70647
2583	167.2899	2583	142.9767	2583	120.7837	2583	88.71248
2582	167.2942	2582	142.978	2582	120.7841	2582	88.71325
2581	167.2946	2581	142.9782	2581	120.7859	2581	88.71027
2580	167.2921	2580	142.9814	2580	120.7887	2580	88.70722
2579	167.2902	2579	142.9902	2579	120.793	2579	88.70787
2578	167.291	2578	143.0024	2578	120.7989	2578	88.71236
2577	167.2926	2577	143.0103	2577	120.805	2577	88.71596
2576	167.2921	2576	143.0071	2576	120.8096	2576	88.714
2575	167.2903	2575	142.9932	2575	120.8132	2575	88.70794
2574	167.2906	2574	142.976	2574	120.817	2574	88.70366
2573	167.2939	2573	142.9622	2573	120.8198	2573	88.70391
2572	167.2976	2572	142.953	2572	120.8173	2572	88.70616
2571	167.2993	2571	142.948	2571	120.8065	2571	88.70896
2570	167.2993	2570	142.9481	2570	120.792	2570	88.71579
2569	167.2991	2569	142.9552	2569	120.7835	2569	88.72952
2568	167.3001	2568	142.9684	2568	120.7879	2568	88.74638
2567	167.3032	2567	142.9835	2567	120.803	2567	88.75908
2566	167.3077	2566	142.9955	2566	120.8195	2566	88.76409
2565	167.3095	2565	143.0006	2565	120.8286	2565	88.76271
2564	167.3044	2564	142.9976	2564	120.827	2564	88.7572

2563	167.2949	2563	142.9886	2563	120.8179	2563	88.74984
2562	167.2902	2562	142.9778	2562	120.8084	2562	88.74458
2561	167.2968	2561	142.9705	2561	120.8067	2561	88.74515
2560	167.3106	2560	142.971	2560	120.8168	2560	88.75061
2559	167.3209	2559	142.9793	2559	120.8346	2559	88.75525
2558	167.3201	2558	142.9892	2558	120.8495	2558	88.75375
2557	167.3094	2557	142.9922	2557	120.8523	2557	88.74566
2556	167.2963	2556	142.9855	2556	120.8424	2556	88.73563
2555	167.2887	2555	142.9761	2555	120.8282	2555	88.73077
2554	167.289	2554	142.9742	2554	120.8204	2554	88.73603
2553	167.2943	2553	142.9831	2553	120.8245	2553	88.74934
2552	167.2996	2552	142.997	2552	120.8371	2552	88.76176
2551	167.3039	2551	143.0089	2551	120.8495	2551	88.76508
2550	167.3088	2550	143.0164	2550	120.8541	2550	88.7595
2549	167.3136	2549	143.0206	2549	120.8507	2549	88.75257
2548	167.3146	2548	143.0224	2548	120.8457	2548	88.75147
2547	167.312	2547	143.0236	2547	120.8465	2547	88.75808
2546	167.3115	2546	143.0261	2546	120.8551	2546	88.76983
2545	167.3178	2545	143.031	2545	120.8682	2545	88.78195
2544	167.3273	2544	143.0365	2544	120.8792	2544	88.78943
2543	167.3326	2543	143.0401	2543	120.8827	2543	88.79017
2542	167.3303	2542	143.0396	2542	120.8768	2542	88.78714
2541	167.3228	2541	143.0332	2541	120.8643	2541	88.78534
2540	167.3137	2540	143.0209	2540	120.8518	2540	88.78587
2539	167.3061	2539	143.0065	2539	120.845	2539	88.7847
2538	167.3016	2538	142.9973	2538	120.8448	2538	88.77794
2537	167.3006	2537	142.9976	2537	120.8478	2537	88.7672
2536	167.3019	2536	143.0054	2536	120.8502	2536	88.759
2535	167.3047	2535	143.0154	2535	120.8515	2535	88.75927
2534	167.3115	2534	143.0244	2534	120.8536	2534	88.76766
2533	167.3241	2533	143.0312	2533	120.8577	2533	88.77683
2532	167.3387	2532	143.0344	2532	120.862	2532	88.77864
2531	167.3476	2531	143.0333	2531	120.8631	2531	88.77203
2530	167.3457	2530	143.0299	2530	120.8593	2530	88.76372
2529	167.3348	2529	143.028	2529	120.852	2529	88.76043
2528	167.3207	2528	143.0287	2528	120.845	2528	88.76261
2527	167.3098	2527	143.0302	2527	120.8417	2527	88.76731
2526	167.3069	2526	143.0308	2526	120.8438	2526	88.77377
2525	167.3145	2525	143.0311	2525	120.85	2525	88.78247
2524	167.3298	2524	143.0325	2524	120.8564	2524	88.79065
2523	167.344	2523	143.0334	2523	120.8586	2523	88.79383
2522	167.347	2522	143.0305	2522	120.8542	2522	88.79199
2521	167.3361	2521	143.0214	2521	120.8452	2521	88.79027
2520	167.3189	2520	143.0081	2520	120.8366	2520	88.79226
2519	167.3088	2519	142.995	2519	120.8326	2519	88.79531

2518	167.3128	2518	142.9869	2518	120.8331	2518	88.79406
2517	167.3259	2517	142.9857	2517	120.834	2517	88.78653
2516	167.3362	2516	142.9892	2516	120.8317	2516	88.77619
2515	167.3369	2515	142.9931	2515	120.8271	2515	88.76943
2514	167.3307	2514	142.9943	2514	120.8245	2514	88.77179
2513	167.3247	2513	142.9937	2513	120.8275	2513	88.78423
2512	167.3232	2512	142.9954	2512	120.8347	2512	88.80184
2511	167.3259	2511	143.0028	2511	120.8412	2511	88.81701
2510	167.3302	2510	143.0151	2510	120.844	2510	88.82455
2509	167.3332	2509	143.0267	2509	120.8447	2509	88.82374
2508	167.334	2508	143.0311	2508	120.8472	2508	88.81661
2507	167.3348	2507	143.027	2507	120.8531	2507	88.80668
2506	167.3388	2506	143.0208	2506	120.8603	2506	88.79925
2505	167.3455	2505	143.0207	2505	120.8659	2505	88.79926
2504	167.3505	2504	143.0288	2504	120.8683	2504	88.80658
2503	167.3507	2503	143.0377	2503	120.8672	2503	88.81475
2502	167.3481	2502	143.0378	2502	120.8627	2502	88.81657
2501	167.3472	2501	143.0279	2501	120.8567	2501	88.8113
2500	167.3508	2500	143.0175	2500	120.854	2500	88.80574
2499	167.3579	2499	143.0171	2499	120.8574	2499	88.80767
2498	167.3653	2498	143.0272	2498	120.8642	2498	88.81894
2497	167.3695	2497	143.0388	2497	120.8683	2497	88.83452
2496	167.3696	2496	143.0435	2496	120.8674	2496	88.84772
2495	167.3692	2495	143.0415	2495	120.8661	2495	88.85598
2494	167.3723	2494	143.0397	2494	120.87	2494	88.86088
2493	167.3778	2493	143.0421	2493	120.8785	2493	88.86368
2492	167.3795	2492	143.0457	2492	120.8867	2492	88.86321
2491	167.3749	2491	143.0444	2491	120.891	2491	88.85913
2490	167.3696	2490	143.0374	2490	120.8922	2490	88.85537
2489	167.3708	2489	143.0321	2489	120.8935	2489	88.8577
2488	167.3789	2488	143.0378	2488	120.8965	2488	88.86759
2487	167.3873	2487	143.0557	2487	120.9006	2487	88.88021
2486	167.3898	2486	143.0758	2486	120.9033	2486	88.88869
2485	167.3852	2485	143.0851	2485	120.9022	2485	88.8896
2484	167.3752	2484	143.0789	2484	120.8979	2484	88.88443
2483	167.3633	2483	143.0642	2483	120.8951	2483	88.87706
2482	167.3537	2482	143.051	2482	120.8985	2482	88.87058
2481	167.3504	2481	143.0438	2481	120.9074	2481	88.86599
2480	167.3541	2480	143.04	2480	120.9156	2480	88.86313
2479	167.362	2479	143.0353	2479	120.9187	2479	88.86267
2478	167.3706	2478	143.0302	2478	120.9178	2478	88.86658
2477	167.3783	2477	143.0304	2477	120.9172	2477	88.87578
2476	167.3844	2476	143.0408	2476	120.9176	2476	88.88716
2475	167.3872	2475	143.0586	2475	120.9152	2475	88.89373
2474	167.3841	2474	143.0719	2474	120.9061	2474	88.88909

2473	167.3748	2473	143.0701	2473	120.8915	2473	88.87322
2472	167.3637	2472	143.0539	2472	120.8783	2472	88.85428
2471	167.3575	2471	143.0353	2471	120.8734	2471	88.84322
2470	167.3586	2470	143.026	2470	120.878	2470	88.84508
2469	167.3642	2469	143.0288	2469	120.8873	2469	88.85533
2468	167.37	2468	143.0397	2468	120.8944	2468	88.86513
2467	167.3755	2467	143.0544	2467	120.8957	2467	88.86946
2466	167.3814	2466	143.0698	2466	120.8916	2466	88.86906
2465	167.385	2465	143.0823	2465	120.8848	2465	88.86617
2464	167.382	2464	143.0885	2464	120.8785	2464	88.86172
2463	167.3732	2463	143.0869	2463	120.8769	2463	88.85652
2462	167.3639	2462	143.0787	2462	120.8819	2462	88.85249
2461	167.3584	2461	143.0681	2461	120.8914	2461	88.851
2460	167.3575	2460	143.0621	2460	120.8998	2460	88.85188
2459	167.3603	2459	143.0659	2459	120.9031	2459	88.85499
2458	167.3665	2458	143.0761	2458	120.9014	2458	88.86082
2457	167.3738	2457	143.0824	2457	120.8963	2457	88.86879
2456	167.3783	2456	143.0784	2456	120.8896	2456	88.87677
2455	167.3787	2455	143.0688	2455	120.8844	2455	88.88377
2454	167.3769	2454	143.0636	2454	120.8856	2454	88.89101
2453	167.3739	2453	143.0649	2453	120.8949	2453	88.89865
2452	167.3685	2452	143.0661	2452	120.9074	2452	88.90319
2451	167.3615	2451	143.0623	2451	120.9142	2451	88.90026
2450	167.3573	2450	143.0565	2450	120.9119	2450	88.88977
2449	167.3595	2449	143.0544	2449	120.9055	2449	88.8772
2448	167.3677	2448	143.057	2448	120.9027	2448	88.87023
2447	167.38	2447	143.0604	2447	120.9061	2447	88.87391
2446	167.3947	2446	143.0619	2446	120.9119	2446	88.88729
2445	167.4074	2445	143.0626	2445	120.916	2445	88.90325
2444	167.4114	2444	143.065	2444	120.9189	2444	88.91313
2443	167.4049	2443	143.0709	2443	120.924	2443	88.91332
2442	167.3948	2442	143.0794	2442	120.9325	2442	88.90703
2441	167.389	2441	143.0886	2441	120.942	2441	88.89985
2440	167.3885	2440	143.0973	2440	120.9494	2440	88.89539
2439	167.3884	2439	143.105	2439	120.9528	2439	88.89518
2438	167.3849	2438	143.1095	2438	120.951	2438	88.89961
2437	167.3775	2437	143.1077	2437	120.943	2437	88.90659
2436	167.3667	2436	143.0994	2436	120.9295	2436	88.91108
2435	167.3539	2435	143.0888	2435	120.9148	2435	88.90876
2434	167.3435	2434	143.0806	2434	120.904	2434	88.90014
2433	167.3406	2433	143.0762	2433	120.8993	2433	88.89028
2432	167.3468	2432	143.0761	2432	120.9015	2432	88.88517
2431	167.3585	2431	143.0821	2431	120.9104	2431	88.88833
2430	167.37	2430	143.0948	2430	120.9237	2430	88.89855
2429	167.3759	2429	143.1083	2429	120.9356	2429	88.90951

2428	167.3746	2428	143.1139	2428	120.9407	2428	88.91371
2427	167.3694	2427	143.1083	2427	120.9387	2427	88.90899
2426	167.3661	2426	143.0968	2426	120.9339	2426	88.90052
2425	167.3679	2425	143.0869	2425	120.9299	2425	88.89533
2424	167.3734	2424	143.0818	2424	120.9273	2424	88.89574
2423	167.3798	2423	143.0807	2423	120.9257	2423	88.89905
2422	167.385	2422	143.0822	2422	120.9237	2422	88.90179
2421	167.386	2421	143.0851	2421	120.9192	2421	88.90263
2420	167.3798	2420	143.0884	2420	120.911	2420	88.90228
2419	167.3679	2419	143.0922	2419	120.9014	2419	88.90243
2418	167.3577	2418	143.0973	2418	120.8961	2418	88.90425
2417	167.3559	2417	143.1037	2417	120.8986	2417	88.90662
2416	167.3613	2416	143.1094	2416	120.9089	2416	88.90664
2415	167.3662	2415	143.1112	2415	120.9228	2415	88.90239
2414	167.3648	2414	143.107	2414	120.934	2414	88.89503
2413	167.3574	2413	143.0972	2413	120.9367	2413	88.88785
2412	167.3485	2412	143.0854	2412	120.9303	2412	88.88445
2411	167.3427	2411	143.0774	2411	120.921	2411	88.8872
2410	167.3418	2410	143.0765	2410	120.9151	2410	88.89506
2409	167.3446	2409	143.0798	2409	120.9123	2409	88.90223
2408	167.3484	2408	143.0813	2408	120.9087	2408	88.90182
2407	167.3513	2407	143.078	2407	120.905	2407	88.89323
2406	167.3539	2406	143.073	2406	120.9068	2406	88.88438
2405	167.3576	2405	143.0712	2405	120.9164	2405	88.88454
2404	167.3628	2404	143.0751	2404	120.929	2404	88.89523
2403	167.3692	2403	143.0824	2403	120.9375	2403	88.90918
2402	167.3744	2402	143.0883	2402	120.9381	2402	88.91757
2401	167.3758	2401	143.0901	2401	120.9319	2401	88.91791
2400	167.374	2400	143.0908	2400	120.924	2400	88.91576
2399	167.3755	2399	143.0973	2399	120.922	2399	88.91921
2398	167.3859	2398	143.1116	2398	120.9301	2398	88.93067
2397	167.4025	2397	143.1268	2397	120.9423	2397	88.94418
2396	167.4144	2396	143.1323	2396	120.9472	2396	88.95186
2395	167.4141	2395	143.1244	2395	120.9393	2395	88.95155
2394	167.4043	2394	143.1075	2394	120.9234	2394	88.94619
2393	167.3936	2393	143.0883	2393	120.9067	2393	88.93763
2392	167.3878	2392	143.071	2392	120.892	2392	88.92573
2391	167.3852	2391	143.0576	2391	120.879	2391	88.91322
2390	167.3799	2390	143.0481	2390	120.8662	2390	88.90559
2389	167.3663	2389	143.0393	2389	120.8495	2389	88.90382
2388	167.3439	2388	143.0266	2388	120.8221	2388	88.90118
2387	167.3177	2387	143.0089	2387	120.7815	2387	88.89062
2386	167.2936	2386	142.9886	2386	120.7345	2386	88.87312
2385	167.2745	2385	142.9689	2385	120.6945	2385	88.8564
2384	167.2627	2384	142.9514	2384	120.6732	2384	88.84739

2383	167.2644	2383	142.9388	2383	120.6747	2383	88.84867
2382	167.2873	2382	142.9357	2382	120.6964	2382	88.86079
2381	167.3296	2381	142.9438	2381	120.7311	2381	88.8829
2380	167.3753	2380	142.9557	2380	120.7663	2380	88.9082
2379	167.4035	2379	142.9594	2379	120.7867	2379	88.92342
2378	167.4073	2378	142.9505	2378	120.7865	2378	88.91912
2377	167.3958	2377	142.9368	2377	120.7749	2377	88.89845
2376	167.3801	2376	142.9258	2376	120.7635	2376	88.87203
2375	167.3636	2375	142.9159	2375	120.7546	2375	88.84923
2374	167.3504	2374	142.9071	2374	120.7542	2374	88.84056
2373	167.3525	2373	142.9086	2373	120.7799	2373	88.85782
2372	167.3754	2372	142.9244	2372	120.8308	2372	88.89615
2371	167.4091	2371	142.9443	2371	120.8738	2371	88.92768
2370	167.4467	2370	142.968	2370	120.8958	2370	88.936
2369	167.4908	2369	143.0121	2369	120.9275	2369	88.94069
2368	167.5193	2368	143.0617	2368	120.9509	2368	88.95424
2367	167.4798	2367	143.056	2367	120.871	2367	88.94238
2366	167.3642	2366	142.9773	2366	120.6917	2366	88.87919
2365	167.2513	2365	142.8991	2365	120.5805	2365	88.80845
2364	167.2196	2364	142.8672	2364	120.6056	2364	88.78078
2363	167.2594	2363	142.817	2363	120.6033	2363	88.78026
2362	167.2999	2362	142.6742	2362	120.4535	2362	88.75528
2361	167.3042	2361	142.5051	2361	120.3073	2361	88.71703
2360	167.256	2360	142.4154	2360	120.289	2360	88.69683
2359	167.164	2359	142.3998	2359	120.2648	2359	88.68271
2358	167.1084	2358	142.4264	2358	120.237	2358	88.68339
2357	167.1613	2357	142.5084	2357	120.35	2357	88.72678
2356	167.2734	2356	142.6208	2356	120.5917	2356	88.77648
2355	167.3415	2355	142.6949	2355	120.7493	2355	88.77473
2354	167.353	2354	142.7098	2354	120.7598	2354	88.73502
2353	167.3371	2353	142.6822	2353	120.6499	2353	88.68777
2352	167.3164	2352	142.6591	2352	120.5383	2352	88.65897
2351	167.3055	2351	142.6764	2351	120.5282	2351	88.66816
2350	167.2889	2350	142.7013	2350	120.5572	2350	88.68861
2349	167.2375	2349	142.6733	2349	120.4859	2349	88.66939
2348	167.1466	2348	142.5748	2348	120.2385	2348	88.5923
2347	167.0616	2347	142.4577	2347	119.8911	2347	88.50021
2346	167.0387	2346	142.4023	2346	119.6471	2346	88.46187
2345	167.0781	2345	142.4366	2345	119.6764	2345	88.49875
2344	167.1157	2344	142.505	2344	119.9244	2344	88.55904
2343	167.1064	2343	142.5423	2343	120.1653	2343	88.583
2342	167.0663	2342	142.5254	2342	120.2505	2342	88.54758
2341	167.0206	2341	142.4721	2341	120.165	2341	88.47088
2340	166.9787	2340	142.4266	2340	120.0164	2340	88.40624
2339	166.9436	2339	142.4107	2339	119.9083	2339	88.38826

2338	166.9432	2338	142.4326	2338	119.915	2338	88.42276
2337	166.9899	2337	142.4813	2337	120.0309	2337	88.48132
2336	167.0405	2336	142.5066	2336	120.1111	2336	88.50531
2335	167.0368	2335	142.4603	2335	120.031	2335	88.45806
2334	166.986	2334	142.365	2334	119.8342	2334	88.37526
2333	166.9313	2333	142.2819	2333	119.6703	2333	88.31275
2332	166.9018	2332	142.2553	2332	119.5931	2332	88.29427
2331	166.9142	2331	142.3073	2331	119.6427	2331	88.3305
2330	166.9799	2330	142.4362	2330	119.8286	2330	88.43089
2329	167.0715	2329	142.5846	2329	120.1156	2329	88.55931
2328	167.1092	2328	142.6476	2328	120.2882	2328	88.62236
2327	167.0478	2327	142.5701	2327	120.2097	2327	88.56173
2326	166.925	2326	142.3945	2326	119.914	2326	88.41103
2325	166.8123	2325	142.2066	2325	119.5729	2325	88.24769
2324	166.7498	2324	142.0695	2324	119.3071	2324	88.1288
2323	166.7304	2323	142.0079	2323	119.1829	2323	88.0754
2322	166.7431	2322	142.0299	2322	119.2204	2322	88.09427
2321	166.7777	2321	142.1199	2321	119.4061	2321	88.169
2320	166.8194	2320	142.236	2320	119.6371	2320	88.25825
2319	166.8579	2319	142.34	2319	119.8426	2319	88.33017
2318	166.8967	2318	142.4208	2318	119.9894	2318	88.38421
2317	166.9401	2317	142.4837	2317	120.1044	2317	88.4325
2316	166.9754	2316	142.5274	2316	120.1638	2316	88.4722
2315	166.9901	2315	142.5496	2315	120.1708	2315	88.49566
2314	166.986	2314	142.5571	2314	120.1413	2314	88.50483
2313	166.9774	2313	142.5658	2313	120.1351	2313	88.51031
2312	166.9753	2312	142.5856	2312	120.1666	2312	88.51778
2311	166.9869	2311	142.6178	2311	120.2328	2311	88.53193
2310	167.0157	2310	142.6571	2310	120.3093	2310	88.56025
2309	167.0559	2309	142.6926	2309	120.3845	2309	88.603
2308	167.092	2308	142.7141	2308	120.4373	2308	88.64715
2307	167.1165	2307	142.7234	2307	120.4674	2307	88.68131
2306	167.1357	2306	142.7338	2306	120.4938	2306	88.70899
2305	167.1528	2305	142.7529	2305	120.5338	2305	88.73765
2304	167.1625	2304	142.7798	2304	120.5849	2304	88.76947
2303	167.1612	2303	142.8111	2303	120.6349	2303	88.80061
2302	167.16	2302	142.8475	2302	120.6728	2302	88.82894
2301	167.1699	2301	142.8864	2301	120.6949	2301	88.852
2300	167.1872	2300	142.9184	2300	120.6932	2300	88.86648
2299	167.1995	2299	142.9347	2299	120.6782	2299	88.87309
2298	167.2039	2298	142.9379	2298	120.6671	2298	88.87999
2297	167.2108	2297	142.9413	2297	120.6884	2297	88.89694
2296	167.2261	2296	142.9542	2296	120.7369	2296	88.92291
2295	167.2442	2295	142.9712	2295	120.7916	2295	88.94693
2294	167.2538	2294	142.9771	2294	120.8227	2294	88.95677

2293	167.2505	2293	142.9648	2293	120.8263	2293	88.94886
2292	167.239	2292	142.945	2292	120.814	2292	88.93002
2291	167.2331	2291	142.9394	2291	120.8082	2291	88.91683
2290	167.2472	2290	142.9603	2290	120.8231	2290	88.9258
2289	167.2811	2289	142.9986	2289	120.8539	2289	88.95777
2288	167.3164	2288	143.0327	2288	120.8808	2288	88.99337
2287	167.3342	2287	143.0494	2287	120.8868	2287	89.01053
2286	167.335	2286	143.0518	2286	120.876	2286	89.00516
2285	167.3322	2285	143.0498	2285	120.8613	2285	88.9901
2284	167.3336	2284	143.0488	2284	120.8553	2284	88.97904
2283	167.3368	2283	143.049	2283	120.8626	2283	88.97635
2282	167.3444	2282	143.0535	2282	120.8912	2282	88.98312
2281	167.367	2281	143.0693	2281	120.9433	2281	89.00134
2280	167.4068	2280	143.0984	2280	121.0043	2280	89.02822
2279	167.4456	2279	143.129	2279	121.0431	2279	89.05078
2278	167.4633	2278	143.144	2278	121.0435	2278	89.05649
2277	167.4586	2277	143.1379	2277	121.0206	2277	89.04484
2276	167.4448	2276	143.1207	2276	120.998	2276	89.02375
2275	167.4309	2275	143.1061	2275	120.9852	2275	88.99977
2274	167.4178	2274	143.1004	2274	120.9814	2274	88.97816
2273	167.4084	2273	143.1025	2273	120.9919	2273	88.96721
2272	167.4071	2272	143.1081	2272	121.0128	2272	88.97234
2271	167.4118	2271	143.1128	2271	121.025	2271	88.98863
2270	167.4124	2270	143.1137	2270	121.0141	2270	89.00485
2269	167.4017	2269	143.1092	2269	120.9938	2269	89.01375
2268	167.3818	2268	143.0992	2268	120.9851	2268	89.01471
2267	167.3618	2267	143.0848	2267	120.9875	2267	89.01022
2266	167.3509	2266	143.0701	2266	120.9836	2266	89.00453
2265	167.3532	2265	143.0627	2265	120.9712	2265	89.00442
2264	167.3654	2264	143.0681	2264	120.9659	2264	89.01426
2263	167.3788	2263	143.084	2263	120.9765	2263	89.03059
2262	167.3872	2262	143.1033	2262	120.9949	2262	89.04531
2261	167.3941	2261	143.1216	2261	121.0108	2261	89.05503
2260	167.4106	2260	143.1416	2260	121.0268	2260	89.06398
2259	167.4406	2259	143.1646	2259	121.0455	2259	89.07462
2258	167.4707	2258	143.1826	2258	121.0603	2258	89.08025
2257	167.4777	2257	143.1827	2257	121.0591	2257	89.07211
2256	167.4559	2256	143.1629	2256	121.0424	2256	89.0555
2255	167.427	2255	143.1378	2255	121.026	2255	89.0482
2254	167.4166	2254	143.1251	2254	121.024	2254	89.05785
2253	167.4255	2253	143.1292	2253	121.0345	2253	89.06832
2252	167.437	2252	143.1392	2252	121.0427	2252	89.05918
2251	167.4456	2251	143.1417	2251	121.0427	2251	89.03528
2250	167.4611	2250	143.1335	2250	121.0409	2250	89.02538
2249	167.4829	2249	143.1232	2249	121.0463	2249	89.052

2248	167.4946	2248	143.1238	2248	121.0544	2248	89.11146
2247	167.4889	2247	143.1403	2247	121.0588	2247	89.18138
2246	167.4777	2246	143.16	2246	121.058	2246	89.23376
2245	167.4659	2245	143.1629	2245	121.0517	2245	89.24376
2244	167.4355	2244	143.1417	2244	121.0342	2244	89.20554
2243	167.3744	2243	143.1086	2243	121.0072	2243	89.14595
2242	167.3039	2242	143.0781	2242	120.9876	2242	89.10519
2241	167.2581	2241	143.0525	2241	120.9877	2241	89.09368
2240	167.2459	2240	143.0287	2240	120.9934	2240	89.0821
2239	167.2549	2239	143.011	2239	120.9831	2239	89.04539
2238	167.2812	2238	143.0026	2238	120.964	2238	89.0004
2237	167.326	2237	142.9901	2237	120.9624	2237	88.98259
2236	167.3696	2236	142.9542	2236	120.9785	2236	88.99982
2235	167.3807	2235	142.9017	2235	120.9846	2235	89.03236
2234	167.3578	2234	142.8704	2234	120.9778	2234	89.06874
2233	167.3361	2233	142.8903	2233	120.9972	2233	89.11311
2232	167.3455	2232	142.9509	2232	121.0679	2232	89.16041
2231	167.3811	2231	143.0181	2231	121.1561	2231	89.19399
2230	167.4241	2230	143.0749	2230	121.2089	2230	89.21532
2229	167.471	2229	143.1289	2229	121.2205	2229	89.25134
2228	167.5262	2228	143.1841	2228	121.2261	2228	89.3168
2227	167.5816	2227	143.2263	2227	121.2462	2227	89.38498
2226	167.621	2226	143.2422	2226	121.269	2226	89.4124
2225	167.6436	2225	143.2407	2225	121.286	2225	89.38865
2224	167.6668	2224	143.2459	2224	121.3095	2224	89.35115
2223	167.7028	2223	143.27	2223	121.3481	2223	89.34928
2222	167.7394	2222	143.3007	2222	121.3859	2222	89.39833
2221	167.7487	2221	143.3129	2221	121.3946	2221	89.46581
2220	167.7152	2220	143.2942	2220	121.3593	2220	89.50121
2219	167.6515	2219	143.2572	2219	121.2903	2219	89.48224
2218	167.5826	2218	143.2277	2218	121.2201	2218	89.42943
2217	167.5193	2217	143.2153	2217	121.184	2217	89.37687
2216	167.458	2216	143.2008	2216	121.1898	2216	89.33844
2215	167.4059	2215	143.1648	2215	121.2138	2215	89.31099
2214	167.3915	2214	143.124	2214	121.2339	2214	89.29916
2213	167.4311	2213	143.1216	2213	121.2523	2213	89.31423
2212	167.4949	2212	143.1703	2212	121.2761	2212	89.34923
2211	167.5271	2211	143.2261	2211	121.2926	2211	89.37875
2210	167.5028	2210	143.2372	2210	121.2905	2210	89.39055
2209	167.4506	2209	143.207	2209	121.2881	2209	89.39878
2208	167.4176	2208	143.1858	2208	121.3127	2208	89.41545
2207	167.4206	2207	143.2007	2207	121.3553	2207	89.42429
2206	167.4373	2206	143.2214	2206	121.3759	2206	89.39863
2205	167.4343	2205	143.201	2205	121.353	2205	89.33773
2204	167.4014	2204	143.1327	2204	121.3065	2204	89.27008

2203	167.3615	2203	143.0533	2203	121.2703	2203	89.2218
2202	167.3499	2202	143.0039	2202	121.2607	2202	89.1954
2201	167.3843	2201	143.0031	2201	121.2753	2201	89.18035
2200	167.4563	2200	143.0451	2200	121.3011	2200	89.17363
2199	167.5468	2199	143.1092	2199	121.3219	2199	89.18353
2198	167.6361	2198	143.1704	2198	121.332	2198	89.21332
2197	167.6981	2197	143.2106	2197	121.3398	2197	89.24544
2196	167.7048	2196	143.2252	2196	121.3471	2196	89.25045
2195	167.652	2195	143.2154	2195	121.3325	2195	89.21585
2194	167.5677	2194	143.1824	2194	121.2781	2194	89.16019
2193	167.4875	2193	143.1355	2193	121.2059	2193	89.11404
2192	167.4233	2192	143.0971	2192	121.1642	2192	89.09177
2191	167.3639	2191	143.0851	2191	121.1735	2191	89.08889
2190	167.3002	2190	143.091	2190	121.2098	2190	89.10048
2189	167.2436	2189	143.0852	2189	121.2402	2189	89.12807
2188	167.2251	2188	143.0484	2188	121.2562	2188	89.17144
2187	167.2791	2187	142.995	2187	121.272	2187	89.2254
2186	167.413	2186	142.9655	2186	121.3059	2186	89.28115
2185	167.5831	2185	142.9995	2185	121.3677	2185	89.32225
2184	167.7208	2184	143.1028	2184	121.4459	2184	89.32868
2183	167.7926	2183	143.2362	2183	121.5083	2183	89.29736
2182	167.8113	2182	143.3351	2182	121.527	2182	89.25081
2181	167.7893	2181	143.3535	2181	121.5047	2181	89.21092
2180	167.7205	2180	143.2988	2180	121.4655	2180	89.17025
2179	167.6165	2179	143.2161	2179	121.4206	2179	89.10997
2178	167.5225	2178	143.1443	2178	121.3679	2178	89.04152
2177	167.4739	2177	143.0974	2177	121.3175	2177	89.00703
2176	167.4579	2176	143.0726	2176	121.2921	2176	89.03251
2175	167.446	2175	143.0632	2175	121.2985	2175	89.09883
2174	167.4386	2174	143.0648	2174	121.3181	2174	89.16504
2173	167.4446	2173	143.0753	2173	121.3275	2173	89.20392
2172	167.4458	2172	143.0939	2172	121.3168	2172	89.21439
2171	167.4226	2171	143.1167	2171	121.2923	2171	89.21499
2170	167.3985	2170	143.1365	2170	121.2739	2170	89.23024
2169	167.4217	2169	143.1489	2169	121.2869	2169	89.2728
2168	167.5062	2168	143.1584	2168	121.3383	2168	89.33189
2167	167.621	2167	143.1728	2167	121.4058	2167	89.38474
2166	167.7297	2166	143.1933	2166	121.4585	2166	89.41639
2165	167.8138	2165	143.218	2165	121.479	2165	89.4222
2164	167.867	2164	143.251	2164	121.4558	2164	89.39952
2163	167.8924	2163	143.294	2163	121.3718	2163	89.3477
2162	167.8935	2162	143.3289	2162	121.2314	2162	89.27271
2161	167.8542	2161	143.3256	2161	121.0963	2161	89.18307
2160	167.7472	2160	143.2797	2160	121.0473	2160	89.08917
2159	167.5894	2159	143.2273	2159	121.0928	2159	89.01859

2158	167.4555	2158	143.2088	2158	121.1585	2158	89.01023
2157	167.3923	2157	143.2291	2157	121.1757	2157	89.06271
2156	167.3664	2156	143.2631	2156	121.1356	2156	89.12167
2155	167.3308	2155	143.2844	2155	121.0635	2155	89.14553
2154	167.2868	2154	143.2807	2154	120.9917	2154	89.14746
2153	167.2555	2153	143.2595	2153	120.9537	2153	89.15839
2152	167.2389	2152	143.2452	2152	120.9696	2152	89.17884
2151	167.2417	2151	143.26	2151	121.036	2151	89.194
2150	167.2966	2150	143.3021	2150	121.1366	2150	89.21901
2149	167.4209	2149	143.3468	2149	121.2488	2149	89.28639
2148	167.5671	2148	143.3715	2148	121.3408	2148	89.38575
2147	167.6589	2147	143.3761	2147	121.3823	2147	89.45273
2146	167.6675	2146	143.3761	2146	121.3692	2146	89.43315
2145	167.6255	2145	143.3803	2145	121.3261	2145	89.33936
2144	167.5749	2144	143.3788	2144	121.2761	2144	89.23062
2143	167.5302	2143	143.3514	2143	121.219	2143	89.15261
2142	167.4886	2142	143.2893	2142	121.1501	2142	89.11331
2141	167.449	2141	143.2105	2141	121.0849	2141	89.10041
2140	167.4129	2140	143.1493	2140	121.045	2140	89.10028
2139	167.3866	2139	143.1286	2139	121.0339	2139	89.10546
2138	167.3849	2138	143.1453	2138	121.0429	2138	89.11636
2137	167.4189	2137	143.1795	2137	121.0661	2137	89.13701
2136	167.477	2136	143.2109	2136	121.0936	2136	89.16492
2135	167.5285	2135	143.2264	2135	121.103	2135	89.18722
2134	167.5478	2134	143.2204	2134	121.0791	2134	89.19049
2133	167.5311	2133	143.1964	2133	121.0396	2133	89.17326
2132	167.4974	2132	143.1663	2132	121.0223	2132	89.1469
2131	167.4775	2131	143.1432	2131	121.0475	2131	89.12545
2130	167.4929	2130	143.1332	2130	121.1015	2130	89.11607
2129	167.5381	2129	143.1367	2129	121.1522	2129	89.11668
2128	167.5857	2128	143.1515	2128	121.1755	2128	89.12099
2127	167.6147	2127	143.1753	2127	121.1697	2127	89.12657
2126	167.626	2126	143.2042	2126	121.1502	2126	89.13625
2125	167.6278	2125	143.2319	2125	121.1316	2125	89.14999
2124	167.6185	2124	143.2483	2124	121.1133	2124	89.1588
2123	167.5948	2123	143.2424	2123	121.0879	2123	89.15239
2122	167.5665	2122	143.2109	2122	121.0608	2122	89.133
2121	167.5512	2121	143.1667	2121	121.0511	2121	89.11716
2120	167.5549	2120	143.1333	2120	121.065	2120	89.12073
2119	167.5679	2119	143.1241	2119	121.0828	2119	89.14324
2118	167.5787	2118	143.1333	2118	121.0807	2118	89.16921
2117	167.5824	2117	143.1493	2117	121.0592	2117	89.18379
2116	167.5796	2116	143.1688	2116	121.0397	2116	89.18598
2115	167.5723	2115	143.1918	2115	121.0354	2115	89.18514
2114	167.5609	2114	143.2092	2114	121.0388	2114	89.18598

2113	167.5425	2113	143.2071	2113	121.0363	2113	89.18126
2112	167.5133	2112	143.184	2112	121.0257	2112	89.16245
2111	167.4778	2111	143.1527	2111	121.0164	2111	89.13398
2110	167.4505	2110	143.1272	2110	121.019	2110	89.11073
2109	167.4427	2109	143.1147	2109	121.0343	2109	89.10177
2108	167.4505	2108	143.1192	2108	121.0512	2108	89.10261
2107	167.4618	2107	143.1406	2107	121.0546	2107	89.10492
2106	167.4689	2106	143.1692	2106	121.0405	2106	89.1076
2105	167.4722	2105	143.191	2105	121.0198	2105	89.11548
2104	167.4761	2104	143.2032	2104	121.007	2104	89.13344
2103	167.4859	2103	143.2171	2103	121.0083	2103	89.16453
2102	167.501	2102	143.238	2102	121.0234	2102	89.20686
2101	167.5094	2101	143.2537	2101	121.0485	2101	89.24779
2100	167.4984	2100	143.2476	2100	121.0712	2100	89.26837
2099	167.4752	2099	143.2208	2099	121.0707	2099	89.25987
2098	167.4648	2098	143.1917	2098	121.0355	2098	89.23179
2097	167.4811	2097	143.1785	2097	120.9783	2097	89.20086
2096	167.5108	2096	143.1845	2096	120.9268	2096	89.17719
2095	167.5346	2095	143.1967	2095	120.9001	2095	89.16352
2094	167.5481	2094	143.197	2094	120.9017	2094	89.15943
2093	167.5525	2093	143.1761	2093	120.9253	2093	89.15902
2092	167.5411	2092	143.1402	2092	120.9564	2092	89.15194
2091	167.5101	2091	143.103	2091	120.9738	2091	89.1351
2090	167.472	2090	143.0746	2090	120.9639	2090	89.11922
2089	167.4459	2089	143.0604	2089	120.937	2089	89.11666
2088	167.4386	2088	143.0675	2088	120.9208	2088	89.12813
2087	167.4481	2087	143.1043	2087	120.9367	2087	89.14878
2086	167.4742	2086	143.167	2086	120.9839	2086	89.17927
2085	167.5115	2085	143.2335	2085	121.0419	2085	89.21885
2084	167.5423	2084	143.2757	2084	121.0846	2084	89.25287
2083	167.5499	2083	143.2774	2083	121.0954	2083	89.261
2082	167.5373	2082	143.2404	2082	121.0766	2082	89.23805
2081	167.5204	2081	143.1815	2081	121.0471	2081	89.19857
2080	167.509	2080	143.1252	2080	121.0285	2080	89.16114
2079	167.5041	2079	143.0916	2079	121.0315	2079	89.13773
2078	167.5071	2078	143.0848	2078	121.0557	2078	89.13831
2077	167.5169	2077	143.0954	2077	121.0924	2077	89.16979
2076	167.5248	2076	143.1169	2076	121.1256	2076	89.22281
2075	167.5266	2075	143.1532	2075	121.1426	2075	89.27092
2074	167.5307	2074	143.2042	2074	121.146	2074	89.29084
2073	167.5418	2073	143.2516	2073	121.1493	2073	89.28009
2072	167.5467	2072	143.2688	2072	121.1551	2072	89.25231
2071	167.5318	2071	143.2449	2071	121.1477	2071	89.22
2070	167.5061	2070	143.1937	2070	121.1146	2070	89.18722
2069	167.4904	2069	143.1424	2069	121.0646	2069	89.15426

2068	167.4893	2068	143.1135	2068	121.0181	2068	89.12144
2067	167.4929	2067	143.1126	2067	120.9896	2067	89.0908
2066	167.4965	2066	143.1304	2066	120.9889	2066	89.07003
2065	167.5015	2065	143.1541	2065	121.0209	2065	89.07189
2064	167.502	2064	143.1781	2064	121.0649	2064	89.1019
2063	167.4927	2063	143.2022	2063	121.0786	2063	89.14924
2062	167.4845	2062	143.2223	2062	121.0443	2062	89.19435
2061	167.4921	2061	143.2303	2061	120.9955	2061	89.22471
2060	167.5136	2060	143.2268	2060	120.9801	2060	89.24425
2059	167.5368	2059	143.2206	2059	121.0045	2059	89.26584
2058	167.5585	2058	143.2154	2058	121.0351	2058	89.29317
2057	167.5781	2057	143.2057	2057	121.047	2057	89.3139
2056	167.5845	2056	143.1919	2056	121.0463	2056	89.31406
2055	167.5717	2055	143.188	2055	121.0485	2055	89.29607
2054	167.561	2054	143.2066	2054	121.0597	2054	89.27766
2053	167.5816	2053	143.2429	2053	121.0862	2053	89.2753
2052	167.6286	2052	143.2811	2052	121.1353	2052	89.29088
2051	167.6613	2051	143.3073	2051	121.1968	2051	89.30979
2050	167.6429	2050	143.3107	2050	121.2376	2050	89.3059
2049	167.5763	2049	143.2869	2049	121.2348	2049	89.25988
2048	167.5068	2048	143.2525	2048	121.2083	2048	89.19123
2047	167.4914	2047	143.2388	2047	121.1986	2047	89.15788
2046	167.5591	2046	143.2573	2046	121.2168	2046	89.20056
2045	167.6801	2045	143.2829	2045	121.2358	2045	89.29113
2044	167.7766	2044	143.2845	2044	121.223	2044	89.35962
2043	167.7911	2043	143.262	2043	121.1744	2043	89.37485
2042	167.7371	2042	143.2406	2042	121.1252	2042	89.36949
2041	167.6774	2041	143.2392	2041	121.1283	2041	89.38095
2040	167.6646	2040	143.2612	2040	121.2063	2040	89.40085
2039	167.7153	2039	143.3021	2039	121.3286	2039	89.40191
2038	167.8137	2038	143.3418	2038	121.435	2038	89.38124
2037	167.8973	2037	143.332	2037	121.4685	2037	89.34808
2036	167.8799	2036	143.2343	2036	121.4047	2036	89.29665
2035	167.7442	2035	143.0844	2035	121.2729	2035	89.22422
2034	167.5922	2034	142.9891	2034	121.156	2034	89.1666
2033	167.5522	2033	143.0346	2033	121.1409	2033	89.18136
2032	167.641	2032	143.1926	2032	121.2327	2032	89.28961
2031	167.7701	2031	143.3488	2031	121.3428	2031	89.45963
2030	167.8643	2030	143.4133	2030	121.3842	2030	89.63596
2029	167.9055	2029	143.3923	2029	121.3558	2029	89.76068
2028	167.8961	2028	143.3669	2028	121.3214	2028	89.79594
2027	167.8472	2027	143.4036	2027	121.3238	2027	89.74483
2026	167.7946	2026	143.4898	2026	121.3502	2026	89.6381
2025	167.7757	2025	143.5521	2025	121.3592	2025	89.5046
2024	167.7779	2024	143.531	2024	121.314	2024	89.36206

2023	167.7538	2023	143.4327	2023	121.2056	2023	89.23211
2022	167.6905	2022	143.3114	2022	121.0704	2022	89.14559
2021	167.6262	2021	143.2242	2021	120.9768	2021	89.11702
2020	167.6027	2020	143.2024	2020	120.9631	2020	89.12957
2019	167.6339	2019	143.2497	2019	121.007	2019	89.16508
2018	167.7139	2018	143.3546	2018	121.0759	2018	89.22559
2017	167.8101	2017	143.4827	2017	121.1646	2017	89.29607
2016	167.8584	2016	143.5689	2016	121.2511	2016	89.31931
2015	167.8156	2015	143.5517	2015	121.2737	2015	89.25282
2014	167.7033	2014	143.4248	2014	121.1941	2014	89.1244
2013	167.5816	2013	143.2611	2013	121.0497	2013	89.00816
2012	167.4929	2012	143.1685	2012	120.9204	2012	88.96013
2011	167.4467	2011	143.1907	2011	120.852	2011	88.98757
2010	167.4462	2010	143.266	2010	120.8455	2010	89.06783
2009	167.4908	2009	143.2968	2009	120.895	2009	89.1653
2008	167.5537	2008	143.2527	2008	120.9832	2008	89.24151
2007	167.5989	2007	143.1847	2007	121.0631	2007	89.27833
2006	167.6187	2006	143.143	2006	121.0885	2006	89.28514
2005	167.6327	2005	143.1275	2005	121.0599	2005	89.27622
2004	167.6573	2004	143.1207	2004	121.0122	2004	89.25371
2003	167.6924	2003	143.127	2003	120.9673	2003	89.21704
2002	167.7235	2002	143.1612	2002	120.9285	2002	89.17303
2001	167.7253	2001	143.2198	2001	120.9072	2001	89.13225
2000	167.6845	2000	143.2861	2000	120.9261	2000	89.10753
1999	167.6272	1999	143.349	1999	120.9948	1999	89.11647
1998	167.5991	1998	143.4038	1998	121.0946	1998	89.16702
1997	167.6061	1997	143.4419	1997	121.1807	1997	89.23288
1996	167.6055	1996	143.4589	1996	121.2053	1996	89.27086
1995	167.5712	1995	143.4624	1995	121.1533	1995	89.27152
1994	167.5369	1994	143.4589	1994	121.0591	1994	89.26501
1993	167.5489	1993	143.4396	1993	120.986	1993	89.26917
1992	167.5918	1992	143.3829	1992	120.9566	1992	89.25209
1991	167.6081	1991	143.2724	1991	120.9176	1991	89.17572
1990	167.5848	1990	143.1208	1990	120.8333	1990	89.06104
1989	167.5555	1989	142.9708	1989	120.7545	1989	88.96983
1988	167.5346	1988	142.8702	1988	120.7455	1988	88.93827
1987	167.5049	1987	142.8403	1987	120.7933	1987	88.95524
1986	167.4651	1986	142.8686	1986	120.8396	1986	88.99287
1985	167.4497	1985	142.9333	1985	120.8715	1985	89.03555
1984	167.4893	1984	143.0244	1984	120.9273	1984	89.08431
1983	167.576	1983	143.1347	1983	121.018	1983	89.1486
1982	167.6709	1982	143.2402	1982	121.1024	1982	89.22819
1981	167.7296	1981	143.3096	1981	121.1463	1981	89.29966
1980	167.7311	1980	143.3352	1980	121.1628	1980	89.33636
1979	167.696	1979	143.3317	1979	121.167	1979	89.34389

1978	167.6662	1978	143.3029	1978	121.1371	1978	89.34887
1977	167.6534	1977	143.2433	1977	121.0633	1977	89.34525
1976	167.6285	1976	143.1736	1976	120.9877	1976	89.29609
1975	167.5794	1975	143.1321	1975	120.9416	1975	89.20119
1974	167.5392	1974	143.1324	1974	120.9012	1974	89.11286
1973	167.5379	1973	143.159	1973	120.8433	1973	89.07258
1972	167.5627	1972	143.1991	1972	120.7943	1972	89.07286
1971	167.59	1971	143.2462	1971	120.7872	1971	89.09556
1970	167.6168	1970	143.2761	1970	120.8105	1970	89.14415
1969	167.6373	1969	143.2545	1969	120.8307	1969	89.21272
1968	167.6259	1968	143.1859	1968	120.839	1968	89.26331
1967	167.576	1967	143.1213	1967	120.8447	1967	89.26711
1966	167.524	1966	143.0993	1966	120.846	1966	89.23847
1965	167.4971	1965	143.1084	1965	120.8384	1965	89.19869
1964	167.4769	1964	143.1216	1964	120.8352	1964	89.14764
1963	167.4457	1963	143.139	1963	120.8513	1963	89.09626
1962	167.4311	1962	143.1837	1962	120.8814	1962	89.08611
1961	167.4699	1961	143.2762	1961	120.9157	1961	89.143
1960	167.5469	1960	143.4092	1960	120.9561	1960	89.23103
1959	167.6066	1959	143.5358	1959	120.9977	1959	89.28958
1958	167.6147	1958	143.5849	1958	121.0133	1958	89.29679
1957	167.5794	1957	143.5176	1957	120.9811	1957	89.27108
1956	167.5301	1956	143.376	1956	120.915	1956	89.23624
1955	167.4967	1955	143.2523	1955	120.8472	1955	89.20585
1954	167.4992	1954	143.2071	1954	120.7937	1954	89.18608
1953	167.5357	1953	143.2297	1953	120.7532	1953	89.17149
1952	167.5841	1952	143.2703	1952	120.7267	1952	89.14773
1951	167.6254	1951	143.2934	1951	120.7217	1951	89.11268
1950	167.6538	1950	143.2919	1950	120.7409	1950	89.08181
1949	167.6636	1949	143.2752	1949	120.7754	1949	89.06537
1948	167.65	1948	143.2602	1948	120.8119	1948	89.05924
1947	167.6307	1947	143.2628	1947	120.8444	1947	89.06948
1946	167.6412	1946	143.2852	1946	120.8773	1946	89.11948
1945	167.6915	1945	143.3146	1945	120.9143	1945	89.21137
1944	167.7477	1944	143.3378	1944	120.9481	1944	89.30163
1943	167.7703	1943	143.3533	1943	120.9658	1943	89.34373
1942	167.7583	1942	143.3627	1942	120.964	1942	89.34563
1941	167.7318	1941	143.3638	1941	120.9466	1941	89.34655
1940	167.7012	1940	143.3608	1940	120.9218	1940	89.35642
1939	167.6747	1939	143.3643	1939	120.9025	1939	89.35961
1938	167.6702	1938	143.3771	1938	120.9009	1938	89.35232
1937	167.6962	1937	143.3962	1937	120.9304	1937	89.35187
1936	167.7343	1936	143.422	1936	120.9946	1936	89.37468
1935	167.7582	1935	143.4506	1935	121.0716	1935	89.4171
1934	167.7629	1934	143.4633	1934	121.1248	1934	89.46184

1933	167.7588	1933	143.4426	1933	121.1364	1933	89.49136
1932	167.7505	1932	143.3989	1932	121.1183	1932	89.4971
1931	167.7378	1931	143.3658	1931	121.0931	1931	89.48536
1930	167.7285	1930	143.3629	1930	121.0791	1930	89.47105
1929	167.7297	1929	143.3793	1929	121.0847	1929	89.46157
1928	167.7362	1928	143.3951	1928	121.102	1928	89.45076
1927	167.7409	1927	143.406	1927	121.1113	1927	89.43596
1926	167.7461	1926	143.4128	1926	121.102	1926	89.42791
1925	167.7537	1925	143.4024	1925	121.0798	1925	89.43493
1924	167.7575	1924	143.366	1924	121.0579	1924	89.45067
1923	167.756	1923	143.3282	1923	121.043	1923	89.46452
1922	167.7542	1922	143.3233	1922	121.0267	1922	89.47168
1921	167.744	1921	143.3452	1921	120.9988	1921	89.46405
1920	167.7068	1920	143.3547	1920	120.9658	1920	89.42988
1919	167.6456	1919	143.3252	1919	120.9419	1919	89.37289
1918	167.6022	1918	143.2746	1918	120.9389	1918	89.32808
1917	167.6127	1917	143.2448	1917	120.96	1917	89.32962
1916	167.6568	1916	143.2514	1916	120.9916	1916	89.36437
1915	167.6938	1915	143.2792	1915	121.0219	1915	89.39776
1914	167.708	1914	143.3096	1914	121.0456	1914	89.41563
1913	167.7031	1913	143.3317	1913	121.0565	1913	89.41857
1912	167.6845	1912	143.3415	1912	121.0457	1912	89.40493
1911	167.6616	1911	143.3394	1911	121.0112	1911	89.37392
1910	167.6544	1910	143.3317	1910	120.9728	1910	89.34053
1909	167.6724	1909	143.3292	1909	120.9618	1909	89.32653
1908	167.6968	1908	143.3364	1908	120.9792	1908	89.33359
1907	167.7105	1907	143.35	1907	121.0017	1907	89.35114
1906	167.7224	1906	143.3638	1906	121.0203	1906	89.37783
1905	167.7427	1905	143.3679	1905	121.0403	1905	89.41043
1904	167.7578	1904	143.3585	1904	121.0595	1904	89.43134
1903	167.7511	1903	143.3452	1903	121.068	1903	89.42609
1902	167.7309	1902	143.3407	1902	121.0664	1902	89.40541
1901	167.7167	1901	143.3452	1901	121.0653	1901	89.39286
1900	167.7134	1900	143.3496	1900	121.0663	1900	89.39474
1899	167.7156	1899	143.3502	1899	121.0605	1899	89.39851
1898	167.7266	1898	143.3519	1898	121.0471	1898	89.3952
1897	167.7501	1897	143.358	1897	121.0405	1897	89.38729
1896	167.7733	1896	143.3659	1896	121.0488	1896	89.37953
1895	167.7794	1895	143.3741	1895	121.0581	1895	89.37631
1894	167.7726	1894	143.3868	1894	121.0535	1894	89.38603
1893	167.7681	1893	143.4065	1893	121.0461	1893	89.41271
1892	167.7677	1892	143.4285	1892	121.0586	1892	89.44592
1891	167.763	1891	143.4424	1891	121.0917	1891	89.46555
1890	167.7569	1890	143.4436	1890	121.1236	1890	89.46132
1889	167.76	1889	143.4357	1889	121.1358	1889	89.44349

1888	167.7666	1888	143.422	1888	121.1205	1888	89.42483
1887	167.7647	1887	143.4069	1887	121.0907	1887	89.41009
1886	167.7549	1886	143.3934	1886	121.0676	1886	89.40118
1885	167.7428	1885	143.3785	1885	121.0598	1885	89.39518
1884	167.7309	1884	143.3611	1884	121.0621	1884	89.38654
1883	167.7243	1883	143.3472	1883	121.0652	1883	89.3784
1882	167.7323	1882	143.3447	1882	121.0665	1882	89.3828
1881	167.7513	1881	143.3536	1881	121.0676	1881	89.40148
1880	167.7599	1880	143.3652	1880	121.0669	1880	89.41588
1879	167.747	1879	143.3698	1879	121.0612	1879	89.40901
1878	167.7318	1878	143.3657	1878	121.0525	1878	89.38951
1877	167.7385	1877	143.3583	1877	121.0474	1877	89.37939
1876	167.7622	1876	143.3537	1876	121.0477	1876	89.38533
1875	167.7796	1875	143.3564	1875	121.0471	1875	89.39702
1874	167.7849	1874	143.3706	1874	121.0401	1874	89.40739
1873	167.7904	1873	143.3972	1873	121.0283	1873	89.41827
1872	167.8013	1872	143.4296	1872	121.012	1872	89.42928
1871	167.8091	1871	143.4519	1871	120.9816	1871	89.43417
1870	167.8102	1870	143.4469	1870	120.9309	1870	89.42908
1869	167.8171	1869	143.4218	1869	120.8993	1869	89.42555
1868	167.8315	1868	143.4025	1868	120.9297	1868	89.43101
1867	167.8342	1867	143.3938	1867	120.9765	1867	89.42838
1866	167.8233	1866	143.3887	1866	120.9967	1866	89.41202
1865	167.8151	1865	143.3859	1865	121.0056	1865	89.39865
1864	167.8149	1864	143.3852	1864	121.023	1864	89.39855
1863	167.8168	1863	143.3861	1863	121.0444	1863	89.40806
1862	167.8169	1862	143.3861	1862	121.0553	1862	89.41896
1861	167.8161	1861	143.3824	1861	121.0542	1861	89.42508
1860	167.8132	1860	143.3749	1860	121.0516	1860	89.42103
1859	167.8078	1859	143.3676	1859	121.0564	1859	89.40765
1858	167.8078	1858	143.3663	1858	121.0735	1858	89.39756
1857	167.818	1857	143.3712	1857	121.0993	1857	89.40145
1856	167.8286	1856	143.376	1856	121.1179	1856	89.41331
1855	167.8286	1855	143.3773	1855	121.1162	1855	89.4209
1854	167.8209	1854	143.3769	1854	121.0957	1854	89.42142
1853	167.8159	1853	143.3797	1853	121.0711	1853	89.42128
1852	167.8164	1852	143.3895	1852	121.0574	1852	89.42296
1851	167.8201	1851	143.4023	1851	121.0563	1851	89.42301
1850	167.829	1850	143.4135	1850	121.0604	1850	89.42287
1849	167.8447	1849	143.4269	1849	121.0558	1849	89.42633
1848	167.8571	1848	143.4473	1848	121.026	1848	89.42858
1847	167.8516	1847	143.4625	1847	120.9591	1847	89.41872
1846	167.827	1846	143.4447	1846	120.8682	1846	89.39549
1845	167.8005	1845	143.3825	1845	120.8043	1845	89.37344
1844	167.7883	1844	143.3122	1844	120.8182	1844	89.36854

1843	167.793	1843	143.2866	1843	120.8858	1843	89.38191
1842	167.8122	1842	143.3121	1842	120.9477	1842	89.40198
1841	167.8394	1841	143.3528	1841	120.981	1841	89.41775
1840	167.8619	1840	143.3782	1840	120.9948	1840	89.42274
1839	167.8709	1839	143.384	1839	121.0026	1839	89.41485
1838	167.87	1838	143.3802	1838	121.0112	1838	89.40088
1837	167.8696	1837	143.3751	1837	121.0247	1837	89.39414
1836	167.8721	1836	143.3755	1836	121.0436	1836	89.40119
1835	167.8749	1835	143.3916	1835	121.061	1835	89.41755
1834	167.8806	1834	143.4315	1834	121.0719	1834	89.4386
1833	167.8927	1833	143.4917	1833	121.0821	1833	89.46409
1832	167.9069	1832	143.5513	1832	121.1013	1832	89.48969
1831	167.9104	1831	143.5756	1831	121.1199	1831	89.49906
1830	167.9042	1830	143.5586	1830	121.1284	1830	89.48941
1829	167.9056	1829	143.5373	1829	121.1389	1829	89.4819
1828	167.9159	1828	143.5361	1828	121.1503	1828	89.48124
1827	167.9245	1827	143.5418	1827	121.1641	1827	89.476
1826	167.9289	1826	143.5267	1826	121.174	1826	89.46342
1825	167.9318	1825	143.4818	1825	121.1613	1825	89.44734
1824	167.9325	1824	143.4332	1824	121.1229	1824	89.43128
1823	167.928	1823	143.4069	1823	121.0774	1823	89.41403
1822	167.925	1822	143.4043	1822	121.0508	1822	89.40089
1821	167.9327	1821	143.4147	1821	121.0567	1821	89.40204
1820	167.9483	1820	143.4279	1820	121.0859	1820	89.41493
1819	167.9598	1819	143.4372	1819	121.1118	1819	89.4247
1818	167.9627	1818	143.4395	1818	121.1159	1818	89.42289
1817	167.9628	1817	143.437	1817	121.1033	1817	89.41598
1816	167.9621	1816	143.4374	1816	121.0864	1816	89.41235
1815	167.9601	1815	143.4476	1815	121.0687	1815	89.41184
1814	167.9617	1814	143.4678	1814	121.0532	1814	89.41258
1813	167.9724	1813	143.4914	1813	121.0509	1813	89.41551
1812	167.9869	1812	143.507	1812	121.0703	1812	89.42064
1811	167.9928	1811	143.505	1811	121.0945	1811	89.42581
1810	167.9899	1810	143.4847	1810	121.0954	1810	89.42999
1809	167.988	1809	143.4555	1809	121.0763	1809	89.43253
1808	167.9858	1808	143.4292	1808	121.0577	1808	89.42799
1807	167.9771	1807	143.4155	1807	121.0456	1807	89.41465
1806	167.9697	1806	143.4215	1806	121.0426	1806	89.40491
1805	167.9762	1805	143.4472	1805	121.0581	1805	89.41256
1804	167.9933	1804	143.4836	1804	121.0914	1804	89.43368
1803	168.0054	1803	143.5139	1803	121.1181	1803	89.44648
1802	168.006	1802	143.5247	1802	121.1129	1802	89.43615
1801	168.0064	1801	143.5179	1801	121.088	1801	89.41289
1800	168.009	1800	143.4971	1800	121.0586	1800	89.38939
1799	168.0032	1799	143.4629	1799	121.0064	1799	89.36474

1798	167.9924	1798	143.4305	1798	120.9316	1798	89.34271
1797	167.9882	1797	143.4242	1797	120.8658	1797	89.33327
1796	167.9855	1796	143.4514	1796	120.8262	1796	89.3339
1795	167.9704	1795	143.491	1795	120.7996	1795	89.33264
1794	167.9423	1794	143.5054	1794	120.7707	1794	89.32443
1793	167.9226	1793	143.4721	1793	120.7595	1793	89.31606
1792	167.9297	1792	143.4171	1792	120.8108	1792	89.31658
1791	167.9514	1791	143.385	1791	120.8951	1791	89.32154
1790	167.972	1790	143.3852	1790	120.9385	1790	89.3201
1789	167.9916	1789	143.4038	1789	120.9435	1789	89.31453
1788	168.0073	1788	143.4243	1788	120.9465	1788	89.30974
1787	168.0127	1787	143.4321	1787	120.9588	1787	89.30342
1786	168.0122	1786	143.423	1786	120.969	1786	89.29587
1785	168.0175	1785	143.4097	1785	120.9739	1785	89.29384
1784	168.0312	1784	143.4182	1784	120.9887	1784	89.30185
1783	168.0454	1783	143.4529	1783	121.0239	1783	89.31162
1782	168.0539	1782	143.4843	1782	121.0615	1782	89.3123
1781	168.0569	1781	143.4864	1781	121.0728	1781	89.30168
1780	168.0541	1780	143.4656	1780	121.0491	1780	89.2826
1779	168.0444	1779	143.4508	1779	121.0065	1779	89.26191
1778	168.0366	1778	143.461	1778	120.9734	1778	89.24937
1777	168.0409	1777	143.4976	1777	120.963	1777	89.25059
1776	168.0505	1776	143.5484	1776	120.9583	1776	89.25242
1775	168.0495	1775	143.5905	1775	120.9302	1775	89.23462
1774	168.029	1774	143.5917	1774	120.8631	1774	89.19127
1773	168.0029	1773	143.5366	1773	120.7834	1773	89.1423
1772	167.9963	1772	143.4759	1772	120.7831	1772	89.12695
1771	168.0046	1771	143.4587	1771	120.8593	1771	89.14513
1770	168.0103	1770	143.4552	1770	120.913	1770	89.15661
1769	168.0131	1769	143.427	1769	120.9276	1769	89.14804
1768	168.0123	1768	143.385	1768	120.9344	1768	89.12955
1767	168.0092	1767	143.3702	1767	120.9436	1767	89.11023
1766	168.0152	1766	143.4053	1766	120.9487	1766	89.09819
1765	168.0338	1765	143.4657	1765	120.9485	1765	89.09281
1764	168.0486	1764	143.5086	1764	120.9434	1764	89.0843
1763	168.0394	1763	143.504	1763	120.9328	1763	89.05877
1762	168.0147	1762	143.4612	1762	120.9176	1762	89.02029
1761	168.0053	1761	143.4243	1761	120.9045	1761	88.99029
1760	168.0146	1760	143.4224	1760	120.8976	1760	88.96946
1759	168.0178	1759	143.4399	1759	120.8999	1759	88.93895
1758	168.0006	1758	143.4372	1758	120.905	1758	88.88888
1757	167.9691	1757	143.4033	1757	120.9023	1757	88.82527
1756	167.935	1756	143.3758	1756	120.887	1756	88.75823
1755	167.9029	1755	143.3882	1755	120.8681	1755	88.69016
1754	167.8713	1754	143.425	1754	120.858	1754	88.6172

1753	167.8287	1753	143.4483	1753	120.8495	1753	88.52724
1752	167.7465	1752	143.4387	1752	120.8198	1752	88.38726
1751	167.5946	1751	143.4063	1751	120.7515	1751	88.16094
1750	167.3748	1750	143.3668	1750	120.6529	1750	87.84885
1749	167.1096	1749	143.317	1749	120.5553	1749	87.47546
1748	166.8066	1748	143.2628	1748	120.5021	1748	87.06161
1747	166.4692	1747	143.2224	1747	120.5028	1747	86.62099
1746	166.1012	1746	143.2018	1746	120.5409	1746	86.14446
1745	165.6859	1745	143.1924	1745	120.6066	1745	85.58811
1744	165.1985	1744	143.1865	1744	120.6862	1744	84.92133
1743	164.6476	1743	143.1828	1743	120.7425	1743	84.17854
1742	164.058	1742	143.1619	1742	120.7268	1742	83.40009
1741	163.4167	1741	143.0875	1741	120.6363	1741	82.55272
1740	162.708	1740	142.9662	1740	120.534	1740	81.61761
1739	162.0139	1739	142.8763	1739	120.4858	1739	80.7268
1738	161.441	1738	142.8644	1738	120.479	1738	80.01767
1737	160.9738	1737	142.8798	1737	120.466	1737	79.44347
1736	160.5145	1736	142.8406	1736	120.422	1736	78.87437
1735	159.9777	1735	142.7057	1735	120.3393	1735	78.20674
1734	159.4259	1734	142.5532	1734	120.2783	1734	77.5126
1733	158.9996	1733	142.516	1733	120.3249	1733	76.97615
1732	158.645	1732	142.5837	1732	120.4128	1732	76.53882
1731	158.2265	1731	142.663	1731	120.4837	1731	76.04003
1730	157.6812	1730	142.6969	1730	120.513	1730	75.42817
1729	156.9899	1729	142.672	1729	120.4649	1729	74.72926
1728	156.1762	1728	142.6266	1728	120.3518	1728	74.04122
1727	155.2776	1727	142.5972	1727	120.233	1727	73.47455
1726	154.3499	1726	142.5908	1726	120.1361	1726	73.15151
1725	153.4846	1725	142.5843	1725	120.0384	1725	73.225
1724	152.7972	1724	142.566	1724	119.9189	1724	73.82933
1723	152.3602	1723	142.5658	1723	119.8047	1723	74.91827
1722	152.1547	1722	142.62	1722	119.7389	1722	76.22676
1721	152.1438	1721	142.7175	1721	119.7481	1721	77.52829
1720	152.4115	1720	142.8066	1720	119.824	1720	78.84085
1719	153.2205	1719	142.8045	1719	119.8505	1719	80.37268
1718	154.8345	1718	142.6602	1718	119.6327	1718	82.20529
1717	156.8352	1717	142.4935	1717	119.3507	1717	83.8894
1716	158.4168	1716	142.4321	1716	119.3239	1716	84.96529
1715	159.4705	1715	142.4458	1715	119.4822	1715	85.57826
1714	160.2418	1714	142.4747	1714	119.6661	1714	85.98439
1713	160.8193	1713	142.4869	1713	119.8059	1713	86.25939
1712	161.2642	1712	142.4824	1712	119.9056	1712	86.45426
1711	161.6472	1711	142.4783	1711	119.9925	1711	86.61926
1710	162.0081	1710	142.4858	1710	120.0712	1710	86.77471
1709	162.3233	1709	142.51	1709	120.1354	1709	86.90393

1708	162.5818	1708	142.5338	1708	120.1852	1708	87.0027
1707	162.8336	1707	142.5201	1707	120.2165	1707	87.09046
1706	163.1334	1706	142.4618	1706	120.2296	1706	87.18317
1705	163.4288	1705	142.4074	1705	120.2436	1705	87.267
1704	163.6077	1704	142.387	1704	120.2476	1704	87.31347
1703	163.6601	1703	142.3642	1703	120.212	1703	87.31305
1702	163.6601	1702	142.2856	1702	120.1409	1702	87.28533
1701	163.7419	1701	142.0991	1701	120.0571	1701	87.2545
1700	163.9556	1700	141.8269	1700	119.9503	1700	87.23035
1699	164.1704	1699	141.6208	1699	119.8728	1699	87.23242
1698	164.3057	1698	141.5053	1698	119.9001	1698	87.23717
1697	164.4138	1697	141.3618	1697	120.0529	1697	87.21979
1696	164.5494	1696	141.1561	1696	120.2959	1696	87.21343
1695	164.6694	1695	140.9499	1695	120.42	1695	87.22076
1694	164.732	1694	140.8273	1694	120.4137	1694	87.21972
1693	164.7587	1693	140.7618	1693	120.4114	1693	87.19882
1692	164.7773	1692	140.6685	1692	120.4463	1692	87.16563
1691	164.8104	1691	140.5236	1691	120.4889	1691	87.13469
1690	164.8607	1690	140.351	1690	120.4938	1690	87.1018
1689	164.9149	1689	140.1936	1689	120.4707	1689	87.0628
1688	164.9565	1688	140.0655	1688	120.4933	1688	87.02924
1687	164.9909	1687	139.9562	1687	120.6108	1687	87.01936
1686	165.0291	1686	139.8447	1686	120.8293	1686	87.04106
1685	165.1164	1685	139.6892	1685	121.0439	1685	87.056
1684	165.2653	1684	139.4895	1684	121.0859	1684	87.03171
1683	165.3723	1683	139.2758	1683	120.8918	1683	86.98844
1682	165.4141	1682	139.0629	1682	120.6669	1682	86.93183
1681	165.4489	1681	138.8585	1681	120.5731	1681	86.86926
1680	165.491	1680	138.6725	1680	120.5763	1680	86.82172
1679	165.5197	1679	138.5038	1679	120.5769	1679	86.78102
1678	165.5215	1678	138.3348	1678	120.5359	1678	86.73504
1677	165.5027	1677	138.1281	1677	120.4676	1677	86.66981
1676	165.5042	1676	137.8945	1676	120.395	1676	86.60043
1675	165.5597	1675	137.699	1675	120.3492	1675	86.55694
1674	165.6431	1674	137.5767	1674	120.3554	1674	86.54665
1673	165.7047	1673	137.476	1673	120.3958	1673	86.53268
1672	165.7332	1672	137.3328	1672	120.4123	1672	86.48799
1671	165.7541	1671	137.1481	1671	120.3837	1671	86.4241
1670	165.806	1670	136.9937	1670	120.3861	1670	86.37681
1669	165.8799	1669	136.8842	1669	120.4899	1669	86.35999
1668	165.9309	1668	136.7448	1668	120.5848	1668	86.34055
1667	165.9526	1667	136.5641	1667	120.6083	1667	86.30232
1666	165.9552	1666	136.3693	1666	120.5902	1666	86.25132
1665	165.9415	1665	136.1602	1665	120.5542	1665	86.18813
1664	165.9238	1664	135.949	1664	120.4998	1664	86.11508

1663	165.9252	1663	135.7851	1663	120.4388	1663	86.04731
1662	165.9504	1662	135.6847	1662	120.4182	1662	86.00456
1661	165.9693	1661	135.573	1661	120.4342	1661	85.97037
1660	165.9702	1660	135.4115	1660	120.4401	1660	85.92458
1659	165.9615	1659	135.2246	1659	120.4301	1659	85.87347
1658	165.9619	1658	135.0779	1658	120.4305	1658	85.84215
1657	165.9669	1657	134.9431	1657	120.488	1657	85.82421
1656	165.983	1656	134.7938	1656	120.6197	1656	85.81504
1655	165.9995	1655	134.5824	1655	120.8307	1655	85.80636
1654	166.033	1654	134.53	1654	120.9693	1654	85.80126
1653	166.0707	1653	134.7779	1653	120.8953	1653	85.8114
1652	166.0549	1652	134.8648	1652	120.5943	1652	85.81607
1651	166.0398	1651	134.7622	1651	120.4088	1651	85.81516
1650	166.0436	1650	134.6438	1650	120.355	1650	85.80569
1649	166.0457	1649	134.4783	1649	120.3515	1649	85.78748
1648	166.0287	1648	134.3479	1648	120.2786	1648	85.75036
1647	166.0353	1647	134.4385	1647	120.2071	1647	85.76165
1646	166.0769	1646	134.6409	1646	120.2548	1646	85.84006
1645	166.0954	1645	134.6708	1645	120.2595	1645	85.89378
1644	166.094	1644	134.5757	1644	120.2274	1644	85.90592
1643	166.099	1643	134.479	1643	120.2002	1643	85.9161
1642	166.1074	1642	134.4131	1642	120.1781	1642	85.93295
1641	166.122	1641	134.4068	1641	120.1702	1641	85.96933
1640	166.1404	1640	134.4343	1640	120.1877	1640	86.01764
1639	166.1621	1639	134.4545	1639	120.2217	1639	86.06486
1638	166.1773	1638	134.453	1638	120.2429	1638	86.09991
1637	166.1882	1637	134.5507	1637	120.2473	1637	86.14591
1636	166.2285	1636	134.9205	1636	120.3225	1636	86.2672
1635	166.284	1635	135.3355	1635	120.4338	1635	86.42866
1634	166.3101	1634	135.4871	1634	120.4251	1634	86.52246
1633	166.3227	1633	135.4739	1633	120.3899	1633	86.55465
1632	166.3362	1632	135.4338	1632	120.381	1632	86.57399
1631	166.3489	1631	135.4214	1631	120.398	1631	86.60464
1630	166.3686	1630	135.4883	1630	120.4384	1630	86.66406
1629	166.3925	1629	135.6487	1629	120.4881	1629	86.74415
1628	166.4157	1628	135.8713	1628	120.5189	1628	86.82949
1627	166.4311	1627	136.0696	1627	120.5184	1627	86.90112
1626	166.4344	1626	136.1937	1626	120.491	1626	86.95088
1625	166.4259	1625	136.2777	1625	120.4479	1625	86.98689
1624	166.4223	1624	136.4179	1624	120.4037	1624	87.03455
1623	166.4502	1623	136.6671	1623	120.404	1623	87.11974
1622	166.501	1622	136.9227	1622	120.4553	1622	87.22246
1621	166.5417	1621	137.0725	1621	120.5078	1621	87.29954
1620	166.5658	1620	137.1247	1620	120.5526	1620	87.34468
1619	166.5808	1619	137.136	1619	120.5924	1619	87.37589

1618	166.5899	1618	137.1978	1618	120.6111	1618	87.41077
1617	166.6043	1617	137.4205	1617	120.623	1617	87.47491
1616	166.6301	1616	137.7477	1616	120.6703	1616	87.57463
1615	166.654	1615	138.0016	1615	120.7121	1615	87.66759
1614	166.668	1614	138.1386	1614	120.7319	1614	87.72305
1613	166.6738	1613	138.2125	1613	120.7446	1613	87.75239
1612	166.6737	1612	138.2786	1612	120.7526	1612	87.77599
1611	166.6753	1611	138.3758	1611	120.7474	1611	87.80557
1610	166.6805	1610	138.5014	1610	120.7334	1610	87.83815
1609	166.6837	1609	138.6349	1609	120.7245	1609	87.86861
1608	166.6831	1608	138.7655	1608	120.7297	1608	87.89871
1607	166.6861	1607	138.888	1607	120.7507	1607	87.93004
1606	166.6918	1606	138.9889	1606	120.7779	1606	87.95546
1605	166.6905	1605	139.0665	1605	120.7942	1605	87.96871
1604	166.6796	1604	139.1365	1604	120.7906	1604	87.97235
1603	166.6679	1603	139.214	1603	120.7836	1603	87.97636
1602	166.6629	1602	139.2981	1602	120.8018	1602	87.9878
1601	166.6613	1601	139.3767	1601	120.8435	1601	88.00428
1600	166.6603	1600	139.4449	1600	120.882	1600	88.02217
1599	166.6641	1599	139.5042	1599	120.902	1599	88.04091
1598	166.6724	1598	139.5549	1598	120.903	1598	88.05715
1597	166.6788	1597	139.5999	1597	120.8883	1597	88.06654
1596	166.6818	1596	139.6491	1596	120.8655	1596	88.07176
1595	166.6899	1595	139.7091	1595	120.8526	1595	88.08269
1594	166.7083	1594	139.7722	1594	120.8635	1594	88.10409
1593	166.7288	1593	139.8244	1593	120.8846	1593	88.12871
1592	166.7443	1592	139.8638	1592	120.8956	1592	88.14901
1591	166.7592	1591	139.8988	1591	120.8998	1591	88.16541
1590	166.7787	1590	139.9324	1590	120.9168	1590	88.17982
1589	166.7969	1589	139.9592	1589	120.9473	1589	88.1899
1588	166.8071	1588	139.9745	1588	120.9732	1588	88.19301
1587	166.8114	1587	139.9796	1587	120.9869	1587	88.19046
1586	166.8126	1586	139.973	1586	120.9939	1586	88.1814
1585	166.8065	1585	139.9542	1585	120.9939	1585	88.16361
1584	166.791	1584	139.9278	1584	120.9836	1584	88.13756
1583	166.7721	1583	139.8947	1583	120.9667	1583	88.10673
1582	166.7551	1582	139.8578	1582	120.9574	1582	88.07731
1581	166.7421	1581	139.8311	1581	120.9601	1581	88.05554
1580	166.7337	1580	139.8152	1580	120.9694	1580	88.03881
1579	166.7292	1579	139.7817	1579	120.9736	1579	88.01521
1578	166.7155	1578	139.6911	1578	120.9488	1578	87.96651
1577	166.6832	1577	139.5564	1577	120.878	1577	87.88836
1576	166.6602	1576	139.467	1576	120.8379	1576	87.83433
1575	166.6654	1575	139.4514	1575	120.8457	1575	87.8384
1574	166.6817	1574	139.456	1574	120.8443	1574	87.85651

1573	166.6971	1573	139.4457	1573	120.8471	1573	87.85988
1572	166.7104	1572	139.4123	1572	120.872	1572	87.85843
1571	166.7245	1571	139.3608	1571	120.9035	1571	87.85939
1570	166.7514	1570	139.3058	1570	120.9189	1570	87.86061
1569	166.7873	1569	139.2486	1569	120.9267	1569	87.86134
1568	166.8166	1568	139.1738	1568	120.9087	1568	87.85739
1567	166.8381	1567	139.0786	1567	120.8791	1567	87.84229
1566	166.8586	1566	138.9768	1566	120.8632	1566	87.82049
1565	166.8746	1565	138.8772	1565	120.8585	1565	87.79825
1564	166.887	1564	138.8	1564	120.8599	1564	87.77774
1563	166.908	1563	138.7449	1563	120.898	1563	87.7766
1562	166.941	1562	138.7005	1562	121.0463	1562	87.79726
1561	166.9979	1561	138.6948	1561	121.3744	1561	87.88538
1560	167.0448	1560	138.6771	1560	121.7449	1560	87.95291
1559	166.9831	1559	138.6091	1559	121.53	1559	87.79914
1558	166.8819	1558	138.4958	1558	121.0808	1558	87.62275
1557	166.8022	1557	138.2445	1557	120.6562	1557	87.51132
1556	166.7528	1556	137.9829	1556	120.4421	1556	87.40721
1555	166.7564	1555	137.8084	1555	120.4255	1555	87.36104
1554	166.7692	1554	137.6738	1554	120.5093	1554	87.33694
1553	166.8006	1553	137.5981	1553	120.6324	1553	87.34897
1552	166.8305	1552	137.5311	1552	120.7739	1552	87.36137
1551	166.8525	1551	137.4644	1551	120.8959	1551	87.36098
1550	166.8455	1550	137.3868	1550	120.9454	1550	87.32549
1549	166.8105	1549	137.2981	1549	120.8968	1549	87.26273
1548	166.7639	1548	137.1837	1548	120.8008	1548	87.19676
1547	166.725	1547	137.0528	1547	120.7223	1547	87.14912
1546	166.6855	1546	136.9121	1546	120.6419	1546	87.10581
1545	166.6265	1545	136.7895	1545	120.4617	1545	87.03567
1544	166.5371	1544	136.6922	1544	120.1704	1544	86.92473
1543	166.4494	1543	136.6145	1543	119.883	1543	86.81615
1542	166.3815	1542	136.5103	1542	119.6817	1542	86.72446
1541	166.4068	1541	136.545	1541	119.7649	1541	86.72135
1540	166.5931	1540	136.9067	1540	120.4738	1540	86.93038
1539	166.7383	1539	137.1559	1539	121.0175	1539	87.14365
1538	166.7434	1538	137.0911	1538	120.9446	1538	87.1841
1537	166.7232	1537	136.9759	1537	120.7496	1537	87.16226
1536	166.7236	1536	136.8887	1536	120.6524	1536	87.15561
1535	166.7474	1535	136.8469	1535	120.6664	1535	87.18537
1534	166.8026	1534	136.9268	1534	120.7477	1534	87.26344
1533	166.877	1533	137.1154	1533	120.8644	1533	87.36646
1532	166.9346	1532	137.2382	1532	120.9369	1532	87.44395
1531	166.9625	1531	137.2321	1531	120.9416	1531	87.4763
1530	166.9827	1530	137.1932	1530	120.9323	1530	87.49778
1529	167.0019	1529	137.1818	1529	120.9238	1529	87.52434

1528	167.0122	1528	137.2076	1528	120.8763	1528	87.54697
1527	167.0054	1527	137.2577	1527	120.7545	1527	87.5539
1526	166.9914	1526	137.3274	1526	120.5905	1526	87.55276
1525	166.9875	1525	137.3984	1525	120.4713	1525	87.55646
1524	166.9983	1524	137.44	1524	120.431	1524	87.56812
1523	167.0093	1523	137.4506	1523	120.4279	1523	87.5828
1522	167.0274	1522	137.5589	1522	120.4699	1522	87.62841
1521	167.0737	1521	137.8314	1521	120.6641	1521	87.73992
1520	167.121	1520	138.0707	1520	120.876	1520	87.85992
1519	167.1542	1519	138.1791	1519	121.001	1519	87.93858
1518	167.1811	1518	138.2372	1518	121.0617	1518	87.98619
1517	167.1991	1517	138.3354	1517	121.0821	1517	88.01649
1516	167.2036	1516	138.4625	1516	121.0777	1516	88.03809
1515	167.1882	1515	138.5126	1515	121.0182	1515	88.03967
1514	167.1673	1514	138.4994	1514	120.9316	1514	88.03191
1513	167.1571	1513	138.5011	1513	120.8797	1513	88.04218
1512	167.1737	1512	138.581	1512	120.8988	1512	88.09403
1511	167.2171	1511	138.7199	1511	120.9901	1511	88.18053
1510	167.2792	1510	138.868	1510	121.1192	1510	88.27463
1509	167.3251	1509	138.9618	1509	121.195	1509	88.3279
1508	167.3297	1508	139.0795	1508	121.1523	1508	88.31097
1507	167.3602	1507	139.4772	1507	121.3632	1507	88.36311
1506	167.3535	1506	139.7888	1506	121.5738	1506	88.44304
1505	167.2609	1505	139.7234	1505	121.2731	1505	88.39788
1504	167.2096	1504	139.6425	1504	121.0277	1504	88.34688
1503	167.2157	1503	139.6502	1503	120.9422	1503	88.34799
1502	167.2333	1502	139.6926	1502	120.9312	1502	88.36634
1501	167.2543	1501	139.7792	1501	120.939	1501	88.40004
1500	167.2676	1500	139.8874	1500	120.9499	1500	88.42949
1499	167.2783	1499	139.9857	1499	120.9515	1499	88.4531
1498	167.2774	1498	140.0427	1498	120.9257	1498	88.45484
1497	167.2795	1497	140.1069	1497	120.9199	1497	88.45241
1496	167.3001	1496	140.2067	1496	120.9957	1496	88.47603
1495	167.329	1495	140.3119	1495	121.0829	1495	88.51819
1494	167.3549	1494	140.408	1494	121.1274	1494	88.55489
1493	167.3774	1493	140.4979	1493	121.154	1493	88.58342
1492	167.3986	1492	140.5788	1492	121.1849	1492	88.61276
1491	167.4126	1491	140.6451	1491	121.1793	1491	88.63097
1490	167.4147	1490	140.7119	1490	121.1162	1490	88.61592
1489	167.4169	1489	140.7957	1489	121.1208	1489	88.58641
1488	167.4111	1488	140.8426	1488	121.1963	1488	88.5537
1487	167.3857	1487	140.8268	1487	121.2401	1487	88.50925
1486	167.3503	1486	140.7894	1486	121.2377	1486	88.45247
1485	167.3075	1485	140.7576	1485	121.2155	1485	88.38037
1484	167.2559	1484	140.7368	1484	121.1986	1484	88.29316

1483	167.1856	1483	140.718	1483	121.1881	1483	88.18028
1482	167.0756	1482	140.6966	1482	121.153	1482	88.01625
1481	166.9205	1481	140.6776	1481	121.0786	1481	87.79846
1480	166.7488	1480	140.667	1480	120.9928	1480	87.56896
1479	166.5971	1479	140.6671	1479	120.9138	1479	87.36231
1478	166.4788	1478	140.6763	1478	120.8043	1478	87.16342
1477	166.3882	1477	140.6783	1477	120.584	1477	86.93181
1476	166.3022	1476	140.6424	1476	120.2241	1476	86.65111
1475	166.1794	1475	140.5491	1475	119.7733	1475	86.33599
1474	165.9576	1474	140.4095	1474	119.2956	1474	85.98032
1473	165.641	1473	140.3098	1473	118.9329	1473	85.61963
1472	165.3759	1472	140.2952	1472	118.7935	1472	85.35597
1471	165.2638	1471	140.2754	1471	118.6403	1471	85.18042
1470	165.2728	1470	140.2231	1470	118.3532	1470	85.01906
1469	165.3478	1469	140.1759	1469	118.0127	1469	84.86161
1468	165.4404	1468	140.1404	1468	117.7042	1468	84.735
1467	165.5107	1467	140.1055	1467	117.484	1467	84.68263
1466	165.5426	1466	140.0549	1466	117.4032	1466	84.76134
1465	165.5602	1465	140.0012	1465	117.5229	1465	85.00696
1464	165.5976	1464	139.9613	1464	117.807	1464	85.33795
1463	165.6453	1463	139.931	1463	118.1155	1463	85.61714
1462	165.69	1462	139.9251	1462	118.4035	1462	85.8199
1461	165.7282	1461	139.9449	1461	118.6965	1461	85.98607
1460	165.7556	1460	139.9547	1460	118.962	1460	86.12969
1459	165.7457	1459	139.8709	1459	119.0424	1459	86.21053
1458	165.7064	1458	139.6598	1458	118.7519	1458	86.19041
1457	165.7617	1457	139.5019	1457	118.4154	1457	86.18656
1456	165.9154	1456	139.4915	1456	118.4146	1456	86.27751
1455	166.0454	1455	139.5214	1455	118.5469	1455	86.37311
1454	166.1333	1454	139.5363	1454	118.6842	1454	86.44809
1453	166.2051	1453	139.5292	1453	118.8237	1453	86.53655
1452	166.2695	1452	139.5187	1452	118.9736	1452	86.64521
1451	166.3291	1451	139.5303	1451	119.1247	1451	86.76254
1450	166.3789	1450	139.5535	1450	119.2594	1450	86.86861
1449	166.4243	1449	139.5816	1449	119.3803	1449	86.9691
1448	166.4668	1448	139.6187	1448	119.5052	1448	87.07184
1447	166.5014	1447	139.6552	1447	119.629	1447	87.16701
1446	166.5235	1446	139.667	1446	119.7325	1446	87.24053
1445	166.5394	1445	139.6648	1445	119.8344	1445	87.30008
1444	166.551	1444	139.6664	1444	119.9452	1444	87.35081
1443	166.551	1443	139.6752	1443	120.0474	1443	87.38811
1442	166.5375	1442	139.6927	1442	120.1277	1442	87.41151
1441	166.5145	1441	139.7212	1441	120.1898	1441	87.4265
1440	166.4813	1440	139.7485	1440	120.2346	1440	87.43242
1439	166.4316	1439	139.7531	1439	120.2524	1439	87.42645

1438	166.3692	1438	139.7338	1438	120.2404	1438	87.41453
1437	166.3244	1437	139.7328	1437	120.2285	1437	87.41618
1436	166.3319	1436	139.7875	1436	120.278	1436	87.45269
1435	166.3796	1435	139.864	1435	120.3731	1435	87.50888
1434	166.4345	1434	139.9228	1434	120.4642	1434	87.55812
1433	166.4843	1433	139.9671	1433	120.5467	1433	87.60115
1432	166.5274	1432	140.0162	1432	120.6321	1432	87.64341
1431	166.5603	1431	140.0774	1431	120.7144	1431	87.67598
1430	166.5733	1430	140.1307	1430	120.7694	1430	87.68115
1429	166.56	1429	140.1582	1429	120.7864	1429	87.6526
1428	166.5254	1428	140.1691	1428	120.7906	1428	87.60343
1427	166.4768	1427	140.1817	1427	120.8202	1427	87.54555
1426	166.4032	1426	140.1953	1426	120.8783	1426	87.4674
1425	166.2683	1425	140.2029	1425	120.9212	1425	87.33344
1424	166.056	1424	140.2098	1424	120.9141	1424	87.13206
1423	165.8076	1423	140.2138	1423	120.8801	1423	86.91185
1422	165.5736	1422	140.1963	1422	120.8525	1422	86.7235
1421	165.3736	1421	140.1578	1421	120.852	1421	86.58654
1420	165.2294	1420	140.1218	1420	120.8664	1420	86.51053
1419	165.1785	1419	140.1096	1419	120.8594	1419	86.50137
1418	165.2122	1418	140.1149	1418	120.8302	1418	86.53974
1417	165.2728	1417	140.1074	1417	120.7772	1417	86.58103
1416	165.3374	1416	140.0799	1416	120.7138	1416	86.61552
1415	165.419	1415	140.0437	1415	120.6581	1415	86.66663
1414	165.5272	1414	140.0069	1414	120.615	1414	86.74797
1413	165.6685	1413	139.9763	1413	120.5835	1413	86.86256
1412	165.8402	1412	139.9579	1412	120.5735	1412	87.00555
1411	166.0209	1411	139.9557	1411	120.6031	1411	87.16108
1410	166.1753	1410	139.9628	1410	120.6639	1410	87.29988
1409	166.2865	1409	139.9719	1409	120.7318	1409	87.40289
1408	166.3611	1408	139.9884	1408	120.805	1408	87.47331
1407	166.4054	1407	140.0168	1407	120.8863	1407	87.51463
1406	166.4102	1406	140.0464	1406	120.9504	1406	87.51321
1405	166.3634	1405	140.0687	1405	120.9704	1405	87.45633
1404	166.2713	1404	140.087	1404	120.9582	1404	87.35631
1403	166.1527	1403	140.1033	1403	120.9453	1403	87.23834
1402	166.0188	1402	140.1175	1402	120.9505	1402	87.11512
1401	165.8677	1401	140.1301	1401	120.9698	1401	86.98499
1400	165.7012	1400	140.1472	1400	120.9813	1400	86.8475
1399	165.5462	1399	140.1787	1399	120.9845	1399	86.72608
1398	165.4363	1398	140.2151	1398	120.992	1398	86.64933
1397	165.3819	1397	140.234	1397	120.9923	1397	86.62147
1396	165.3899	1396	140.2383	1396	120.9864	1396	86.64986
1395	165.4652	1395	140.2488	1395	121.0041	1395	86.74381
1394	165.5711	1394	140.2584	1394	121.035	1394	86.86584

1393	165.6656	1393	140.2546	1393	121.041	1393	86.96825
1392	165.7487	1392	140.2531	1392	121.0409	1392	87.05104
1391	165.8298	1391	140.2612	1391	121.0563	1391	87.1298
1390	165.9074	1390	140.2676	1390	121.0789	1390	87.20575
1389	165.9819	1389	140.2634	1389	121.0874	1389	87.27389
1388	166.0626	1388	140.2594	1388	121.08	1388	87.33561
1387	166.1496	1387	140.2796	1387	121.0949	1387	87.39534
1386	166.2185	1386	140.318	1386	121.1411	1386	87.43901
1385	166.2493	1385	140.349	1385	121.183	1385	87.44789
1384	166.2436	1384	140.3648	1384	121.2063	1384	87.42022
1383	166.2022	1383	140.3722	1383	121.2158	1383	87.35788
1382	166.1175	1382	140.3772	1382	121.2147	1382	87.25549
1381	165.9808	1381	140.3805	1381	121.2061	1381	87.10654
1380	165.7882	1380	140.3829	1380	121.1931	1380	86.91027
1379	165.5503	1379	140.3933	1379	121.1815	1379	86.68016
1378	165.2958	1378	140.42	1378	121.1808	1378	86.4445
1377	165.0543	1377	140.4603	1377	121.1941	1377	86.22869
1376	164.8394	1376	140.5028	1376	121.2088	1376	86.03814
1375	164.656	1375	140.5413	1375	121.2116	1375	85.86538
1374	164.5055	1374	140.576	1374	121.2119	1374	85.70514
1373	164.3693	1373	140.5943	1373	121.2007	1373	85.54628
1372	164.2212	1372	140.5918	1372	121.1673	1372	85.37513
1371	164.0542	1371	140.5905	1371	121.1454	1371	85.18974
1370	163.873	1370	140.606	1370	121.1585	1370	84.98978
1369	163.6912	1369	140.6345	1369	121.1836	1369	84.78043
1368	163.539	1368	140.6666	1368	121.1866	1368	84.59052
1367	163.446	1367	140.6994	1367	121.1612	1367	84.45518
1366	163.4243	1366	140.7392	1366	121.1208	1366	84.38373
1365	163.4755	1365	140.7877	1365	121.037	1365	84.35213
1364	163.5998	1364	140.8305	1364	120.7918	1364	84.3071
1363	163.7896	1363	140.8553	1363	120.2756	1363	84.19618
1362	164.0086	1362	140.8651	1362	119.5888	1362	84.03555
1361	164.205	1361	140.8603	1361	118.9821	1361	83.92362
1360	164.3658	1360	140.8453	1360	118.6413	1360	83.97138
1359	164.5094	1359	140.8341	1359	118.6341	1359	84.23073
1358	164.645	1358	140.8327	1358	118.9179	1358	84.65929
1357	164.7674	1357	140.8356	1357	119.356	1357	85.14214
1356	164.8736	1356	140.8355	1356	119.7893	1356	85.56733
1355	164.9689	1355	140.8296	1355	120.1203	1355	85.88314
1354	165.0599	1354	140.8197	1354	120.3271	1354	86.08987
1353	165.1506	1353	140.8064	1353	120.4214	1353	86.207
1352	165.2444	1352	140.7868	1352	120.4193	1352	86.25864
1351	165.3452	1351	140.7605	1351	120.3456	1351	86.26862
1350	165.4544	1350	140.7327	1350	120.2372	1350	86.24815
1349	165.5674	1349	140.7074	1349	120.1029	1349	86.17672

1348	165.6773	1348	140.6818	1348	119.8834	1348	85.97952
1347	165.7805	1347	140.6512	1347	119.4433	1347	85.51831
1346	165.8762	1346	140.6154	1346	118.6159	1346	84.65846
1345	165.9637	1345	140.5787	1345	117.3252	1345	83.44059
1344	166.0434	1344	140.545	1344	115.7313	1344	82.19404
1343	166.1164	1343	140.5151	1343	114.2172	1343	81.40238
1342	166.1835	1342	140.484	1342	113.2189	1342	81.43632
1341	166.244	1341	140.445	1341	113.0715	1341	82.37264
1340	166.3033	1340	140.4072	1340	113.867	1340	83.89226
1339	166.3662	1339	140.3895	1339	115.2713	1339	85.37615
1338	166.4208	1338	140.3849	1338	116.6826	1338	86.37301
1337	166.4543	1337	140.3723	1337	117.7432	1337	86.89003
1336	166.472	1336	140.3494	1336	118.4512	1336	87.13957
1335	166.4833	1335	140.3277	1335	118.9183	1335	87.27187
1334	166.4903	1334	140.315	1334	119.2386	1334	87.35279
1333	166.4912	1333	140.3116	1333	119.474	1333	87.40847
1332	166.4856	1332	140.3133	1332	119.6518	1332	87.44516
1331	166.4745	1331	140.3187	1331	119.7784	1331	87.46001
1330	166.4583	1330	140.3292	1330	119.8679	1330	87.45687
1329	166.4368	1329	140.3433	1329	119.9418	1329	87.44548
1328	166.4118	1328	140.3565	1328	120.0079	1328	87.43056
1327	166.3871	1327	140.3673	1327	120.0623	1327	87.41351
1326	166.3651	1326	140.3783	1326	120.1096	1326	87.39968
1325	166.3452	1325	140.3902	1325	120.1557	1325	87.39392
1324	166.3287	1324	140.4013	1324	120.1971	1324	87.3948
1323	166.3185	1323	140.41	1323	120.2272	1323	87.39786
1322	166.3142	1322	140.4155	1322	120.2483	1322	87.40111
1321	166.3121	1321	140.4172	1321	120.269	1321	87.40315
1320	166.3105	1320	140.4166	1320	120.2973	1320	87.40093
1319	166.3108	1319	140.4188	1319	120.3422	1319	87.39438
1318	166.3119	1318	140.4264	1318	120.4078	1318	87.38743
1317	166.3086	1317	140.4345	1317	120.4802	1317	87.37962
1316	166.2971	1316	140.4377	1316	120.5369	1316	87.36333
1315	166.2777	1315	140.4374	1315	120.5697	1315	87.33188
1314	166.2511	1314	140.4398	1314	120.5923	1314	87.28647
1313	166.2175	1313	140.4496	1313	120.6245	1313	87.23388
1312	166.1782	1312	140.4643	1312	120.6667	1312	87.17792
1311	166.1327	1311	140.4773	1311	120.7021	1311	87.11677
1310	166.0772	1310	140.4859	1310	120.7218	1310	87.04718
1309	166.0073	1309	140.4925	1309	120.7311	1309	86.96615
1308	165.9224	1308	140.4992	1308	120.7355	1308	86.87125
1307	165.8264	1307	140.5054	1307	120.735	1307	86.76421
1306	165.7232	1306	140.5093	1306	120.7303	1306	86.65066
1305	165.6124	1305	140.5109	1305	120.7213	1305	86.53225
1304	165.4886	1304	140.5128	1304	120.7058	1304	86.40186

1303	165.3391	1303	140.5173	1303	120.6812	1303	86.24597
1302	165.144	1302	140.5232	1302	120.6488	1302	86.05004
1301	164.8844	1301	140.5265	1301	120.6111	1301	85.8022
1300	164.5549	1300	140.5234	1300	120.5687	1300	85.49645
1299	164.1657	1299	140.5134	1299	120.5228	1299	85.13547
1298	163.7389	1298	140.4993	1298	120.4806	1298	84.73247
1297	163.3112	1297	140.4865	1297	120.4519	1297	84.31521
1296	162.941	1296	140.4784	1296	120.4381	1296	83.93269
1295	162.6992	1295	140.4746	1295	120.4311	1295	83.65109
1294	162.6377	1294	140.4716	1294	120.4183	1294	83.52764
1293	162.7656	1293	140.4666	1293	120.3862	1293	83.57979
1292	163.0478	1292	140.4597	1292	120.3195	1292	83.77357
1291	163.4195	1291	140.4514	1291	120.2009	1291	84.03525
1290	163.809	1290	140.4401	1290	120.0165	1290	84.27968
1289	164.1631	1289	140.4243	1289	119.7618	1289	84.44152
1288	164.4567	1288	140.406	1288	119.445	1288	84.49221
1287	164.6845	1287	140.3883	1287	119.0846	1287	84.43697
1286	164.8499	1286	140.3718	1286	118.6958	1286	84.29844
1285	164.9638	1285	140.355	1285	118.2811	1285	84.10049
1284	165.0436	1284	140.3376	1284	117.8386	1284	83.8644
1283	165.1056	1283	140.3217	1283	117.3791	1283	83.61594
1282	165.1578	1282	140.3096	1282	116.9354	1282	83.39185
1281	165.2021	1281	140.3015	1281	116.5576	1281	83.23766
1280	165.2408	1280	140.2949	1280	116.3014	1280	83.19629
1279	165.2766	1279	140.2868	1279	116.211	1279	83.29023
1278	165.3098	1278	140.2757	1278	116.2974	1278	83.50839
1277	165.3395	1277	140.2632	1277	116.5333	1277	83.81342
1276	165.3669	1276	140.2517	1276	116.8722	1276	84.16246
1275	165.394	1275	140.2421	1275	117.2708	1275	84.5209
1274	165.4203	1274	140.2317	1274	117.6954	1274	84.86413
1273	165.4441	1273	140.2176	1273	118.1194	1273	85.17558
1272	165.4654	1272	140.2008	1272	118.5209	1272	85.4444
1271	165.4841	1271	140.1834	1271	118.8839	1271	85.66345
1270	165.497	1270	140.1648	1270	119.1985	1270	85.83011
1269	165.5002	1269	140.1411	1269	119.4598	1269	85.94829
1268	165.4943	1268	140.112	1268	119.669	1268	86.02709
1267	165.4815	1267	140.0821	1267	119.8352	1267	86.07542
1266	165.4608	1266	140.0553	1266	119.9714	1266	86.09843
1265	165.4299	1265	140.0305	1265	120.0858	1265	86.09938
1264	165.3894	1264	140.0045	1264	120.1792	1264	86.08265
1263	165.3422	1263	139.9752	1263	120.2507	1263	86.05292
1262	165.2884	1262	139.9435	1262	120.3046	1262	86.01229
1261	165.2267	1261	139.9118	1261	120.3507	1261	85.96041
1260	165.1587	1260	139.8829	1260	120.397	1260	85.89739
1259	165.0852	1259	139.8566	1259	120.446	1259	85.82404

1258	165.0013	1258	139.8286	1258	120.4942	1258	85.73843
1257	164.8976	1257	139.7948	1257	120.5365	1257	85.63595
1256	164.7678	1256	139.7567	1256	120.5699	1256	85.51284
1255	164.6064	1255	139.7193	1255	120.5953	1255	85.36552
1254	164.3998	1254	139.6848	1254	120.6144	1254	85.18424
1253	164.1225	1253	139.6506	1253	120.6256	1253	84.9493
1252	163.7443	1252	139.6146	1252	120.6232	1252	84.63467
1251	163.2388	1251	139.5775	1251	120.6025	1251	84.21287
1250	162.5934	1250	139.54	1250	120.5604	1250	83.65647
1249	161.8248	1249	139.5011	1249	120.4897	1249	82.94515
1248	160.9935	1248	139.4614	1248	120.3734	1248	82.08334
1247	160.1946	1247	139.4245	1247	120.1831	1247	81.11232
1246	159.5183	1246	139.3941	1246	119.8838	1246	80.09985
1245	159.0167	1245	139.3721	1245	119.4549	1245	79.12409
1244	158.7007	1244	139.3601	1244	118.9281	1244	78.27366
1243	158.5509	1243	139.3582	1243	118.4093	1243	77.64346
1242	158.5258	1242	139.3633	1242	118.0385	1242	77.30306
1241	158.5851	1241	139.3716	1241	117.9065	1241	77.26813
1240	158.7167	1240	139.3842	1240	118.0002	1240	77.5051
1239	158.9321	1239	139.405	1239	118.2169	1239	77.94964
1238	159.2362	1238	139.4344	1238	118.4343	1238	78.52589
1237	159.6145	1237	139.4686	1237	118.5848	1237	79.17463
1236	160.0465	1236	139.5054	1236	118.6792	1236	79.87058
1235	160.5121	1235	139.5462	1235	118.7698	1235	80.60334
1234	160.985	1234	139.5914	1234	118.9001	1234	81.34877
1233	161.4373	1233	139.6386	1233	119.0851	1233	82.07151
1232	161.854	1232	139.6862	1232	119.3218	1232	82.74613
1231	162.2317	1231	139.7347	1231	119.5978	1231	83.35933
1230	162.567	1230	139.7841	1230	119.8876	1230	83.89713
1229	162.8554	1229	139.832	1229	120.1551	1229	84.34513
1228	163.1002	1228	139.8766	1228	120.3723	1228	84.70094
1227	163.3121	1227	139.9191	1227	120.5339	1227	84.97738
1226	163.4987	1226	139.9635	1226	120.6529	1226	85.19324
1225	163.6603	1225	140.013	1225	120.746	1225	85.36469
1224	163.7965	1224	140.0675	1224	120.822	1224	85.50324
1223	163.9093	1223	140.1225	1223	120.8833	1223	85.61577
1222	164.0022	1222	140.1727	1222	120.9312	1222	85.70484
1221	164.0802	1221	140.2174	1221	120.9682	1221	85.77219
1220	164.1505	1220	140.261	1220	120.9981	1220	85.82303
1219	164.2178	1219	140.3067	1219	121.0241	1219	85.86463
1218	164.2784	1218	140.3517	1218	121.0465	1218	85.9001
1217	164.3252	1217	140.391	1217	121.062	1217	85.92641
1216	164.3577	1216	140.4229	1216	121.0668	1216	85.93931
1215	164.3816	1215	140.4488	1215	121.0615	1215	85.93792
1214	164.3994	1214	140.4692	1214	121.05	1214	85.92432

1213	164.4079	1213	140.4836	1213	121.0365	1213	85.90122
1212	164.4048	1212	140.493	1212	121.0236	1212	85.87086
1211	164.3913	1211	140.4993	1211	121.0126	1211	85.83365
1210	164.3672	1210	140.5026	1210	121.0026	1210	85.78702
1209	164.3297	1209	140.5032	1209	120.9918	1209	85.72696
1208	164.2756	1208	140.503	1208	120.9797	1208	85.65106
1207	164.2025	1207	140.5039	1207	120.9684	1207	85.55872
1206	164.1044	1206	140.5051	1206	120.9602	1206	85.44842
1205	163.9713	1205	140.5045	1205	120.9561	1205	85.31632
1204	163.7908	1204	140.5027	1204	120.9557	1204	85.15673
1203	163.5456	1203	140.5017	1203	120.9581	1203	84.96111
1202	163.2096	1202	140.5022	1202	120.9608	1202	84.71583
1201	162.7507	1201	140.5037	1201	120.9607	1201	84.40332
1200	162.1446	1200	140.5069	1200	120.9584	1200	84.007
1199	161.3867	1199	140.512	1199	120.9596	1199	83.51378
1198	160.4974	1198	140.5157	1198	120.9691	1198	82.91192
1197	159.5229	1197	140.5142	1197	120.9851	1197	82.1925
1196	158.5325	1196	140.5077	1196	121.0024	1196	81.35877
1195	157.6041	1195	140.5009	1195	121.0178	1195	80.43375
1194	156.7972	1194	140.4973	1194	121.0307	1194	79.45724
1193	156.1417	1193	140.4967	1193	121.0401	1193	78.48166
1192	155.6524	1192	140.4982	1192	121.0449	1192	77.57429
1191	155.3361	1191	140.5018	1191	121.0462	1191	76.80762
1190	155.1787	1190	140.5071	1190	121.0449	1190	76.23039
1189	155.146	1189	140.5134	1189	121.0398	1189	75.85321
1188	155.2074	1188	140.5204	1188	121.03	1188	75.66562
1187	155.3423	1187	140.5279	1187	121.0191	1187	75.64761
1186	155.5207	1186	140.5345	1186	121.0118	1186	75.75783
1185	155.7012	1185	140.5389	1185	121.0068	1185	75.93361
1184	155.8517	1184	140.5416	1184	120.9977	1184	76.11642
1183	155.9593	1183	140.5428	1183	120.9795	1183	76.26991
1182	156.0211	1182	140.5401	1182	120.9531	1182	76.37761
1181	156.0406	1181	140.5307	1181	120.9211	1181	76.44186
1180	156.0361	1180	140.5161	1180	120.885	1180	76.48543
1179	156.0315	1179	140.5007	1179	120.8467	1179	76.53555
1178	156.0349	1178	140.4859	1178	120.8082	1178	76.59919
1177	156.0377	1177	140.4694	1177	120.7693	1177	76.6648
1176	156.0356	1176	140.4502	1176	120.7285	1176	76.72725
1175	156.0347	1175	140.4304	1175	120.6862	1175	76.79528
1174	156.0349	1174	140.4123	1174	120.6453	1174	76.87468
1173	156.0266	1173	140.3958	1173	120.6056	1173	76.9623
1172	156.0087	1172	140.3815	1172	120.5641	1172	77.06013
1171	155.9915	1171	140.3721	1171	120.5202	1171	77.17747
1170	155.9791	1170	140.3677	1170	120.4757	1170	77.31462
1169	155.9643	1169	140.3641	1169	120.4305	1169	77.46067

1168	155.9455	1168	140.357	1168	120.3815	1168	77.61157
1167	155.9325	1167	140.3469	1167	120.3273	1167	77.77615
1166	155.9309	1166	140.3376	1166	120.2698	1166	77.96083
1165	155.9391	1165	140.3323	1165	120.2089	1165	78.16313
1164	155.9623	1164	140.333	1164	120.1414	1164	78.38252
1163	156.015	1163	140.341	1163	120.0634	1163	78.62294
1162	156.1025	1162	140.3561	1162	119.9723	1162	78.88119
1161	156.2188	1161	140.3766	1161	119.8636	1161	79.146
1160	156.3647	1160	140.401	1160	119.7296	1160	79.40976
1159	156.5524	1159	140.4297	1159	119.5624	1159	79.66728
1158	156.7867	1158	140.4617	1158	119.3516	1158	79.90064
1157	157.0586	1157	140.4944	1157	119.0787	1157	80.07759
1156	157.361	1156	140.5254	1156	118.7172	1156	80.16613
1155	157.694	1155	140.5552	1155	118.243	1155	80.14271
1154	158.0521	1154	140.5845	1154	117.6495	1154	79.9957
1153	158.4224	1153	140.613	1153	116.9647	1153	79.74534
1152	158.7996	1152	140.6408	1152	116.2627	1152	79.4677
1151	159.1899	1151	140.67	1151	115.65	1151	79.27772
1150	159.5949	1150	140.7025	1150	115.2156	1150	79.2644
1149	160.0053	1149	140.7373	1149	114.9806	1149	79.43762
1148	160.4099	1148	140.7715	1148	114.8983	1148	79.73634
1147	160.8026	1147	140.8037	1147	114.8963	1147	80.07839
1146	161.1775	1146	140.8354	1146	114.9237	1146	80.40922
1145	161.5288	1145	140.8674	1145	114.9738	1145	80.72924
1144	161.8562	1144	140.8991	1144	115.0813	1144	81.08605
1143	162.1627	1143	140.9293	1143	115.2913	1143	81.52897
1142	162.4474	1142	140.9571	1142	115.6177	1142	82.05917
1141	162.7058	1141	140.9814	1141	116.0247	1141	82.62413
1140	162.9388	1140	141.0006	1140	116.4496	1140	83.15644
1139	163.1531	1139	141.0145	1139	116.8377	1139	83.60956
1138	163.3537	1138	141.026	1138	117.1586	1138	83.96348
1137	163.542	1137	141.0389	1137	117.4006	1137	84.21459
1136	163.7187	1136	141.0534	1136	117.5617	1136	84.36851
1135	163.8834	1135	141.0654	1135	117.6464	1135	84.43689
1134	164.0324	1134	141.0714	1134	117.6634	1134	84.43216
1133	164.1628	1133	141.0727	1133	117.6198	1133	84.36042
1132	164.278	1132	141.0729	1132	117.5127	1132	84.21524
1131	164.3842	1131	141.073	1131	117.327	1131	83.9734
1130	164.4828	1130	141.0698	1130	117.0379	1130	83.5954
1129	164.5721	1129	141.0604	1129	116.6202	1129	83.03679
1128	164.6539	1128	141.0462	1128	116.0616	1128	82.26834
1127	164.732	1127	141.0315	1127	115.3729	1127	81.29415
1126	164.8063	1126	141.0193	1126	114.5879	1126	80.15889
1125	164.8734	1125	141.0095	1125	113.7588	1125	78.94427
1124	164.9306	1124	141.0007	1124	112.9491	1124	77.75314

1123	164.9747	1123	140.9906	1123	112.2155	1123	76.6696
1122	164.9981	1122	140.9759	1122	111.5733	1122	75.7074
1121	164.9932	1121	140.9547	1121	110.9835	1121	74.80231
1120	164.9607	1120	140.9288	1120	110.3837	1120	73.8698
1119	164.9063	1119	140.9028	1119	109.7308	1119	72.86614
1118	164.8292	1118	140.8793	1118	109.0112	1118	71.79777
1117	164.7192	1117	140.856	1117	108.2354	1117	70.70248
1116	164.5661	1116	140.8281	1116	107.4394	1116	69.63935
1115	164.3681	1115	140.7931	1115	106.6776	1115	68.66964
1114	164.1292	1114	140.7536	1114	105.9892	1114	67.81584
1113	163.8576	1113	140.7153	1113	105.3739	1113	67.05027
1112	163.5694	1112	140.684	1112	104.8083	1112	66.34124
1111	163.2905	1111	140.6626	1111	104.274	1111	65.69796
1110	163.0498	1110	140.6488	1110	103.7598	1110	65.16556
1109	162.8716	1109	140.638	1109	103.2601	1109	64.80553
1108	162.7717	1108	140.6276	1108	102.7883	1108	64.69172
1107	162.7512	1107	140.6185	1107	102.3692	1107	64.88794
1106	162.7908	1106	140.612	1106	102.0035	1106	65.40598
1105	162.8613	1105	140.6069	1105	101.6595	1105	66.20756
1104	162.9416	1104	140.6001	1104	101.3105	1104	67.2517
1103	163.0225	1103	140.5886	1103	100.9618	1103	68.50827
1102	163.0961	1102	140.5719	1102	100.6324	1102	69.92451
1101	163.1545	1101	140.5549	1101	100.338	1101	71.41977
1100	163.1988	1100	140.5461	1100	100.113	1100	72.9205
1099	163.2363	1099	140.548	1099	100.0064	1099	74.37253
1098	163.2702	1098	140.555	1098	100.0491	1098	75.72714
1097	163.2973	1097	140.5583	1097	100.2336	1097	76.94178
1096	163.3183	1096	140.5553	1096	100.5428	1096	78.00201
1095	163.3409	1095	140.5496	1095	100.9692	1095	78.92225
1094	163.372	1094	140.5445	1094	101.4957	1094	79.72182
1093	163.4121	1093	140.5387	1093	102.0843	1093	80.41153
1092	163.4614	1092	140.53	1092	102.7014	1092	81.00211
1091	163.5227	1091	140.5177	1091	103.3304	1091	81.51073
1090	163.596	1090	140.5004	1090	103.9551	1090	81.95504
1089	163.6773	1089	140.476	1089	104.5539	1089	82.3494
1088	163.7648	1088	140.4445	1088	105.121	1088	82.70843
1087	163.8595	1087	140.4091	1087	105.6716	1087	83.04515
1086	163.9588	1086	140.3722	1086	106.2169	1086	83.36295
1085	164.0571	1085	140.334	1085	106.7527	1085	83.65656
1084	164.1511	1084	140.2962	1084	107.2773	1084	83.92332
1083	164.2408	1083	140.2635	1083	107.7996	1083	84.16735
1082	164.3247	1082	140.2386	1082	108.3216	1082	84.39219
1081	164.4008	1081	140.2209	1081	108.8303	1081	84.59647
1080	164.4712	1080	140.2091	1080	109.3147	1080	84.77864
1079	164.5389	1079	140.2042	1079	109.7763	1079	84.93884

1078	164.601	1078	140.2063	1078	110.2143	1078	85.07382
1077	164.6501	1077	140.2127	1077	110.6163	1077	85.17611
1076	164.6832	1076	140.2221	1076	110.9734	1076	85.2417
1075	164.7001	1075	140.2357	1075	111.2915	1075	85.2731
1074	164.6956	1074	140.2522	1074	111.5809	1074	85.27178
1073	164.6607	1073	140.2656	1073	111.8445	1073	85.23232
1072	164.5919	1072	140.271	1072	112.0824	1072	85.14828
1071	164.4939	1071	140.2711	1071	112.2957	1071	85.01912
1070	164.3711	1070	140.2726	1070	112.4766	1070	84.84721
1069	164.2267	1069	140.2781	1069	112.6049	1069	84.63251
1068	164.0743	1068	140.2853	1068	112.6591	1068	84.37431
1067	163.9422	1067	140.2915	1067	112.6173	1067	84.07072
1066	163.8606	1066	140.2948	1066	112.4376	1066	83.7091
1065	163.8436	1065	140.2931	1065	112.0593	1065	83.26995
1064	163.887	1064	140.2866	1064	111.4552	1064	82.76479
1063	163.9738	1063	140.2803	1063	110.6959	1063	82.27715
1062	164.0805	1062	140.282	1062	109.9512	1062	81.9476
1061	164.1851	1061	140.2946	1061	109.4142	1061	81.8967
1060	164.2745	1060	140.3142	1060	109.216	1060	82.14868
1059	164.3423	1059	140.3346	1059	109.3797	1059	82.61458
1058	164.378	1058	140.3519	1058	109.8255	1058	83.14191
1057	164.3656	1057	140.3652	1057	110.4211	1057	83.59216
1056	164.2918	1056	140.3755	1056	111.0483	1056	83.8888
1055	164.1507	1055	140.3835	1055	111.6367	1055	84.01452
1054	163.9404	1054	140.3887	1054	112.1561	1054	83.98536
1053	163.6576	1053	140.3891	1053	112.599	1053	83.83224
1052	163.3016	1052	140.3832	1052	112.9726	1052	83.59352
1051	162.8818	1051	140.3717	1051	113.2912	1051	83.30957
1050	162.4244	1050	140.3575	1050	113.5641	1050	83.01867
1049	161.978	1049	140.3433	1049	113.7927	1049	82.7616
1048	161.6097	1048	140.3322	1048	113.9819	1048	82.58701
1047	161.3813	1047	140.3271	1047	114.1447	1047	82.53864
1046	161.3167	1046	140.3294	1046	114.2925	1046	82.63085
1045	161.3967	1045	140.3377	1045	114.4265	1045	82.84362
1044	161.5821	1044	140.3494	1044	114.5461	1044	83.14118
1043	161.8336	1043	140.3625	1043	114.6572	1043	83.48754
1042	162.1138	1042	140.375	1042	114.7659	1042	83.84878
1041	162.3907	1041	140.3838	1041	114.8707	1041	84.19575
1040	162.6469	1040	140.385	1040	114.967	1040	84.51041
1039	162.8785	1039	140.3763	1039	115.0561	1039	84.7864
1038	163.0844	1038	140.3586	1038	115.1428	1038	85.02352
1037	163.2645	1037	140.3361	1037	115.2274	1037	85.22501
1036	163.4252	1036	140.3157	1036	115.3073	1036	85.39855
1035	163.578	1035	140.303	1035	115.3854	1035	85.55402
1034	163.7263	1034	140.2988	1034	115.4712	1034	85.6982

1033	163.863	1033	140.298	1033	115.5722	1033	85.83329
1032	163.9817	1032	140.2953	1032	115.6868	1032	85.9596
1031	164.0851	1031	140.2899	1031	115.8053	1031	86.07621
1030	164.1788	1030	140.2842	1030	115.9156	1030	86.18007
1029	164.2633	1029	140.2807	1029	116.0126	1029	86.2692
1028	164.3377	1028	140.2818	1028	116.1021	1028	86.34748
1027	164.4043	1027	140.2902	1027	116.1943	1027	86.42252
1026	164.4666	1026	140.307	1026	116.2909	1026	86.49721
1025	164.5242	1025	140.3293	1025	116.3825	1025	86.56635
1024	164.5732	1024	140.3512	1024	116.4606	1024	86.62282
1023	164.6112	1023	140.3672	1023	116.5271	1023	86.66455
1022	164.6395	1022	140.3751	1022	116.5898	1022	86.69466
1021	164.6622	1021	140.3778	1021	116.652	1021	86.71804
1020	164.6858	1020	140.3823	1020	116.7128	1020	86.74047
1019	164.7149	1019	140.3958	1019	116.7734	1019	86.76861
1018	164.7495	1018	140.4209	1018	116.8368	1018	86.80695
1017	164.7854	1017	140.4538	1017	116.904	1017	86.85448
1016	164.8207	1016	140.4895	1016	116.9748	1016	86.90631
1015	164.8578	1015	140.5278	1015	117.0513	1015	86.9588
1014	164.8994	1014	140.5723	1014	117.1371	1014	87.01231
1013	164.9455	1013	140.6253	1013	117.233	1013	87.06916
1012	164.9951	1012	140.6849	1012	117.3354	1012	87.12969
1011	165.0472	1011	140.7462	1011	117.4406	1011	87.19062
1010	165.0983	1010	140.8032	1010	117.546	1010	87.24709
1009	165.1432	1009	140.8518	1009	117.6505	1009	87.29616
1008	165.179	1008	140.8911	1008	117.755	1008	87.33788
1007	165.2073	1007	140.9248	1007	117.8614	1007	87.37344
1006	165.2335	1006	140.9583	1006	117.9693	1006	87.40374
1005	165.2633	1005	140.9948	1005	118.0727	1005	87.43027
1004	165.2998	1004	141.0322	1004	118.1653	1004	87.45597
1003	165.3403	1003	141.0656	1003	118.2464	1003	87.48344
1002	165.3778	1002	141.0919	1002	118.3211	1002	87.51267
1001	165.4059	1001	141.1132	1001	118.3948	1001	87.54115
1000	165.4248	1000	141.1344	1000	118.4688	1000	87.56586
999	165.4381	999	141.1578	999	118.541	999	87.58424
998	165.4481	998	141.181	998	118.6085	998	87.59498
997	165.4564	997	141.2007	997	118.6707	997	87.60024
996	165.467	996	141.2177	996	118.7309	996	87.60646
995	165.4841	995	141.2366	995	118.7937	995	87.6198
994	165.505	994	141.2602	994	118.8588	994	87.63946
993	165.5211	993	141.2854	993	118.9192	993	87.65716
992	165.5276	992	141.3067	992	118.9685	992	87.66476
991	165.5288	991	141.3225	991	119.0096	991	87.66182
990	165.5321	990	141.3358	990	119.0508	990	87.65522
989	165.5416	989	141.3506	989	119.096	989	87.65286

988	165.5574	988	141.3681	988	119.1429	988	87.65831
987	165.578	987	141.3877	987	119.1892	987	87.66968
986	165.5996	986	141.4079	986	119.2359	986	87.68141
985	165.6178	985	141.4263	985	119.283	985	87.68711
984	165.631	984	141.4398	984	119.3275	984	87.68222
983	165.6405	983	141.446	983	119.3659	983	87.66505
982	165.6449	982	141.4453	982	119.3981	982	87.63592
981	165.6393	981	141.4407	981	119.426	981	87.59581
980	165.6199	980	141.4343	980	119.4513	980	87.54504
979	165.5886	979	141.4267	979	119.4751	979	87.48225
978	165.5486	978	141.4168	978	119.496	978	87.40422
977	165.501	977	141.4047	977	119.508	977	87.30757
976	165.4468	976	141.3921	976	119.503	976	87.19096
975	165.39	975	141.3816	975	119.4767	975	87.0538
974	165.335	974	141.3738	974	119.4259	974	86.89091
973	165.2812	973	141.366	973	119.3389	973	86.68798
972	165.2224	972	141.3545	972	119.1905	972	86.42184
971	165.149	971	141.3384	971	118.9469	971	86.06183
970	165.0501	970	141.3204	970	118.5704	970	85.56867
969	164.9151	969	141.3044	969	118.0193	969	84.89682
968	164.7362	968	141.2927	968	117.2542	968	84.01365
967	164.5093	967	141.2848	967	116.264	967	82.93771
966	164.2334	966	141.2787	966	115.0968	966	81.77407
965	163.9184	965	141.274	965	113.8713	965	80.71298
964	163.598	964	141.2732	964	112.7577	964	79.97505
963	163.3312	963	141.2796	963	111.9261	963	79.71613
962	163.1768	962	141.2945	962	111.4758	962	79.94498
961	163.1608	961	141.3156	961	111.3946	961	80.52526
960	163.2668	960	141.3397	960	111.5853	960	81.2647
959	163.4536	959	141.3641	959	111.9293	959	82.01001
958	163.6796	958	141.3873	958	112.3333	958	82.68424
957	163.9179	957	141.4086	957	112.7443	957	83.27106
956	164.1555	956	141.4291	956	113.1398	956	83.77865
955	164.3838	955	141.4512	955	113.5067	955	84.20985
954	164.5931	954	141.4748	954	113.8221	954	84.55365
953	164.7765	953	141.4957	953	114.0532	953	84.79819
952	164.935	952	141.5101	952	114.1752	952	84.94678
951	165.0739	951	141.5196	951	114.1877	951	85.01947
950	165.1965	950	141.5291	950	114.1161	950	85.04419
949	165.3032	949	141.5415	949	114.0024	949	85.05241
948	165.3953	948	141.5545	948	113.8969	948	85.08009
947	165.4756	947	141.5633	947	113.848	947	85.16013
946	165.5445	946	141.5651	946	113.886	946	85.30598
945	165.5995	945	141.5607	945	114.0145	945	85.50582
944	165.6385	944	141.5535	944	114.219	944	85.735

943	165.6608	943	141.5471	943	114.4799	943	85.97016
942	165.6646	942	141.5437	942	114.7771	942	86.19217
941	165.645	941	141.5422	941	115.0887	941	86.38432
940	165.5949	940	141.5391	940	115.3953	940	86.53313
939	165.5088	939	141.5301	939	115.681	939	86.62973
938	165.3877	938	141.5133	938	115.9313	938	86.67089
937	165.2451	937	141.4909	937	116.1337	937	86.66387
936	165.108	936	141.4704	936	116.285	936	86.63267
935	165.0082	935	141.4589	935	116.3989	935	86.6142
934	164.968	934	141.4566	934	116.501	934	86.64132
933	164.993	933	141.4574	933	116.6159	933	86.72695
932	165.0755	932	141.4577	932	116.7559	932	86.8629
931	165.199	931	141.4613	931	116.9162	931	87.02848
930	165.3413	930	141.4723	930	117.077	930	87.19915
929	165.4808	929	141.4867	929	117.2168	929	87.35454
928	165.6058	928	141.4951	928	117.3303	928	87.48603
927	165.7147	927	141.4936	927	117.4318	927	87.598
926	165.8083	926	141.4852	926	117.5409	926	87.70033
925	165.8855	925	141.4732	925	117.6645	925	87.79944
924	165.9469	924	141.4591	924	117.797	924	87.8955
923	165.9973	923	141.4472	923	117.9306	923	87.98471
922	166.0419	922	141.4447	922	118.0598	922	88.06291
921	166.0824	921	141.4539	921	118.1789	921	88.12917
920	166.1183	920	141.4682	920	118.2829	920	88.18715
919	166.1498	919	141.479	919	118.372	919	88.24115
918	166.1772	918	141.4853	918	118.4509	918	88.29038
917	166.2006	917	141.4935	917	118.5231	917	88.32997
916	166.2217	916	141.5101	916	118.5882	916	88.35859
915	166.2445	915	141.5358	915	118.6464	915	88.3813
914	166.2717	914	141.5642	914	118.699	914	88.40366
913	166.3018	913	141.5866	913	118.7458	913	88.42695
912	166.3316	912	141.5989	912	118.7861	912	88.45162
911	166.3591	911	141.6049	911	118.8225	911	88.48096
910	166.383	910	141.6115	910	118.8598	910	88.51702
909	166.4026	909	141.6205	909	118.8996	909	88.55559
908	166.4207	908	141.6287	908	118.9402	908	88.58889
907	166.4407	907	141.6343	907	118.9817	907	88.61145
906	166.4615	906	141.6377	906	119.0265	906	88.6235
905	166.4769	905	141.634	905	119.0725	905	88.63448
904	166.4856	904	141.6146	904	119.1124	904	88.66383
903	166.4969	903	141.5813	903	119.1422	903	88.72669
902	166.5206	902	141.5542	902	119.1672	902	88.80945
901	166.5534	901	141.5561	901	119.197	901	88.86896
900	166.5804	900	141.5903	900	119.2378	900	88.8697
899	166.5907	899	141.6368	899	119.2881	899	88.8202

898	166.5874	898	141.6715	898	119.3401	898	88.76273
897	166.5805	897	141.6841	897	119.3836	897	88.72836
896	166.5762	896	141.6802	896	119.4134	896	88.71548
895	166.5751	895	141.672	895	119.4341	895	88.7102
894	166.5762	894	141.6688	894	119.4545	894	88.70925
893	166.5797	893	141.6732	893	119.4773	893	88.71577
892	166.586	892	141.6819	892	119.4985	892	88.72635
891	166.5946	891	141.6903	891	119.5164	891	88.73433
890	166.6059	890	141.697	890	119.5348	890	88.74016
889	166.6207	889	141.7043	889	119.5559	889	88.74939
888	166.6396	888	141.7143	888	119.5781	888	88.76328
887	166.661	887	141.7252	887	119.6002	887	88.77735
886	166.6799	886	141.7322	886	119.6226	886	88.78639
885	166.6906	885	141.7306	885	119.6429	885	88.78666
884	166.6917	884	141.719	884	119.6565	884	88.77646
883	166.6877	883	141.7007	883	119.6639	883	88.75905
882	166.6851	882	141.6804	882	119.6721	882	88.742
881	166.6867	881	141.6609	881	119.685	881	88.73009
880	166.6921	880	141.6429	880	119.7009	880	88.72175
879	166.7006	879	141.6283	879	119.7185	879	88.7144
878	166.7099	878	141.6188	878	119.7401	878	88.7082
877	166.7156	877	141.6129	877	119.763	877	88.70206
876	166.715	876	141.6062	876	119.7786	876	88.69198
875	166.7125	875	141.5978	875	119.782	875	88.67728
874	166.7155	874	141.5921	874	119.7763	874	88.66256
873	166.7232	873	141.5933	873	119.7649	873	88.64979
872	166.7269	872	141.5999	872	119.7472	872	88.63482
871	166.7234	871	141.6076	871	119.7264	871	88.61641
870	166.7215	870	141.613	870	119.7118	870	88.60208
869	166.7321	869	141.6141	869	119.7085	869	88.59854
868	166.7528	868	141.6091	868	119.7114	868	88.60074
867	166.7702	867	141.598	867	119.7128	867	88.59617
866	166.7749	866	141.5843	866	119.7114	866	88.5775
865	166.7707	865	141.5735	865	119.7066	865	88.54663
864	166.7682	864	141.5677	864	119.6927	864	88.50865
863	166.7727	863	141.5655	863	119.6624	863	88.46558
862	166.7807	862	141.5631	862	119.6118	862	88.41446
861	166.7858	861	141.5587	861	119.539	861	88.34986
860	166.7869	860	141.5534	860	119.4429	860	88.27069
859	166.7887	859	141.5501	859	119.3312	859	88.18523
858	166.796	858	141.5492	858	119.2208	858	88.10504
857	166.8081	857	141.5468	857	119.1266	857	88.03291
856	166.8196	856	141.5387	856	119.0503	856	87.96135
855	166.8252	855	141.526	855	118.9826	855	87.88173
854	166.8231	854	141.5137	854	118.9069	854	87.78637

853	166.8134	853	141.5034	853	118.7949	853	87.65838
852	166.7982	852	141.4938	852	118.6045	852	87.46721
851	166.7823	851	141.4853	851	118.2897	851	87.17996
850	166.7717	850	141.4824	850	117.814	850	86.7742
849	166.7674	849	141.4879	849	117.1587	849	86.23895
848	166.7622	848	141.4996	848	116.3279	848	85.57152
847	166.7463	847	141.5124	847	115.3479	847	84.78162
846	166.7128	846	141.5213	846	114.2582	846	83.90242
845	166.658	845	141.5223	845	113.1109	845	83.01148
844	166.5808	844	141.5136	844	112.0058	844	82.25737
843	166.4876	843	141.4991	843	111.1227	843	81.84764
842	166.3994	842	141.4858	842	110.6757	842	81.95572
841	166.3455	841	141.4768	841	110.797	841	82.60034
840	166.3462	840	141.4691	840	111.4518	840	83.61302
839	166.3994	839	141.4609	839	112.4541	839	84.72892
838	166.4842	838	141.4552	838	113.5614	838	85.72437
837	166.5758	837	141.4545	837	114.58	837	86.49698
836	166.6593	836	141.4565	836	115.4139	836	87.04932
835	166.7298	835	141.4572	835	116.0495	835	87.42792
834	166.7872	834	141.4553	834	116.5168	834	87.68157
833	166.8306	833	141.4502	833	116.8584	833	87.84938
832	166.8596	832	141.4392	832	117.1148	832	87.96034
831	166.8773	831	141.4207	831	117.3176	831	88.03627
830	166.8923	830	141.3979	830	117.4859	830	88.09612
829	166.9142	829	141.3764	829	117.6289	829	88.15629
828	166.9463	828	141.3603	828	117.7567	828	88.22693
827	166.9839	827	141.3512	827	117.8872	827	88.31061
826	167.0189	826	141.3482	826	118.0363	826	88.40298
825	167.0458	825	141.3452	825	118.1991	825	88.49227
824	167.0621	824	141.3326	824	118.3495	824	88.56263
823	167.0667	823	141.306	823	118.4643	823	88.60515
822	167.0601	822	141.2699	822	118.5413	822	88.62473
821	167.046	821	141.2322	821	118.5932	821	88.63185
820	167.0312	820	141.1966	820	118.6347	820	88.63238
819	167.0217	819	141.1652	819	118.6781	819	88.63027
818	167.0197	818	141.1416	818	118.7315	818	88.63305
817	167.0242	817	141.1288	817	118.7931	817	88.64581
816	167.0322	816	141.125	816	118.8525	816	88.6629
815	167.0412	815	141.1237	815	118.901	815	88.67339
814	167.0494	814	141.117	814	118.9396	814	88.67339
813	167.0561	813	141.0993	813	118.9732	813	88.66717
812	167.0604	812	141.0713	812	119.0048	812	88.65842
811	167.0603	811	141.0403	811	119.0369	811	88.64826
810	167.0534	810	141.0146	810	119.0731	810	88.64087
809	167.0419	809	140.9967	809	119.1144	809	88.64279

808	167.0336	808	140.9837	808	119.1581	808	88.65502
807	167.0359	807	140.9725	807	119.2026	807	88.67093
806	167.0473	806	140.9611	806	119.2477	806	88.68313
805	167.057	805	140.9456	805	119.2882	805	88.68869
804	167.0555	804	140.923	804	119.3152	804	88.68825
803	167.0451	803	140.8974	803	119.3289	803	88.6847
802	167.0355	802	140.8755	802	119.3406	802	88.68189
801	167.0318	801	140.8576	801	119.3586	801	88.68094
800	167.0317	800	140.8378	800	119.3794	800	88.67947
799	167.0336	799	140.8161	799	119.3994	799	88.67836
798	167.042	798	140.7999	798	119.4244	798	88.68517
797	167.0606	797	140.7909	797	119.4593	797	88.70464
796	167.0836	796	140.7784	796	119.4978	796	88.72879
795	167.1008	795	140.7523	795	119.5302	795	88.74387
794	167.1079	794	140.7172	794	119.5565	794	88.7461
793	167.1095	793	140.686	793	119.5821	793	88.7435
792	167.1117	792	140.6634	792	119.6071	792	88.74236
791	167.1156	791	140.6443	791	119.629	791	88.73937
790	167.1186	790	140.6242	790	119.6495	790	88.72811
789	167.119	789	140.6043	789	119.6718	789	88.70804
788	167.1179	788	140.5867	788	119.6952	788	88.6858
787	167.1183	787	140.5702	787	119.7197	787	88.67109
786	167.1215	786	140.5514	786	119.7495	786	88.67068
785	167.1251	785	140.5292	785	119.7842	785	88.68185
784	167.1252	784	140.5051	784	119.815	784	88.69263
783	167.1216	783	140.4834	783	119.8366	783	88.69308
782	167.1181	782	140.4673	782	119.8573	782	88.68508
781	167.1174	781	140.4552	781	119.8864	781	88.67719
780	167.1185	780	140.4415	780	119.9192	780	88.67168
779	167.1178	779	140.4222	779	119.9447	779	88.66245
778	167.1116	778	140.3988	778	119.9621	778	88.64409
777	167.0964	777	140.3751	777	119.9793	777	88.61714
776	167.0687	776	140.3526	776	119.9982	776	88.58453
775	167.0305	775	140.3295	775	120.013	775	88.54881
774	166.9911	774	140.302	774	120.0211	774	88.51422
773	166.9633	773	140.2665	773	120.0259	773	88.48751
772	166.9555	772	140.2244	772	120.0319	772	88.47557
771	166.9683	771	140.1846	771	120.0414	771	88.48257
770	166.9949	770	140.1568	770	120.0544	770	88.50629
769	167.0244	769	140.1415	769	120.0654	769	88.53531
768	167.0483	768	140.1295	768	120.0662	768	88.55497
767	167.0681	767	140.1125	767	120.0595	767	88.56127
766	167.093	766	140.0892	766	120.0594	766	88.56553
765	167.1282	765	140.0615	765	120.0729	765	88.5793
764	167.1665	764	140.0313	764	120.0886	764	88.59775

763	167.1952	763	140.0004	763	120.0927	763	88.60459
762	167.2085	762	139.9698	762	120.0885	762	88.59094
761	167.2089	761	139.9374	761	120.0879	761	88.56292
760	167.2004	760	139.9002	760	120.0915	760	88.53265
759	167.1853	759	139.8615	759	120.0918	759	88.50847
758	167.167	758	139.8282	758	120.0887	758	88.49219
757	167.1495	757	139.8011	757	120.0891	757	88.47869
756	167.1341	756	139.773	756	120.0957	756	88.45921
755	167.1191	755	139.7408	755	120.1075	755	88.43126
754	167.1032	754	139.7112	754	120.1283	754	88.40317
753	167.0858	753	139.6904	753	120.1585	753	88.38218
752	167.0648	752	139.6753	752	120.1868	752	88.36159
751	167.0384	751	139.6582	751	120.2011	751	88.32847
750	167.007	750	139.635	750	120.2041	750	88.28158
749	166.972	749	139.6046	749	120.2069	749	88.23283
748	166.9332	748	139.5673	748	120.2136	748	88.19113
747	166.8888	747	139.5262	747	120.2216	747	88.15231
746	166.8371	746	139.4875	746	120.2327	746	88.10631
745	166.7741	745	139.4533	745	120.2496	745	88.04592
744	166.6932	744	139.4197	744	120.2639	744	87.96696
743	166.5932	743	139.3854	743	120.2664	743	87.86832
742	166.4814	742	139.3575	742	120.2635	742	87.75345
741	166.3607	741	139.3398	741	120.2674	741	87.62352
740	166.2159	740	139.3242	740	120.2745	740	87.46893
739	166.0221	739	139.299	739	120.2731	739	87.2763
738	165.7639	738	139.2632	738	120.2688	738	87.04639
737	165.4425	737	139.2249	737	120.2795	737	86.79852
736	165.0685	736	139.188	736	120.3057	736	86.55732
735	164.6679	735	139.1513	735	120.3266	735	86.34535
734	164.2985	734	139.116	734	120.3301	734	86.18905
733	164.0406	733	139.0852	733	120.3227	733	86.11623
732	163.951	732	139.0548	732	120.311	732	86.13735
731	164.0215	731	139.0184	731	120.2976	731	86.23487
730	164.1847	730	138.9788	730	120.2951	730	86.37482
729	164.3587	729	138.9406	729	120.3055	729	86.52252
728	164.5066	728	138.9054	728	120.3132	728	86.66359
727	164.6604	727	138.8767	727	120.3131	727	86.81452
726	164.8772	726	138.8555	726	120.3143	726	86.99694
725	165.1755	725	138.8366	725	120.3251	725	87.20628
724	165.5133	724	138.8145	724	120.3434	724	87.41026
723	165.8234	723	138.7895	723	120.3625	723	87.5762
722	166.0584	722	138.7636	722	120.3783	722	87.69227
721	166.2071	721	138.734	721	120.3846	721	87.76323
720	166.2886	720	138.6974	720	120.3741	720	87.79875
719	166.335	719	138.6606	719	120.3538	719	87.81007

718	166.366	718	138.6298	718	120.3381	718	87.80439
717	166.3812	717	138.5972	717	120.3263	717	87.78186
716	166.3724	716	138.5529	716	120.3114	716	87.74387
715	166.3379	715	138.4999	715	120.2976	715	87.69909
714	166.2845	714	138.4505	714	120.2947	714	87.65706
713	166.2211	713	138.409	713	120.3009	713	87.61865
712	166.1577	712	138.3717	712	120.3093	712	87.58225
711	166.1079	711	138.3352	711	120.3203	711	87.55408
710	166.0862	710	138.3	710	120.3369	710	87.54563
709	166.0988	709	138.2628	709	120.3469	709	87.5584
708	166.1433	708	138.2197	708	120.3345	708	87.58425
707	166.2189	707	138.1803	707	120.3191	707	87.62456
706	166.3166	706	138.15	706	120.321	706	87.68067
705	166.4169	705	138.1197	705	120.3291	705	87.74174
704	166.5102	704	138.0872	704	120.3358	704	87.79874
703	166.5997	703	138.0609	703	120.3506	703	87.85053
702	166.6869	702	138.0436	702	120.3755	702	87.89771
701	166.7648	701	138.0262	701	120.3941	701	87.93719
700	166.8248	700	138.0006	700	120.3896	700	87.96397
699	166.8714	699	137.9762	699	120.3788	699	87.98347
698	166.9104	698	137.9592	698	120.3784	698	88.0007
697	166.9405	697	137.9394	697	120.376	697	88.01247
696	166.9681	696	137.9138	696	120.3679	696	88.02125
695	167.0051	695	137.8907	695	120.369	695	88.03325
694	167.0562	694	137.8737	694	120.391	694	88.0498
693	167.1102	693	137.8502	693	120.4174	693	88.06224
692	167.1522	692	137.8086	692	120.4203	692	88.06188
691	167.1853	691	137.7629	691	120.4052	691	88.05746
690	167.218	690	137.7297	690	120.3892	690	88.05948
689	167.2469	689	137.7031	689	120.3712	689	88.0646
688	167.2666	688	137.6729	688	120.3534	688	88.0679
687	167.2823	687	137.6453	687	120.3532	687	88.07433
686	167.3069	686	137.6322	686	120.3828	686	88.09294
685	167.341	685	137.6259	685	120.4239	685	88.11946
684	167.3707	684	137.6069	684	120.449	684	88.13829
683	167.3883	683	137.5753	683	120.4622	683	88.14515
682	167.3981	682	137.5459	682	120.4835	682	88.14843
681	167.4011	681	137.5199	681	120.5021	681	88.1493
680	167.3939	680	137.4876	680	120.496	680	88.14124
679	167.3858	679	137.4573	679	120.4842	679	88.13108
678	167.3834	678	137.4371	678	120.486	678	88.12367
677	167.3772	677	137.414	677	120.4769	677	88.10824
676	167.3745	676	137.3926	676	120.4734	676	88.0941
675	167.3691	675	137.37	675	120.4725	675	88.07761
674	167.3806	674	137.3673	674	120.5324	674	88.08396

673	167.4424	673	137.4009	673	120.7092	673	88.14608
672	167.534	672	137.422	672	120.9431	672	88.22838
671	167.6025	671	137.3683	671	121.0958	671	88.2559
670	167.7213	670	137.3548	670	121.3645	670	88.30582
669	168.121	669	137.7227	669	122.3359	669	88.64996
668	168.4845	668	138.1934	668	123.3094	668	88.98613
667	168.3509	667	138.1852	667	122.9741	667	88.88857
666	168.1625	666	138.0359	666	122.4646	666	88.77991
665	167.9794	665	137.801	665	121.921	665	88.63772
664	167.7673	664	137.4981	664	121.3102	664	88.41236
663	167.5923	663	137.2408	663	120.8005	663	88.20044
662	167.514	662	137.1114	662	120.5645	662	88.09684
661	167.4947	661	137.0626	661	120.5196	661	88.07595
660	167.4836	660	137.0267	660	120.5156	660	88.07618
659	167.4815	659	137.0037	659	120.5132	659	88.08823
658	167.4907	658	137.0006	658	120.5203	658	88.1099
657	167.4814	657	136.9828	657	120.4941	657	88.10645
656	167.4689	656	136.9576	656	120.4707	656	88.08872
655	167.477	655	136.9483	655	120.4838	655	88.07978
654	167.5005	654	136.9548	654	120.5178	654	88.08244
653	167.5171	653	136.9589	653	120.5448	653	88.08688
652	167.5194	652	136.9522	652	120.5639	652	88.08967
651	167.5159	651	136.9416	651	120.5897	651	88.09323
650	167.5134	650	136.9332	650	120.6284	650	88.09581
649	167.5018	649	136.9148	649	120.6461	649	88.08167
648	167.4809	648	136.8856	648	120.6241	648	88.04993
647	167.4718	647	136.8694	647	120.602	647	88.0262
646	167.4791	646	136.8703	646	120.6039	646	88.02145
645	167.4879	645	136.8696	645	120.618	645	88.02515
644	167.4856	644	136.8537	644	120.626	644	88.02609
643	167.4708	643	136.8241	643	120.6188	643	88.02132
642	167.4526	642	136.7914	642	120.6067	642	88.01635
641	167.4395	641	136.7627	641	120.5973	641	88.01229
640	167.435	640	136.7437	640	120.5852	640	88.00322
639	167.4421	639	136.7447	639	120.5767	639	87.98913
638	167.4549	638	136.761	638	120.5818	638	87.97364
637	167.4587	637	136.7672	637	120.5927	637	87.95716
636	167.4498	636	136.748	636	120.5995	636	87.94481
635	167.4386	635	136.7124	635	120.5996	635	87.94382
634	167.4349	634	136.6786	634	120.5977	634	87.95475
633	167.4385	633	136.6545	633	120.6002	633	87.96972
632	167.4401	632	136.6347	632	120.6019	632	87.97621
631	167.4336	631	136.6129	631	120.5916	631	87.9673
630	167.4248	630	136.5897	630	120.5704	630	87.94867
629	167.4229	629	136.5675	629	120.5474	629	87.92937

628	167.4316	628	136.5499	628	120.5321	628	87.91425
627	167.4464	627	136.5414	627	120.5359	627	87.90521
626	167.4516	626	136.5337	626	120.5482	626	87.89673
625	167.4413	625	136.5194	625	120.5584	625	87.88838
624	167.4246	624	136.5009	624	120.562	624	87.88143
623	167.4105	623	136.4817	623	120.5512	623	87.86893
622	167.4025	622	136.4622	622	120.5283	622	87.84553
621	167.4032	621	136.4449	621	120.5123	621	87.81966
620	167.412	620	136.4348	620	120.5165	620	87.80453
619	167.4213	619	136.4308	619	120.5293	619	87.80276
618	167.4241	618	136.4221	618	120.5289	618	87.80844
617	167.4287	617	136.4031	617	120.5145	617	87.82186
616	167.4483	616	136.3865	616	120.5108	616	87.8477
615	167.4733	615	136.3791	615	120.5149	615	87.86992
614	167.4807	614	136.3713	614	120.5044	614	87.86243
613	167.4688	613	136.3578	613	120.4889	613	87.82361
612	167.457	612	136.3452	612	120.4919	612	87.77526
611	167.4594	611	136.3417	611	120.512	611	87.7393
610	167.4702	610	136.3461	610	120.5305	610	87.72589
609	167.4723	609	136.3471	609	120.5375	609	87.73289
608	167.4537	608	136.3326	608	120.5308	608	87.74605
607	167.4167	607	136.2996	607	120.5102	607	87.74564
606	167.3733	606	136.2522	606	120.4751	606	87.72174
605	167.3451	605	136.2062	605	120.454	605	87.69526
604	167.3371	604	136.1694	604	120.4646	604	87.68664
603	167.3207	603	136.1227	603	120.4644	603	87.67417
602	167.2709	602	136.051	602	120.4126	602	87.62553
601	167.2097	601	135.9823	601	120.3482	601	87.55677
600	167.1832	600	135.9661	600	120.3397	600	87.51902
599	167.2015	599	136.0108	599	120.3887	599	87.5303
598	167.2296	598	136.071	598	120.4346	598	87.55395
597	167.2363	597	136.1029	597	120.4438	597	87.55252
596	167.2207	596	136.1037	596	120.4332	596	87.52659
595	167.1903	595	136.089	595	120.4187	595	87.49379
594	167.1449	594	136.0642	594	120.3964	594	87.46016
593	167.0916	593	136.0331	593	120.3738	593	87.42524
592	167.0506	592	136.0145	592	120.3789	592	87.39645
591	167.0311	591	136.023	591	120.4191	591	87.38028
590	167.0183	590	136.0437	590	120.4656	590	87.37236
589	167.0018	589	136.0523	589	120.4967	589	87.37001
588	166.9941	588	136.0471	588	120.5162	588	87.37852
587	167.0055	587	136.0399	587	120.5251	587	87.39433
586	167.0221	586	136.0315	586	120.5118	586	87.39923
585	167.0245	585	136.013	585	120.474	585	87.38427
584	167.0149	584	135.9854	584	120.4218	584	87.3646

583	167.0095	583	135.9604	583	120.3627	583	87.35823
582	167.0113	582	135.9416	582	120.3007	582	87.3611
581	167.0113	581	135.9224	581	120.2548	581	87.35852
580	167.0086	580	135.9013	580	120.2466	580	87.35034
579	167.0156	579	135.8859	579	120.2746	579	87.35199
578	167.0433	578	135.8782	578	120.3164	578	87.37213
577	167.0954	577	135.8708	577	120.3584	577	87.40503
576	167.1702	576	135.8585	576	120.4036	576	87.4415
575	167.2525	575	135.8385	575	120.4429	575	87.4751
574	167.316	574	135.8026	574	120.4512	574	87.50229
573	167.3473	573	135.7436	573	120.4225	573	87.52282
572	167.3572	572	135.6695	572	120.3796	572	87.53617
571	167.3653	571	135.5982	571	120.3413	571	87.53628
570	167.3798	570	135.5413	570	120.3084	570	87.52066
569	167.3969	569	135.4993	569	120.2802	569	87.50375
568	167.415	568	135.4761	568	120.2742	568	87.50969
567	167.4387	567	135.4782	567	120.3032	567	87.5419
566	167.4708	566	135.4956	566	120.3483	566	87.5732
565	167.5119	565	135.5067	565	120.3829	565	87.57997
564	167.5622	564	135.5017	564	120.4013	564	87.57058
563	167.6153	563	135.4878	563	120.4126	563	87.57168
562	167.654	562	135.4705	562	120.4177	562	87.59425
561	167.6651	561	135.4488	561	120.4131	561	87.62634
560	167.6545	560	135.4278	560	120.4071	560	87.65342
559	167.6395	559	135.4213	559	120.4103	559	87.67188
558	167.6307	558	135.4346	558	120.4164	558	87.68451
557	167.6273	557	135.4588	557	120.4118	557	87.69426
556	167.6261	556	135.4826	556	120.3935	556	87.7003
555	167.6257	555	135.4972	555	120.3683	555	87.69647
554	167.6261	554	135.4946	554	120.343	554	87.67918
553	167.6279	553	135.4743	553	120.3216	553	87.65777
552	167.6345	552	135.451	552	120.3071	552	87.64892
551	167.6466	551	135.4416	551	120.2977	551	87.65677
550	167.6584	550	135.4438	550	120.2876	550	87.66541
549	167.66	549	135.4392	549	120.2737	549	87.657
548	167.6476	548	135.422	548	120.2592	548	87.6327
547	167.6268	547	135.4098	547	120.2503	547	87.60854
546	167.6082	546	135.4192	546	120.2533	546	87.59473
545	167.5984	545	135.4441	545	120.27	545	87.58561
544	167.596	544	135.4654	544	120.2914	544	87.56975
543	167.598	543	135.4758	543	120.2989	543	87.54515
542	167.6076	542	135.4844	542	120.2788	542	87.52342
541	167.6312	541	135.5048	541	120.2366	541	87.51931
540	167.6637	540	135.5385	540	120.1861	540	87.53027
539	167.6832	539	135.5669	539	120.1246	539	87.5261

538	167.6696	538	135.5666	538	120.0319	538	87.46993
537	167.6294	537	135.539	537	119.8992	537	87.3558
536	167.5872	536	135.514	536	119.7371	536	87.2145
535	167.5599	535	135.5168	535	119.5576	535	87.08198
534	167.5481	534	135.5415	534	119.3571	534	86.969
533	167.5549	533	135.5736	533	119.1394	533	86.8727
532	167.5855	532	135.6145	532	118.9221	532	86.79786
531	167.6254	531	135.6683	531	118.7293	531	86.75504
530	167.6399	530	135.7195	530	118.5882	530	86.74503
529	167.6076	529	135.7446	529	118.5291	529	86.76028
528	167.5384	528	135.7353	528	118.57	528	86.79901
527	167.4595	527	135.7035	527	118.7043	527	86.86963
526	167.3962	526	135.6696	526	118.8987	526	86.97601
525	167.3651	525	135.651	525	119.1081	525	87.10477
524	167.3751	524	135.658	524	119.3124	524	87.23889
523	167.4233	523	135.6893	523	119.5098	523	87.36742
522	167.489	522	135.7287	522	119.6808	522	87.4752
521	167.5446	521	135.7576	521	119.7917	521	87.54183
520	167.5741	520	135.7718	520	119.828	520	87.55808
519	167.5821	519	135.7768	519	119.812	519	87.54024
518	167.584	518	135.7745	518	119.7897	518	87.52513
517	167.594	517	135.7703	517	119.8016	517	87.54395
516	167.6173	516	135.7845	516	119.8533	516	87.59359
515	167.6446	515	135.8273	515	119.9033	515	87.63669
514	167.6626	514	135.8814	514	119.9025	514	87.63974
513	167.6695	513	135.9204	513	119.8399	513	87.60457
512	167.6771	512	135.9306	512	119.7377	512	87.56004
511	167.6954	511	135.9138	511	119.6296	511	87.53293
510	167.72	510	135.8823	510	119.5448	510	87.52893
509	167.7373	509	135.8586	509	119.5089	509	87.54148
508	167.7332	508	135.8632	508	119.5294	508	87.56065
507	167.7017	507	135.8905	507	119.5929	507	87.57532
506	167.6502	506	135.9019	506	119.6742	506	87.57909
505	167.5966	505	135.8709	505	119.7551	505	87.58269
504	167.5579	504	135.824	504	119.8337	504	87.6099
503	167.5385	503	135.8109	503	119.8976	503	87.6676
502	167.5352	502	135.8521	502	119.9384	502	87.73284
501	167.551	501	135.9223	501	119.9815	501	87.77926
500	167.5933	500	135.9934	500	120.0499	500	87.80763
499	167.6584	499	136.0633	499	120.1327	499	87.83488
498	167.7175	498	136.124	498	120.1876	498	87.86172
497	167.7356	497	136.1538	497	120.1878	497	87.87614
496	167.7092	496	136.1477	496	120.156	496	87.87663
495	167.6713	495	136.1269	495	120.1324	495	87.87259
494	167.6584	494	136.1158	494	120.135	494	87.86963

	493	167.6756	493	136.1232	493	120.1532	493	87.86317
	492	167.6972	492	136.1484	492	120.16	492	87.84657
	491	167.7003	491	136.1868	491	120.1435	491	87.8228
	490	167.6882	490	136.2203	490	120.1112	490	87.80168
	489	167.6827	489	136.2351	489	120.0872	489	87.79904
	488	167.6972	488	136.2418	488	120.0848	488	87.82853
	487	167.7257	487	136.2649	487	120.0959	487	87.88855
	486	167.7552	486	136.3118	486	120.1142	486	87.95658
	485	167.7797	485	136.3633	485	120.1481	485	88.00178
	484	167.7968	484	136.4016	484	120.1958	484	88.01118
	483	167.7952	483	136.4317	483	120.2316	483	88.00269
	482	167.7605	482	136.461	482	120.2393	482	88.00508
	481	167.6961	481	136.4809	481	120.2325	481	88.02605
	480	167.6292	480	136.4853	480	120.2349	480	88.04747
	479	167.5923	479	136.4846	479	120.2549	479	88.04403
	478	167.5963	478	136.4853	478	120.2717	478	88.00143
	477	167.6294	477	136.4805	477	120.264	477	87.93148
	476	167.6804	476	136.4694	476	120.2407	476	87.86802
	475	167.7442	475	136.4669	475	120.2298	475	87.84522
	474	167.7968	474	136.4802	474	120.2316	474	87.87454
	473	167.7955	473	136.5009	473	120.2262	473	87.94035
	472	167.7305	472	136.5198	472	120.2169	472	88.00225
	471	167.6548	471	136.5239	471	120.2188	471	88.01414
	470	167.6142	470	136.4947	470	120.2192	470	87.96412
	469	167.603	469	136.4379	469	120.2105	469	87.88443
	468	167.5923	468	136.3901	468	120.1947	468	87.8165
	467	167.566	467	136.3866	467	120.179	467	87.78315
	466	167.5329	466	136.4133	466	120.1713	466	87.77927
	465	167.5107	465	136.4315	465	120.1881	465	87.80046
	464	167.5014	464	136.4373	464	120.2413	464	87.83877
	463	167.4808	463	136.4527	463	120.2982	463	87.87406
	462	167.4229	462	136.4713	462	120.306	462	87.8395
	461	167.3334	461	136.4538	461	120.2536	461	87.76351
	460	167.2427	460	136.3992	460	120.1991	460	87.61748
	459	167.1339	459	136.3759	459	120.1985	459	87.1466
	458	166.8876	458	136.46	458	120.2373	458	86.87833
	457	166.413	457	136.6185	457	120.2703	457	86.51498
	456	165.8424	456	136.7315	456	120.29	456	86.15899
	455	165.4037	455	136.7625	455	120.3229	455	85.85792
--			454	136.7604	454	120.3703		
--			453	136.7716	453	119.8581		
--			452	136.8078	452	119.9639		
--			451	136.8418	451	120.1889		
--			450	136.835	450	120.3537		
