# Supplementary material for: Understanding the impact of crosslinked PCL/PEG/GelMA electrospun nanofibers on bactericidal activity
Source: PLoS One. 2018 Dec 20;13(12):e0209386. doi: 10.1371/journal.pone.0209386 (PMC6301679; doi:10.1371/journal.pone.0209386)
Supplement: S2 Table — The raw data for the bacteria test presented in Fig 3. (PDF) [file pone.0209386.s002.pdf]

Bacteria test raw data

Bacteria test

S. aureus

Sample name	Bacteria density	SD	N
PCL	248571.4286	22677.86838	3
PCL:PEG	442500	72456.88373	3
PCL:PEG:GELMA	1.63E+06	413118.2236	3
PCL:PEG:GELMA-UV	125000	37815.3408	3

P. aeruginosa

Sample name	Bacteria density	SD	N
PCL	7.00E+07	1.53E+07	3
PCL:PEG	4.60E+07	1.34E+07	3
PCL:PEG:GELMA	6.75E+07	1.26E+07	3
PCL:PEG:GELMA-UV	5.00E+06	1.41E+06	3

MRSA

Sample name	Bacteria density	SD	N
PCL	2.44E+06	296647.9395	3
PCL:PEG	2.22E+07	1.64E+06	3
PCL:PEG:GELMA	2.43E+06	975900.073	3
PCL:PEG:GELMA-UV	2.29E+06	967230.9519	3
