# Supplementary material for: Understanding the impact of crosslinked PCL/PEG/GelMA electrospun nanofibers on bactericidal activity
Source: PLoS One. 2018 Dec 20;13(12):e0209386. doi: 10.1371/journal.pone.0209386 (PMC6301679; doi:10.1371/journal.pone.0209386)
Supplement: S3 Table — The raw data for the Hydrogen peroxide assay, BCA protein assay, Reactive oxygen species (ROS) assay, Superoxide assay and Cell viability test presented in Fig 5. (PDF) [file pone.0209386.s003.pdf]

Raw data

Hydrogen peroxide assay

PCL:PEG:GelMA	PCL:PEG:GelMA-UV	PCL:PEG:GelMA
2.022542	1.705995	3.902638
2.626858	1.917026	3.931415
3.648441	1.854676	3.1832

BCA protein assay

PCL:PEG:GelMA	PCL:PEG:GelMA-UV	PCL:PEG:GelMA	PCL:PEG:GelMA-UV
41.05	53.5	122.1	121.35
38.65	62.25	129.9	160.3
43.45	61.2	142.65	141.55

Reactive oxygen species (ROS) assay

Sample name		SD	N
		**P. aeruginosa**	
PCL:PEG:GelMA	18.13641	0.5345776	3
PCL:PEG:GelMA-UV	40.17823	17.35631	3
		**MRSA**	
PCL:PEG:GelMA	57.3346	8.378231	3
PCL:PEG:GelMA-UV	44.99643	7.147259	3
		**S. aureus**	
PCL:PEG:GelMA	54.94762	10.70661	3
PCL:PEG:GelMA-UV	83.86202	23.0011	3

Superoxide assay

Sample name		SD	N
		**P. aeruginosa**	
PCL:PEG:GelMA	14.99222	0.6232161	3
PCL:PEG:GelMA-UV	18.00027	3.850022	3
		**MRSA**	
PCL:PEG:GelMA	70.75477	23.21392	3
PCL:PEG:GelMA-UV	46.86355	8.606808	3
		**S. aureus**	
PCL:PEG:GelMA	22.21775	1.683256	3
PCL:PEG:GelMA-UV	16.96426	0.7768585	3

Cell viability test

Sample name	Mean Cell viability (%)	SD	N
PCL	41.64871	0.86579	3
PCL:PEG	103.9991	8.36931	3
PCL:PEG:GelMA	114.4626	10.0485	3
PCL:PEG:GelMA-UV	86.65741	4.01791	3
Control	100	3.68974	3
